# MYC2 influences rubber and sesquiterpene lactones synthesis in Taraxacum species

**DOI:** 10.1007/s00425-025-04719-9

**Published:** 2025-05-24

**Authors:** Elio Fantini, Loretta Daddiego, Paolo Facella, Giorgio Perrella, Linda Bianco, Carlo Fasano, Fiammetta Alagna, Michele Antonio Savoia, Daniela Rigano, Carmina Sirignano, Orazio Taglialatela Scafati, Severina Pacifico, Simona Piccolella, Loredana Lopez, Francesco Panara

**Affiliations:** 1 Trisaia Research Center, ENEA, S.S. 106 Ionica - Km 419+500, 75026 Rotondella, MT Italy; 2 Department of Biosciences, University of Milan, Via Celoria 26, 20133 Milan, Italy; 3 Department of Soil, Plant and Food Sciences, University of Bari Aldo Moro, Via Amendola 165/A, 70126 Bari, Italy; 4 Department of Pharmacy, School of Medicine and Surgery, University of Naples Federico II, Via D. Montesano 49, 80131 Naples, Italy; 5 Department of Environmental Biological and Pharmaceutical Sciences and Technologies, University of Campania “Luigi Vanvitelli”, Via Vivaldi 43, 81100 Caserta, Italy

**Keywords:** *Taraxacum kok-saghyz*, MYC2, Metabolites, Rubber, Jasmonate signalling, Sesquiterpene lactone

## Abstract

**Main conclusion:**

This study showed that MYC2 transcriptionally regulates valuable metabolites in *Taraxacum spp*. through direct interaction with specific target gene promoters.

**Abstract:**

The Russian dandelion (*Taraxacum kok-saghyz*) represents a promising alternative species, capable of producing several high-added-value compounds, including natural rubber. Nevertheless, further enhancements are required for its optimal utilization by the industry. Here, we explored the role of the bHLH transcription factor TksMYC2, homolog of AtMYC2, in the regulation of the biosynthesis of specialized metabolites and free fatty acids and in the control of natural rubber production. Metabolic analyses of *Taraxacum kok-saghyz* plants showed that the overexpression of TksMYC2 significantly affected the accumulation of metabolites in roots and leaves, such as sesquiterpene lactones, phenylpropanoids, and free fatty acids. Moreover, overexpressing plants presented a significant increase in natural rubber production in both *Taraxacum kok-saghyz* and its related species *Taraxacum brevicorniculatum*. The direct interaction of TksMYC2 with the regulatory regions of cis-prenyltransferase 2 (*CPT2*), small rubber particle proteins (*SRPP1*, *SRPP3,* and *SRPP4*), involved in the biosynthesis of natural rubber, and with the germacrene A oxidase (*GAO*), involved in the biosynthesis of sesquiterpenes, was demonstrated by chromatin immunoprecipitation coupled with quantitative PCR. Additionally, these genes were highly induced in the lines overexpressing *TksMYC2*. Our findings suggest that TksMYC2 and its downstream components may be valid targets for breeding programmes to increase the production of valuable metabolites, including natural rubber.

**Supplementary Information:**

The online version contains supplementary material available at 10.1007/s00425-025-04719-9.

## Introduction

The Russian dandelion (*Taraxacum kok-saghyz* Rodin, Tks) is a perennial herbaceous dicotyledonous plant belonging to the *Asteraceae* family, native to Kazakhstan (Kirschner et al. 2013). The number of *Taraxacum* species is rich and diverse, but only a small proportion have been subjected to scientific studies in recent years, including *Taraxacum brevicorniculatum* (Tb), *Taraxacum officinale*, and *Taraxacum mongolicum* (Schütz et al. 2006; Laibach et al. 2015, 2018; Martinez et al. 2015). It is notable that Tb is an apomictic species, sympatric with Tks, with which it probably shares a maternal ancestor and a portion of the genome (Zhang et al. 2017). Tks has been identified as a potential alternative source of high-quality natural rubber (NR), which is abundant in its latex (van Beilen and Poirier 2007). NR (cis-1,4-polyisoprene) is a biopolymer consisting of isoprene units (C_5_H_8_)_n_ linked together in a 1,4 cis-configuration that is produced and stored in laticifer cells, pipe-like anastomosed cell systems, that produce latex (Brown et al. 2017). The isopentenyl pyrophosphate (IPP) monomer is the key precursor in natural rubber biosynthesis (Chow et al. 2012). Plants utilize two distinct pathways to synthesize IPP: the mevalonate (MVA) pathway, which occurs in the cytosol, and the methylerythritol (MEP) pathway, which takes place in the plastid (Chow et al. 2012; Yamashita and Takahashi 2020). Enzymes and proteins known to use IPP precursors to synthesize NR include farnesyl pyrophosphate synthase (FPS), small rubber particle protein (SRPP), rubber elongation factor (REF), cis-prenyltransferase (CPT), and HRT1-REF bridging protein (HRBP) (Amerik et al. 2018; Men et al. 2019; Yamashita and Takahashi 2020). A total of 102 gene and protein members related to NR biosynthesis, including 10 CPT, 8 SRPP, 2 REF, and one HRBP, were identified in the roots of Tks during their development (Xie et al. 2022).

The roots of *Taraxacum* spp*.*, besides NR, are also a rich source of various bioactive metabolites, with promising applications in several fields, particularly bioethanol production, biomedical, and food industries (Ramirez-Cadavid et al. 2017; Piccolella et al. 2023). The high carbohydrate content, particularly inulin, makes dandelion roots a valuable feedstock used in food and non-food applications. In addition to the roots, various tissues of the dandelion contain secondary metabolites of known physiological and pharmaceutical significance. These include terpenoids, such as sesquiterpene lactones (STLs) like taraxacolides, dihydrolactucin, ixerin, taraxinic acids, and ainslioside, as well as pentacyclic triterpenes such as lupeol, amyrins, and taraxasterol. Additionally, dandelions are rich in phenolic compounds, including chicoric, monocaffeoyl, tartaric, chlorogenic, and caffeic acids, as well as coumarins. Other abundant secondary metabolites include phenylpropanoids, flavonoids, and phytosterols (Schütz et al. 2006; Esatbeyoglu et al. 2017).

Tks can be grown from transplants or direct sowing, and can be cultivated as an annual crop, although it remains poorly domesticated due to a number of intrinsic factors, such as slow growth, poor competitiveness with weeds, significant rubber yield only at maturity, high degree of heterozygosity, and self-incompatibility (Kuluev et al. 2023). Therefore, both molecular and conventional breeding efforts are fundamental to improve agronomic fitness and rubber yield, before that Tks can become a major competitive rubber crop (Cherian et al. 2019; Panara et al. 2022). However, to make Tks a truly successful biorefinery feedstock, the parallel valorization of inulin and other classes of valuable metabolites is essential (Ramirez-Cadavid et al. 2017; Piccolella et al. 2023).

Despite the identification of numerous genes involved in the biosynthetic pathways of NR, inulin and other valuable metabolites (Kuluev et al. 2023), our understanding of their regulation remains limited. Several evidences suggest that Jasmonate (JA) signalling could affect the modulation of NR biosynthesis. Tks plants treated with Methyl-Jasmonate (MeJA) and analysed at 6 h and 24 h after treatment showed an induction of genes involved in NR production as well as genes involved in JA signalling, such as *JAR1* and *MYC2,* and in JA synthesis (Cao et al. 2017; Dong et al. 2023b). MeJA had similar effects in Hb, upregulating NR biosynthesis genes and enhancing rubber production (Deng et al. 2018). The analysis of RNAseq data obtained from a comparison between high and low rubber-producing plants of Tks showed that several genes involved in JA signalling were differentially expressed between the two groups. The master regulator of JA signalling, MYC2, and a contig annotated as MYC2-like, were highly expressed in high rubber-producing plants (Panara et al. 2018). In *Taraxacum antungense* (Ta), the overexpression of MYC2 induced the expression of squalene synthase gene (*TaSS*), hence triterpenes biosynthesis. Furthermore, it has been shown that TaMYC2 directly binds to the E-box motif in the promoter of *TaSS* (Liu et al. 2023). Recently, it was observed that the overexpression of TksMYC2 increases the biomass of TKS roots and stimulates the biosynthesis of Tks NR by modulating the expression of the TksSRPP/REF gene family (Wu et al. 2024).

MYC2 is a bHLH transcription factor involved in the JA-mediated regulation of plant development, stress responses, and synthesis of specialized metabolites (Kazan and Manners 2013; Song et al. 2022; Luo et al. 2023). MYC2 forms a complex with the F-box receptor COI1 and the JA signalling repressor JAZ. In *Arabidopsis thaliana* (At), MYC2 regulates gene expression through binding to the G-box motif present in promoter regions (Song et al. 2022). Additionally, it has been demonstrated that the JA active form on the MYC2 complex is the (+)-7-iso-jasmonoyl-L-isoleucine (JA-Ile). JA-Ile can bind COI1 and JAZ, causing the degradation of JAZ by the 26S proteasome. This results in the release of MYC2 activity and the activation of gene expression (Thines et al. 2007). The COI1–JAZ3–MYC2 module was also identified in laticifer cells of Hb, where it regulates HbFPS1 and HbSRPP1 determining an increase in the efficiency of natural rubber biosynthesis (Deng et al. 2018).

To investigate the potential involvement of MYC2 in the regulation of valuable metabolite biosynthesis in *Taraxacum*, including NR, we identified and functionally characterized the homolog of AtMYC2 in Tks. An in silico analysis was initially conducted to investigate the gene structure, conserved domains, phylogeny, and collinearity of the *TksMYC2* gene. Subsequently, we overexpressed the *TksMYC2* gene in both Tks and Tb. Transgenic lines analyses revealed that increased expression of *TksMYC2* affects specialized metabolite accumulation and NR production. Finally, we used ChIP-qPCR to explore the interaction of TksMYC2 with selected genes involved in the biosynthesis of specialized metabolites. This analysis showed that TksMYC2 binds G-box elements in the promoter regions of genes involved in rubber biosynthesis and sesquiterpene lactones (SLs) production.

## Materials and methods

### Plant materials and growth conditions

**Fig. 1:**
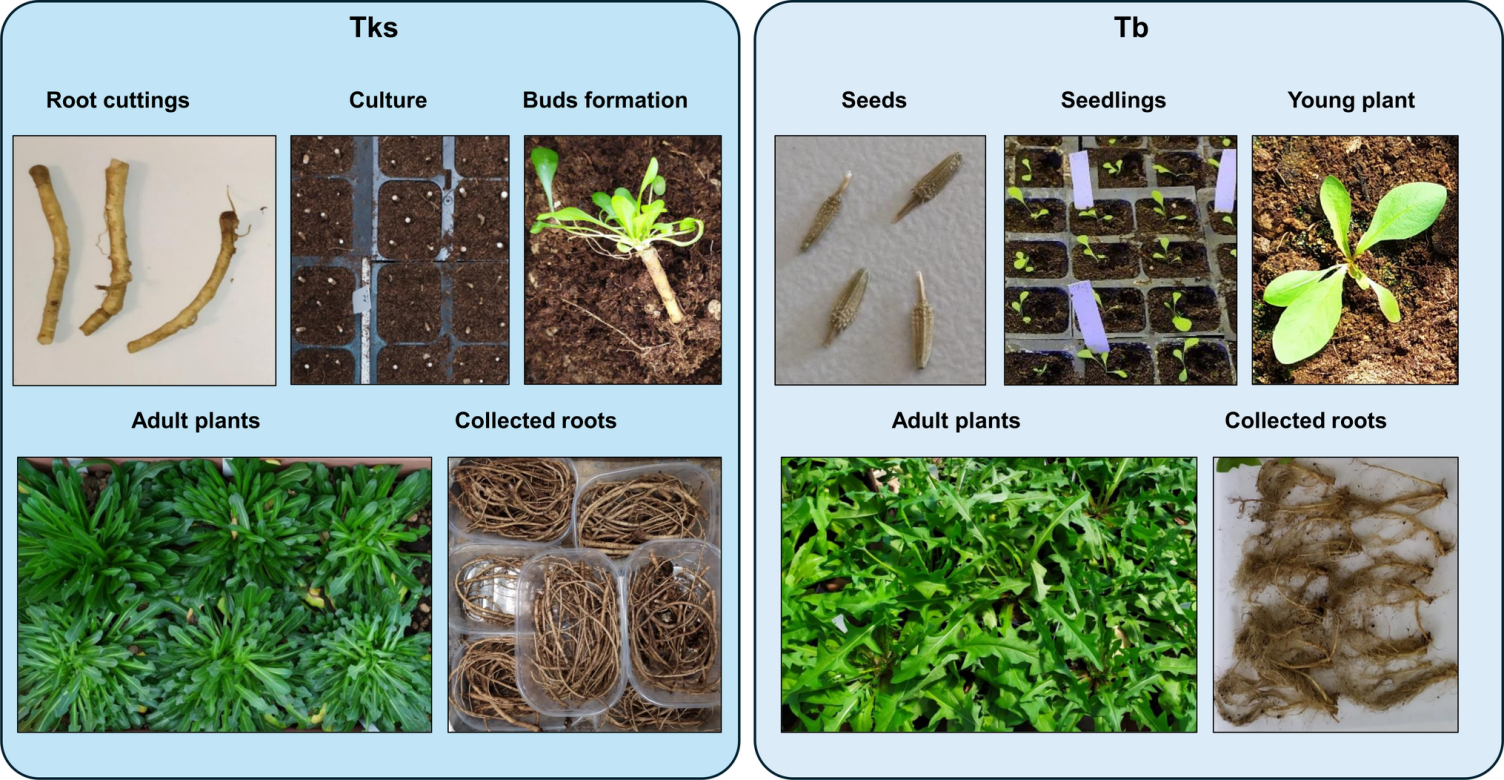
*Taraxacum kok-saghyz* (Tks) and *Taraxacum brevicorniculatum* (Tb) plant propagation

All Tks plants used in this research were derived from a plant of the high rubber accession W6 35166 (Panara et al. 2018), named Tks-G, which was selected for its high regeneration ability. Tb plants were derived from the apomictic “Clone A” (Kirschner et al. 2013) donated by Peter van Dijk (Keygene, Wageningen, The Netherlands). Tks plants were propagated by root cutting, whereas Tb plants were propagated by seed (Fig. 1). Tks cuttings and Tb seedlings were placed on standard commercial culture substrate (decomposed peat moss) with 7% horticultural sand (v/v) to improve drainage and grown in growth chambers under a 24/20 °C thermoperiod and 14/10 h photoperiod with 30 µmol m-2 s-1 blue and red-enriched white light (Osram Natura de Luxe 36 W/76). Rooted Tks clones and Tb plantlets were transplanted into 2.6 L polyethylene pots and cultivated under greenhouse conditions at the ENEA Trisaia Research Centre (40°09′47.4″N 16°38′00.1″E, Basilicata Region, Italy) from October 2022 to the end of June 2023.

### In silico analyses

The complete Tks genome sequence and transcriptomic data in tissues and organs were obtained from Lin and co-authors (Lin et al. 2022). Alignments were performed using the ClustalO program. A phylogenetic tree was constructed using the MEGA 6 software by the neighbour-joining tree method with bootstrap set to 1000. For genomic annotation and intron/exon structure prediction, the FGENESH (www.softberry.com) algorithm was employed (Solovyev et al. 2006). Hierarchical clustering was performed using MeV 4.9.0 (Saeed et al. 2003) on Log_2_ fold changes values among samples for each gene.

### Cloning and genetic transformation

Generation of plasmid for *TksMYC2* overexpression: the coding sequence of the *TksMYC2* gene was amplified using TksMYC2_BamHI_for and TksMYC2_SacI_rev primers (Table S1) and cloned from the full-length cDNA of the Tks-G plant into the BamHI and SacI sites of the *Agrobacterium*-based plant expression vector pBI121 (Jefferson 1987; Chen et al. 2003) under the control of the cauliflower mosaic virus 35S promoter, generating pBI121:*TksMYC2* (Fig. S1a).

Generation of plasmid for *TksMYC2-GFP* expression: the coding sequence without the stop codon of the *TksMYC2* gene was amplified using *TksMYC2_B1* and *TksMYC2_B2* primers (Table S1) and cloned into pDONOR221 using BP-clonase II, following the manufacturer’s instructions, and then transferred to pH7 FWG2 plasmid (Karimi et al. 2002) by recombination using LR-clonase II (Thermo Fisher) allowing the C-terminus fusion with *eGFP*, generating pH7 FWG2:*TksMYC2* (Fig. S1b)*.*

*Agrobacterium tumefaciens* strain EHA105, carrying pBI121:*TksMYC2* or pH7 FWG2:*TksMYC2*, was cultured in 50 mL of LB broth containing the appropriate antibiotics (100 mg L^−1^ kanamycin and 50 mg L^−1^ rifampicin). The bacteria were cultured at 28 °C to the end of the log phase and then centrifuged, and the pellet was resuspended in sterile ultrapure water. Leaf cuttings from Tks-G and Tb plants were sterilized and inoculated with *A. tumefaciens*. Cuttings were then placed on filter paper and successively on co-culture medium (4.3 g L^−1^ Murashige and Skoog salt solution, 30 g L^−1^ sucrose, vitamins B5 (100 mg L^−1^ myoinositol, 1 mg L^−1^ nicotinic acid, 1 mg L^−1^ pyridoxine–HCl, 10 mg L^−1^ thiamine-HCl), 1, 5 mg L^−1^ zeatin, 0, 2 mg L^−1^ indole-3-acetic acid, and 8 g L^−1^ agar, pH5.8) for 2–3 days. Then, cuttings were washed to remove any excess of *Agrobacterium* and placed on the selection/regeneration medium (4.3 g L^−1^ Murashige and Skoog salt solution, 30 g L^−1^ sucrose, B5 vitamins, 1.5 mg L^−1^ zeatin, 0.2 mg L^−1^ Indole-3-acetic acid, and 8 g L^−1^ agar, pH5.8) supplemented with 25 mg L^−1^ kanamycin and 300 mg L^−1^ augmentin, for callus and shoot induction. Root formation was induced on rooting medium (2.15 g L^−1^ Murashige and Skoog salt solution, 15 g L^−1^ sucrose, B5 vitamins, 0.2 mg L^−1^ naphthaleneacetic acid, and 8 g L^−1^ agar, pH 5.8) supplemented with 10 mg L^−1^ kanamycin and 200 mg L^−1^ augmentin. Rooted seedlings were transferred to the same medium without naphthaleneacetic acid and antibiotics. The resulting plants were transferred to soil and grown in growth chamber as described above.

To verify the presence of the transgene, DNA was extracted from leaves using the InnuPrep Plant DNA Kit (Analytik, Jena, Germany) according to the manufacturer’s protocol. DNA concentrations were estimated by Nanodrop^™^ 1000 Spectrophotometer (Thermo Fisher Scientific, Waltham, MA, USA) and amplified with transgene-specific primers (Table S1). Positive plants were further checked for transgene expression by qRT-PCR.

### Extraction and quantification of rubber, inulin, and resins from roots

Rapid extraction and quantification of the rubber were performed using the method proposed by Zhang and co-authors (Zhang et al. 2019). Root pools of at least 5 plants/genotype were used for rubber extraction. Briefly, the roots were washed with tap water and dried in an oven at 45 °C to constant weight. They were then cut into approximately 1 cm long pieces, which were ground and sieved through a 3 mm sieve. To remove the resins, the root powder was weighed and mixed with 20 mL acetone (Merck Life Science S.r.l. ACS reagent, ≥ 99.5%) per gramme of powder. The acetone-dispersed samples were then subjected to a total of 3 cycles of ultrasonic treatment using an ultrasonic cleaning bath (Falc Instruments, LBS2 10Lt). The ultrasonic power was set to 80% and the working frequency was 40 kHz; each extraction was performed for 6 min. After each treatment cycle, the samples were centrifuged at 3500 rpm for 10 min and the supernatant was discarded. For rubber extraction, the above treatment was repeated with the addition of 1:20 hexane (Merck Life Science S.r.l. ACS reagent ≥ 99%) instead of acetone. The hexane fractions were collected in a pre-weighed aluminium weighing boat, evaporated to constant weight under a fume hood, and then analysed by gravimetry.

For a more detailed characterization of the rubber, inulin, and resin content of Tks and Tb roots, the method proposed by Piccolella and co-authors (Piccolella et al. 2023) was applied. Ground roots, prepared as before, were extracted using an ASE 200 accelerated solvent extractor (Dionex Corp., Now Thermo Fisher Scientific Inc., Waltham, MA). An 11 mL stainless steel ASE extraction cell, with a cellulose filter on the bottom, was filled with 20–30 mesh inert material and 0.5 g of ground roots continuously mixed using a metal spatula. According to the optimized ASE extraction protocol of Ramirez-Cadavid and co-authors (Ramirez-Cadavid et al. 2017), water, acetone, and hexane were used to extract inulin, resins, and rubber, respectively. The optimized ASE extraction protocol started with two 40 min water extractions at 95 °C, followed by one 40 min acetone extraction at 40 °C, and three 20 min hexane extractions at 160 °C. The gravimetric determination of acetone and hexane extractables was performed by evaporation, while inulin quantification in the water extract was carried out by using a high-pressure liquid chromatography (Pari et al. 2021).

### Metabolite extraction

Tks wild-type and TksMYC2 overexpressing plants were collected and air dried at room temperature in the shade for metabolite extraction. Aerial parts and roots from 5 plants/genotype were separated and finely powdered using a blender. The powders were then sequentially extracted by cold maceration with *n*-hexane, acetone, and methanol (3 × 250 mL for all solvents) at room temperature. Three cold maceration cycles (each one for eight hours) were carried out for each solvent. After filtration, the samples were dried by a rotary evaporator operating *in vacuo* at a low temperature (35 °C). The obtained extracts were stored at −20 °C until UHPLC-HRMS/MS analysis was performed. All solvents were purchased from Merck Life Science S.r.l. (ACS reagent ≥ 99%).

### Metabolite profiling by UHPLC-HRMS and MS/MS tools

The obtained extracts from the leaves and the roots of wild-type and TksMYC2 overexpressing Tks plants were chemically profiled by using the UHPLC NEXERA (Shimadzu, Tokyo, Japan) coupled to the AB SCIEX TripleTOF^®^ 4600 mass spectrometer (AB Sciex, Concord, ON, Canada). The Luna^®^ Omega C18 column (150 × 2.1 mm, 1.6 μm; Phenomenex) as a stationary phase was exploited for all the extracts, as well as the mobile phase composed of water and acetonitrile (both containing 0.1% formic acid), and adapting the elution gradient to the complexity and polarity of the metabolites therein (Fig. S2a). The flow rate was 0.5 mL min^−1^ with an injection volume of 2.0 μL.

Metabolite profiling was carried out in negative ESI mode with automated mass calibration of both TOF–MS and MS/MS experiments. The detailed mass parameters are reported in Fig. S2b. Data analysis was performed using PeakView^®^ software version 2.2, whereas the instrument was controlled by Analyst^®^ TF 1.7 software.

The compound relative quantitation was performed taking into account the areas under the peak in extracted ion chromatograms and calculating the deviation (%) *vs.* Tks wildtype for each metabolite.

### RNA extraction and RT-qPCR

Total RNA from Tks or Tb leaves was extracted using innuPREP Plant RNA Kit (Analytik, Jena, Germany) according to the manufacturer’s protocol. RNA quality and concentration were estimated by Nanodrop^™^ 1000 Spectrophotometer (Thermo Fisher Scientific, Waltham, MA, USA). qRT-PCR was performed according to Panara and co-authors (Panara et al. 2018), and data were normalized to the amount of the housekeeping GAPC2 (glyceraldehyde-3-phosphate dehydrogenase C2) transcript. The sequences of the primers used for the RT-qPCR are listed in Table S1.

### Confocal imaging

GFP signal was assessed in tobacco epidermal cells two days after infiltration (Geelen et al. 2002). GFP fluorescence in tobacco and *Taraxacum* leaves was visualized by confocal microscopy. GFP fluorescence was excited at 488 nm with light from an argon laser and collected after passage through an NFT545 dichroic mirror with a 505 nm long-pass filter.

### Chromatin immunoprecipitation

Chromatin immunoprecipitation was performed using 5 g of tissue as previously described with minor modifications (Perrella et al. 2024). For each experiment leaves and roots from at least three plants were pooled to extract chromatin (at least 5 g of fixed tissue). A water bath sonicator was used to shear the chromatin using 40 cycles consisting each of 30 secs ON and 30 secs OFF at high power. Anti-GFP (Abcam ab290) antibody was used to immunoprecipitate the chromatin. ChIP-qPCR was performed with a 3 min initial denaturation at 95 °C followed by 40 cycles at 95 °C, 3 s, and 59.5 °C, 30 s. Reactions were performed on four technical replicates and two independent biological replicates. Relative enrichment for ChIP-qPCR assays was calculated as shown in Shapulatov and co-authors (Shapulatov et al. 2023).

## Results

### Identification of the Tks homolog of AtMYC2

**Fig. 2:**
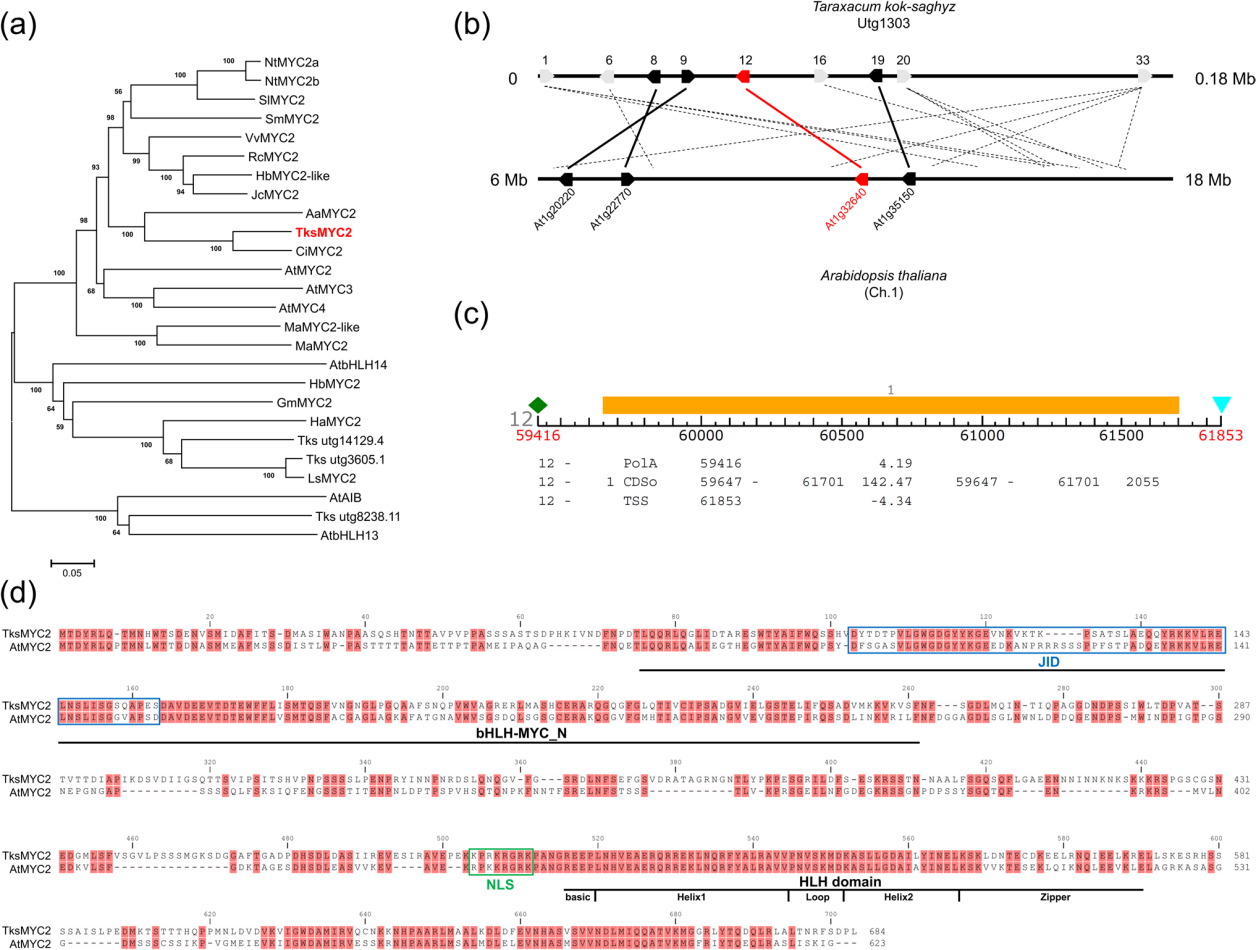
In silico analysis of AtMYC2 homolog in *Taraxacum kok-saghyz*. **a** Phylogenetic tree constructed by the neighbour-joining method with 1000 replications after ClustalO alignment of TksMYC2 and other bHLH proteins: NtMYC2a (ADU60100.1; *Nicotiana tabacum*), NtMYC2b (ADU60101.1; *Nicotiana tabacum*), SlMYC2 (NP_001311412.1; *Solanum lycopersicum*), SmMYC2 (AIO09733.1; *Salvia miltiorrhiza*), VvMYC2 (A6NAB4; *Vitis vinifera*), RcMYC2 (XP_002519814.1; *Ricinus communis*), HbMYC2-like (AJC01627.1; *Hevea brasiliensis*), JcMYC2 (XP_012076236.0; *Jatropha curcas*), AaMYC2 (AKO62850.1; *Artemisia annua*), CiMYC2 (A0A0M4JJA3; *Cichorium intybus*), AtMYC2 (Q32204.2; *Arabidopsis thaliana*), AtMYC3 (Q9 F1 T9; *Arabidopsis thaliana*), AtMYC4 (O49687.1; *Arabidopsis thaliana*), MaMYC2-like (XP_009384727.2; *Musa acuminata*), MaMYC2 (XP_009413229.2; *Musa acuminata*), AtbHLH14 (O23090; *Arabidopsis thaliana*), HbMYC2 (ADK91082.1; *Hevea brasiliensis*), GmMYC2 (XP_003528771.1; *Glycine max*), HaMYC2 (XP_022016669.1; *Helianthus annuus*), Tks utg14129.4, Tks utg3605.1, LsMYC2 (XP_023753528.1; *Lactuca sativa*), AtAIB (Q9ZPY8; *Arabidopsis thaliana*), Tks utg8238.11, AtbHLH13 (Q9LNJ5.1; *Arabidopsis thaliana*). **b** Collinearity between the Utg1303 contig and the *Arabidopsis thaliana* chromosome 1 region spanning 6 to 18 Megabases. The number refers to the genes predicted by FGENESH. In light grey are indicated probable repeated elements, in black the conserved genes, and in red TksMYC2 and AtMYC2. **c** Structure of the *TksMYC2* gene predicted by FGENESH. Positions are referred to the Utg1303 contig. TSS is the transcription start site; PolA is the polyadenylation site. **d** Alignment of TksMYC2 with MYC2 from *Arabidopsis thaliana*. bHLH-MYC-N domain and bHLH-leucine zipper domain are underlined. JAZ Interaction Domain (JID) is indicated by blue boxes. Nuclear localization signal (NLS) is indicated by green boxes

In order to identify the TksMYC2 protein sequence, the AtMYC2 protein (TAIR At1g32640, UNIPROT Q39204) was used to query the Tks genome databases (Lin et al. 2017) using blastp. The evm.model.utg1303.12 protein was selected for the highest similarity with AtMYC2 (Score = 599 bits (1545), E-value = 0.0, Identities = 51%, Positives = 65%, Gaps = 14%). Proteins with lower similarity were employed as queries in a blastp search against the NCBI nr database, resulting to be homologs to other members of the At bHLH gene family. Evm.model.utg1303.12 and the successive top three proteins resulted from blastp (evm.model.utg14129.4, evm.model.utg3605.1 and evm.model.utg8238.11) were aligned to AtMYC2 and 21 additional bHLH proteins from At and other plant species, and a phylogenetic tree was constructed (Fig. 2a). As expected, TksMYC2 is the most closely related to AtMYC2 among the selected Tks bHLHs. Additionally, TksMYC2 exhibits high similarity to two other putative MYC2 proteins within the *Asteraceae* family (*Cichorium intybus*, CiMYC2 and *Artemisia annua,* AaMYC2), but not to the putative MYC2 proteins from the rubber-producing species *Lactuca sativa* and Hb*,* which cluster with evm.model.utg3605.1 and evm.model.utg14129.4.

To further confirm whether *TksMYC2* could be the homolog of *AtMYC2*, a collinearity analysis was conducted between Tks contig utg1303 (position 59,662–61,701 in the reverse orientation) and At chromosome 1, harbouring *TksMYC2* and *AtMYC2,* respectively. The Tks contig utg1303 was reannotated using FGENESH software, resulting in the identification of 33 putative CDSs (Fig. S3). A tblastn analysis of the corresponding predicted proteins against the At genome revealed that only 15 CDSs exhibited a degree of similarity with At. The results were further filtered based on identity > 40%. As expected, one of the identified CDS (#12) correspond to *TksMYC2*, while 4 other CDSs exhibited similarities to genes in close proximity to *AtMYC2* within the At genome (Fig. 2b). CDSs #8 and #9 were located upstream of *TksMYC2* but in inverted order when compared to At, while CDS #19 was located downstream of *TksMYC2*, as observed in At. Moreover, 5 out of 7 CDSs with numerous hits, identified as probable repeated sequences, were present in one or more locations within the At genomic region containing *AtMYC2*. This result supports the hypothesis of collinearity between the two regions surrounding *MYC2* in Tks and At.

The structure of the *TksMYC2* transcript was predicted using the FGENESH software. *TksMYC2* is composed of a single exon, devoid of any introns. This characteristic of *MYC2* is observed in other plant species (Fig. 2c). CDS sequence length was confirmed by the assembly of RNAseq data (Panara et al. 2018).

Finally, the TksMYC2 and AtMYC2 protein sequences were aligned and analysed to identify the main functional domains. The bHLH-MYC-N, bHLH-leucine zipper, the Jaz Interaction domain, and the nuclear localization signal were present, exhibiting high similarity with At, indicating that the protein function and its mechanism of action may also be conserved between Tks and At (Fig. 2d).

### Expression analysis of TksMYC2

**Fig. 3:**
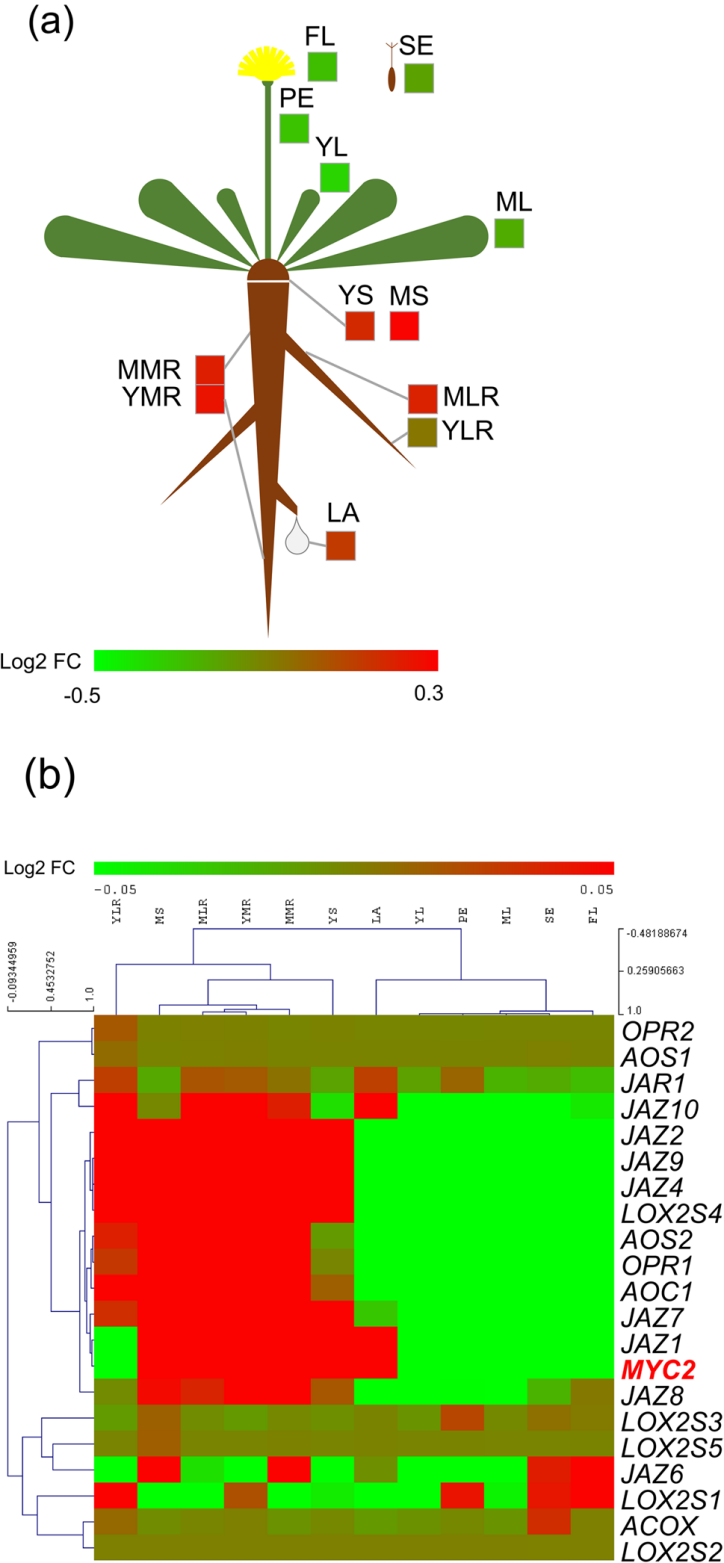
Expression analysis of *TksMYC2* transcript in plant organs. **a** Schematic representation of the Tks plant organs with corresponding Log_2_FC gene expression levels reported in coloured boxes. **b** Heatmap representing hierarchical clustering analysis of Log_2_FC gene expression levels among plant organs of genes involved in JA synthesis and signalling. *FL* flower, *SE* seed, *PE* petiole, *YL* young leaf, *ML* mature leaf, *YS* young stem, *MS* mature stem, *MMR* mature main root, *YMR* young main root, *MLR* mature lateral root, *YLR* young lateral root, *LA* latex

Previous gene expression studies have demonstrated that *TksMYC2* expression in the root is modulated between high and low rubber-producing Tks genotypes (Panara et al. 2018); additionally, its expression was induced by MeJa treatment after 6 and 24 h (Dong et al. 2023b). Analysis of gene expression data in Tks organs generated by Lin and co-authors (Lin et al. 2017) revealed that *TksMYC2* exhibited a reduced level of expression in aerial and reproductive organs (Fig. 3a). It is noteworthy that *TksMYC2* showed high level of expression in the latex, which may indicate a potential involvement in the regulation of transcripts expressed in this tissue.

It has also been shown that genes involved in JA synthesis and signalling exhibited increased expression in the roots of Tks plants at 6 and 24 h following MeJa treatment (Dong et al. 2023b). In addition, interactions between TksMYC2 and all TksJAZs were demonstrated using Y2H and BiFC assays (Wu et al. 2024). Hierarchical clustering analysis was performed to identify potential correlations between the expression patterns of the same genes in Tks organs (Fig. 3b). *TksMYC2* clustered with *TksJAZ1* and *TksJAZ7*, whose products are potential interactors of MYC2. These three genes were observed to be repressed in the aerial organs and induced in the latex tissue, which is the site of NR biosynthesis. *TksMYC2* was also found to cluster with *TksAOS2*, *TksAOC1*, and *TksOPR1,* which, based on their expression patterns, may be responsible for a root-specific pathway for JA biosynthesis. *TksJAZ2*, *4,* and *9* in conjunction with *TksLOX2S4* formed a cluster with *TksMYC2* yet exhibited higher expression levels in young lateral roots and lower expression levels in latex. A second cluster of genes included *TksJAR1* and *TksJAZ10* which showed higher expression level in the root, including the latex. This expression pattern suggests that JA conversion in the active form, JA-Ile, by TksJAR1 could take place in the latex. In contrast, JA synthesis genes were downregulated in the latex, suggesting that its synthesis occurs mainly in other root compartments. Other clusters did not show notable modulation between aerial and radical organs. It is noteworthy that the genes in cluster IV displayed increased expression in reproductive organs.

### Cloning and overexpression of TksMYC2 in *Tks* and *Tb*

To determine whether TksMYC2 influences secondary metabolite production, an overexpression vector, containing the complete CDS of *TksMYC2* under the control of the CaMV 35S promoter, was generated and used to stably transform Tks and Tb plants by *Agrobacterium tumefaciens*.

**Fig. 4:**
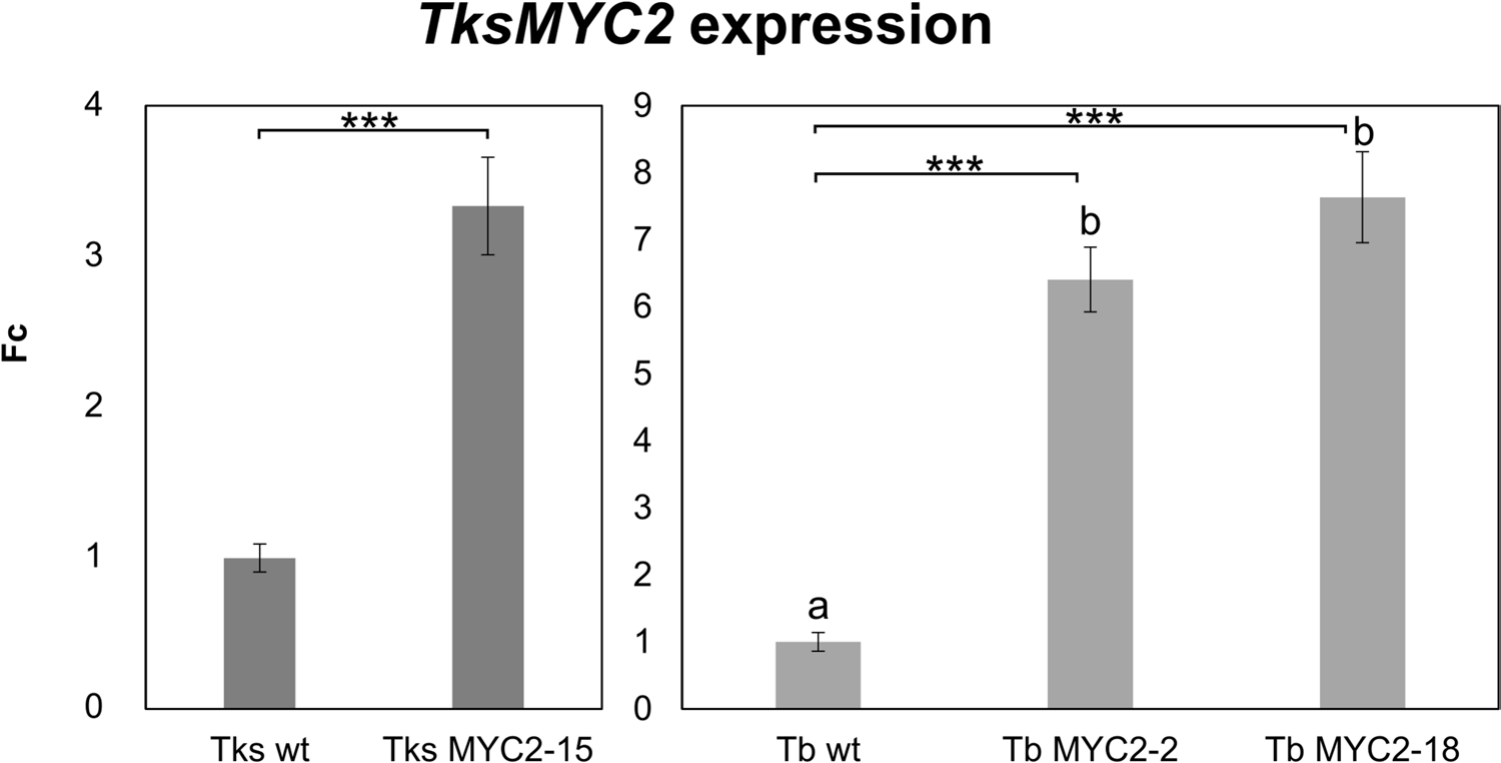
Quantification of *TksMYC2* transcript in wild-type and transgenic Tks and Tb roots. The abundance of the *TksMYC2* transcript was normalized to the abundance of the *GAPC2* housekeeping transcript. Data are presented as fold change of relative *TksMYC2* expression in each genotype with respect to its wild-type. All data are presented as mean ± standard deviation of 3 independent measurements on pools of at least 5 plants/genotype. Asterisks indicate significant differences (***P < 0.001; Student’s t-test with respect to the wild-type). Different letters correspond to significant differences (*P* < 0.05) after ANOVA followed by Tukey’s Honestly Significant Difference post hoc test

Following antibiotic selection, regenerated plants were assayed for *TksMYC2* expression levels. Three independent transgenic *Taraxacum* lines (TksMYC2-15, Tb-MYC2-2, and Tb-MYC2-18) exhibited a significantly increased *MYC2* expression level in comparison to their wild-type, as illustrated in Fig. 4. The TksMYC2-15 line showed 3.3-fold higher *TksMYC2* expression level than the wild-type, while Tb-MYC2-2 and Tb-MYC2-18 lines displayed 6.4-fold and 7.6-fold increases, respectively. Subsequently, the transgenic lines were subjected to secondary metabolite quantification. To produce genetically homogeneous material, Tks lines were propagated by root cutting, while apomictic Tb lines were propagated by seed.

### Metabolite profiling of TksMYC2 overexpressing plants

To evaluate the influence of constitutive TksMYC2 expression on the synthesis of high-value compounds, a metabolite profiling by UHPLC-HRMS and MS/MS was applied to the extracts obtained from leaves and roots of Tks plants. The qualitative/quantitative chemical composition of the TksMYC2 overexpression plants was compared with that of the wild-type plants. All the data recorded in UHPLC-TOF/MS analyses are listed in Table S2, as well as all the MS/MS spectra, which are reported in Figs. S4–S12.

**Fig. 5:**
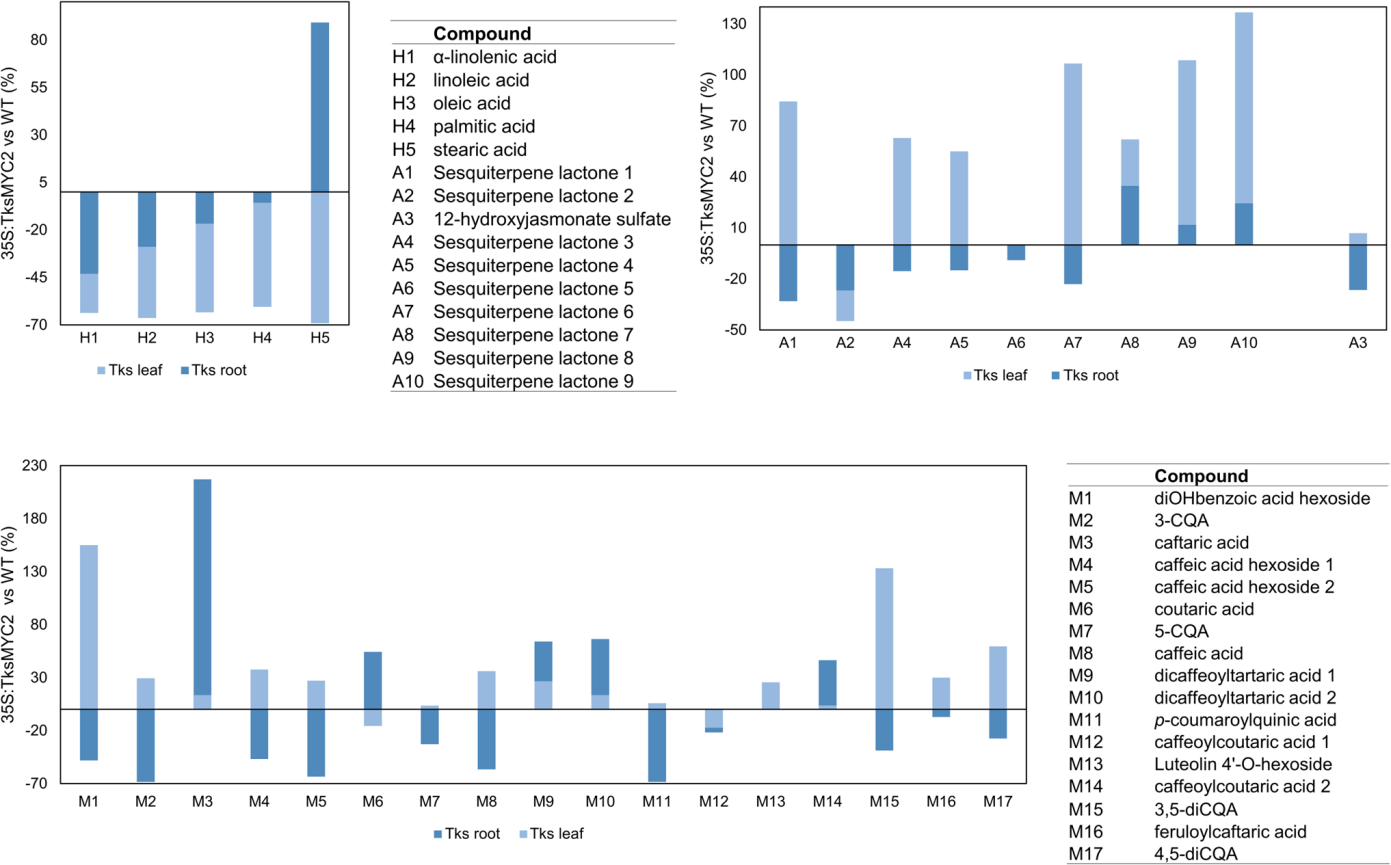
Variation (%) in the content of each metabolite, referred to TksMYC2 overexpressing plants *vs.* wild-type. H1–H5: compounds in the hexane extract; A1–A10: compounds in the acetone extract; M1–M17: compounds in the methanolic extract

In this framework, based on the previous findings regarding metabolite composition in Tks acetone and MeOH extracts (Piccolella et al. 2023), a targeted approach was chosen, focusing on quantitative variations in the content of SLs and (poly)phenols, respectively. Instead, *n*-hexane samples were analysed both qualitatively and quantitatively for the first time. The latter were constituted by free fatty acids, FAs (H1–H5; Table S2), whose total content showed a 2.7- and 1.4-fold decrease in leaf and root samples (Fig. S4), respectively. In particular, this effect was almost comparable for all the compounds in the leaves, with values in the range of −60 to −69%. Instead, it was more pronounced for unsaturated FAs in the roots and was progressively reduced with the decrease in unsaturation degree, until a complete inversion was observed for stearic acid (H5), whose content increased by about 89% (Fig. 5).

Nine SLs (A1, A2, A4–A10; Table S2) were the main constituents of acetone extracts from the roots of Tks wild-type and TksMYC2 overexpressing plants. SLs mainly occurred as glycosides, except for A9, putatively identified as dihydrotaraxinic acid (Fig. S5). Their presence in the leaf extracts was negligible, being 51- and 29-fold lower than the root samples. Furthermore, comparing the root extracts from Tks wild-type and TksMYC2 overexpressing plants, the SL total content was comparable (Fig. S6). In Fig. 5, the content decrease for each metabolite, expressed as %, clearly highlights the compound-specific differences, with values ranging from −33% (A1) to −9% (A6). On the contrary, positive variations were observed for compounds A8–A10, equal to about 35, 12, and 24%, respectively. Although the total accumulation in the leaf extracts was very low, with A6 completely absent, the general variation trend was extremely positive, with an increase in TksMYC2 overexpressing plants roots up to 137% (A10). A2, putatively identified as cynaroside A (or its isomer), was the only SL showing a reduced amount of about 45%.

The only metabolite in the acetone samples not belonging to SLs was A3. Based on its molecular formula (C_12_H_18_O_7_S) and taking into account fragment ions in its MS/MS spectrum, it was characterized as 12-hydroxyjasmonate sulphate (Piccolella et al. 2023). This compound was also peculiarly accumulated in the roots (Fig. S6), and its concentration was 26% lower in TksMYC2 overexpressing plants than in the wild-type (Fig. 5).

With regard to the methanolic extracts, apart from dihydroxybenzoic acid (M1) and luteolin 4′-*O*-hexoside (M13) (Fig. S7), they were constituted by simple phenols and their hexosides (Fig. S8), and mostly their depsides with tartaric and quinic acids (Figs. S9, S10). Considered as a whole, their content slightly increased in TksMYC2 overexpressing plants *vs*. wild-type plant organs (1.2 and 1.3%) (Fig. S11). However, when the contribution of the metabolites was individually analysed, again several differences could be highlighted. In particular, it was observed that the content of caffeic acid (M8), its glycosylated derivatives (M4, M5), and the so-called chlorogenic acids (quinic acid esters; M7, M11, M15, M17) were the most affected in the roots of TksMYC2 overexpressing plants, whereas in the leaves the trend was the opposite. Finally, the depsides containing tartaric acid with very few exclusions showed positive trends both in the leaves and in the roots (Fig. 5).

### Rubber and inulin production in TksMYC2 overexpressing plants

**Fig. 6:**
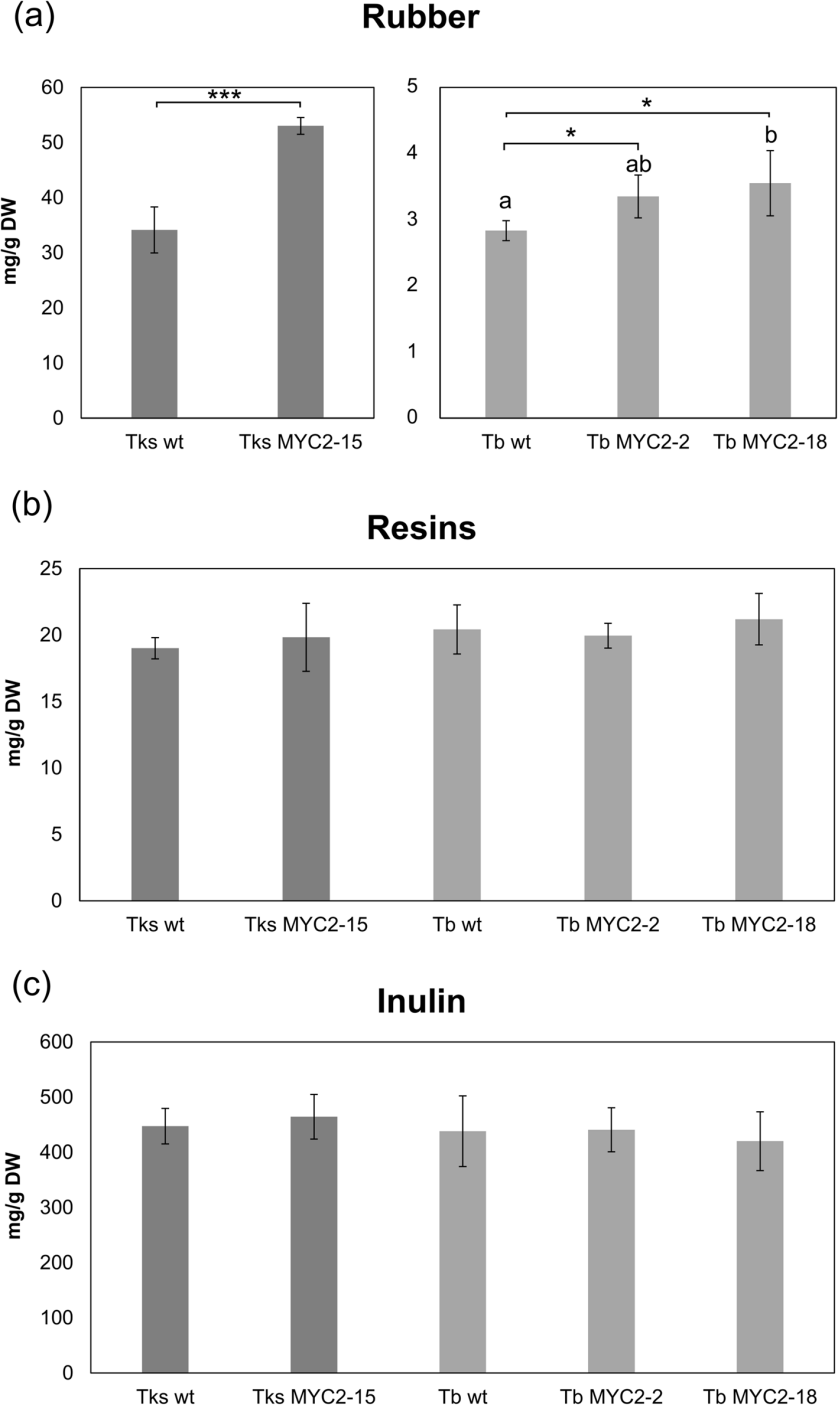
Quantification of rubber, resins, and inulin content in wild-type and transgenic Tks and Tb roots. Content of rubber (**a**), resins (**b**), and inulin (**c**) is presented as mg/g dry weight (DW). All data are presented as mean ± standard deviation of 3 independent measurements on pools of at least 5 plants/genotype. Asterisks indicate significant differences (*P < 0.05, ***P < 0.001; Student’s t-test with respect to the wild-type). Different letters correspond to significant differences (P < 0.05) after ANOVA followed by Tukey’s Honestly Significant Difference post hoc test

A rapid screening method for rubber quantification was used to assess the impact of the constitutive TksMYC2 overexpression on rubber production in Tb and Tks root samples. Afterwards, sequential solvent extraction in an ASE 200 extractor was conducted to simultaneously quantify NR, inulin and resins. As illustrated in Fig. 6a, the NR content, expressed as hexane extractables, was markedly higher in Tks than in Tb wild-type roots, aligning with previous observations (Stolze et al. 2016; Piccolella et al. 2023).

It is noteworthy that the NR content in the roots of the TksMYC2-15 line (53.0 ± 1.5 mg/g DW) exhibited an increase of over 55% in NR compared to the Tks wild-type plants (34.1 ± 4.1 mg/g DW). A significant rise in rubber, 18% and 25%, respectively, was also observed in TbMYC2-2 and Tb-MYC2-18 in comparison to wild-type plants (Fig. 6a).

Conversely, no alterations in resin and inulin content were observed in the Tks and Tb transgenic lines, as illustrated in Fig. 6b, c. Furthermore, the findings substantiated that the inulin and resin content is analogous between the two *Taraxacum* species, as previously observed (Piccolella et al. 2023).

### MYC2 regulates rubber and SLs synthesis genes

**Fig. 7:**
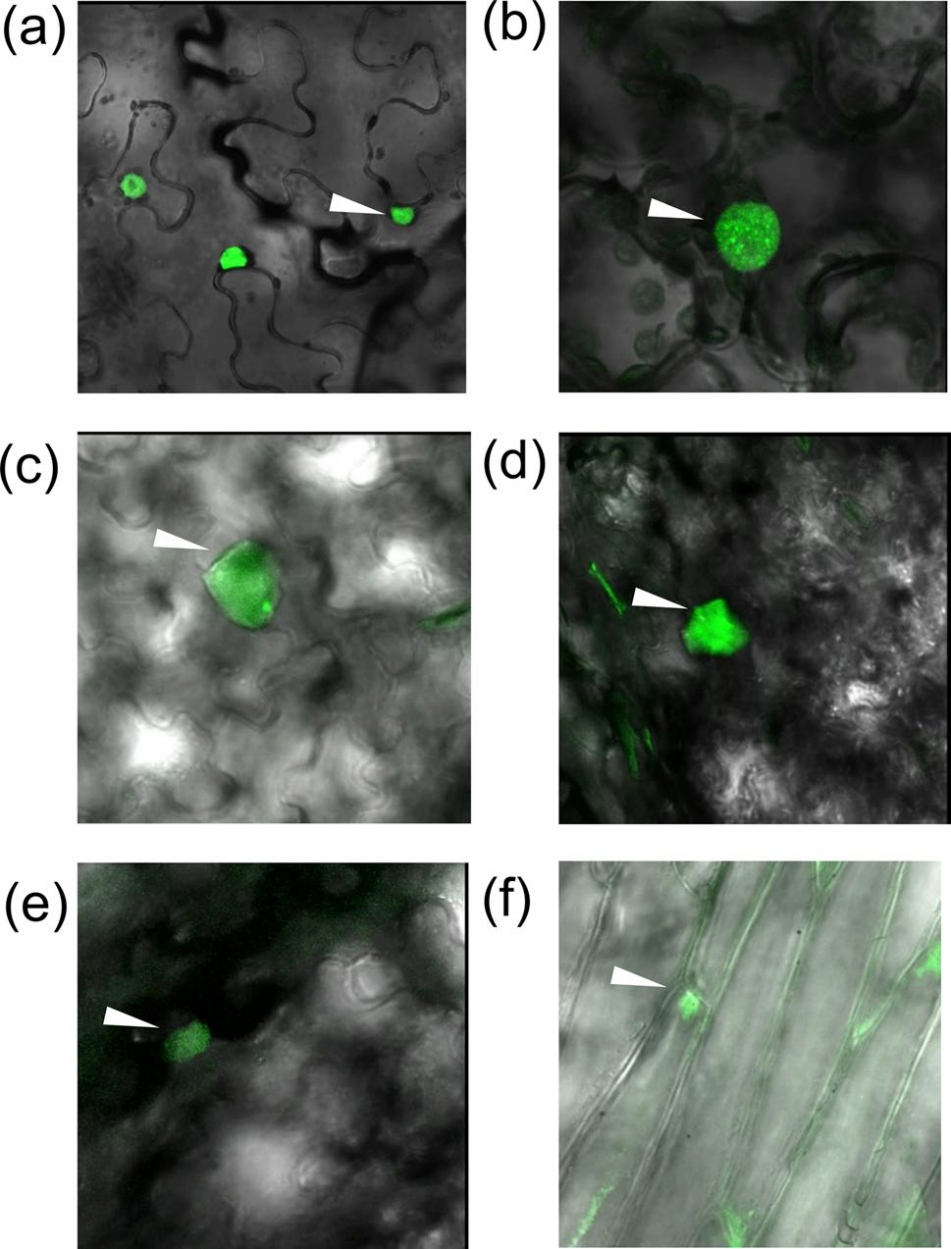
Nuclear localization of the TksMYC2 protein in transient and stable lines. **a**, **b** Images of nuclei in *Nicotiana benthamiana* epidermal leaf cells after transient expression of TksMYC2-GFP. **c**, **d** Images of nuclei expressing GFP in epidermal leaf cells of stable MYC2-GFP lines in *Taraxacum kok-saghyz*. **e**, **f** Images of nuclei expressing GFP in epidermal leaf cells of stable MYC2-GFP lines in *Taraxacum brevicorniculatum*. White arrowhead indicates Taraxacum nuclei expressing GFP. Scale bar is 10 µm

To identify the direct targets of TksMYC2 and to test its ability to bind promoter regions of genes involved in the NR and SLs synthesis pathways, Tks and Tb plants expressing *TksMYC2* tagged to *eGFP* under the control of the CaMV 35S promoter were generated. As expected, the GFP signal was localized in the nucleus of plants expressing the recombinant protein (Fig. 7).

Three-week-old transgenic Tks and Tb plants expressing *TksMYC2:eGFP* were used to perform ChIP using an anti-GFP polyclonal antibody. Wild-type plants of both genotypes were used as negative controls. Given that MYC2 in At is known to bind DNA regions containing G-box motifs (CACGTG) (Song et al. 2022), ChIP-qPCR was performed on the promoter regions enriched in G-box of the Tks genes *CPT2*, *SRPP1*, *SRPP3*, *SRPP4,* and *GAO*. CPT2 and SRPPs genes were selected based on their role in NR biosynthesis. Germacrene A Oxidase (GAO) was selected for its role in the biosynthesis of SLs, compounds that are abundant in both Tks and Tb roots (Piccolella et al. 2023). GAO is a cytochrome P450 that catalyses the three-step sequential oxidation of germacrene A to Germacrene A acid (GAA), which is a precursor of germacranolides, a class of SLs (Nguyen et al. 2010).

**Fig. 8:**
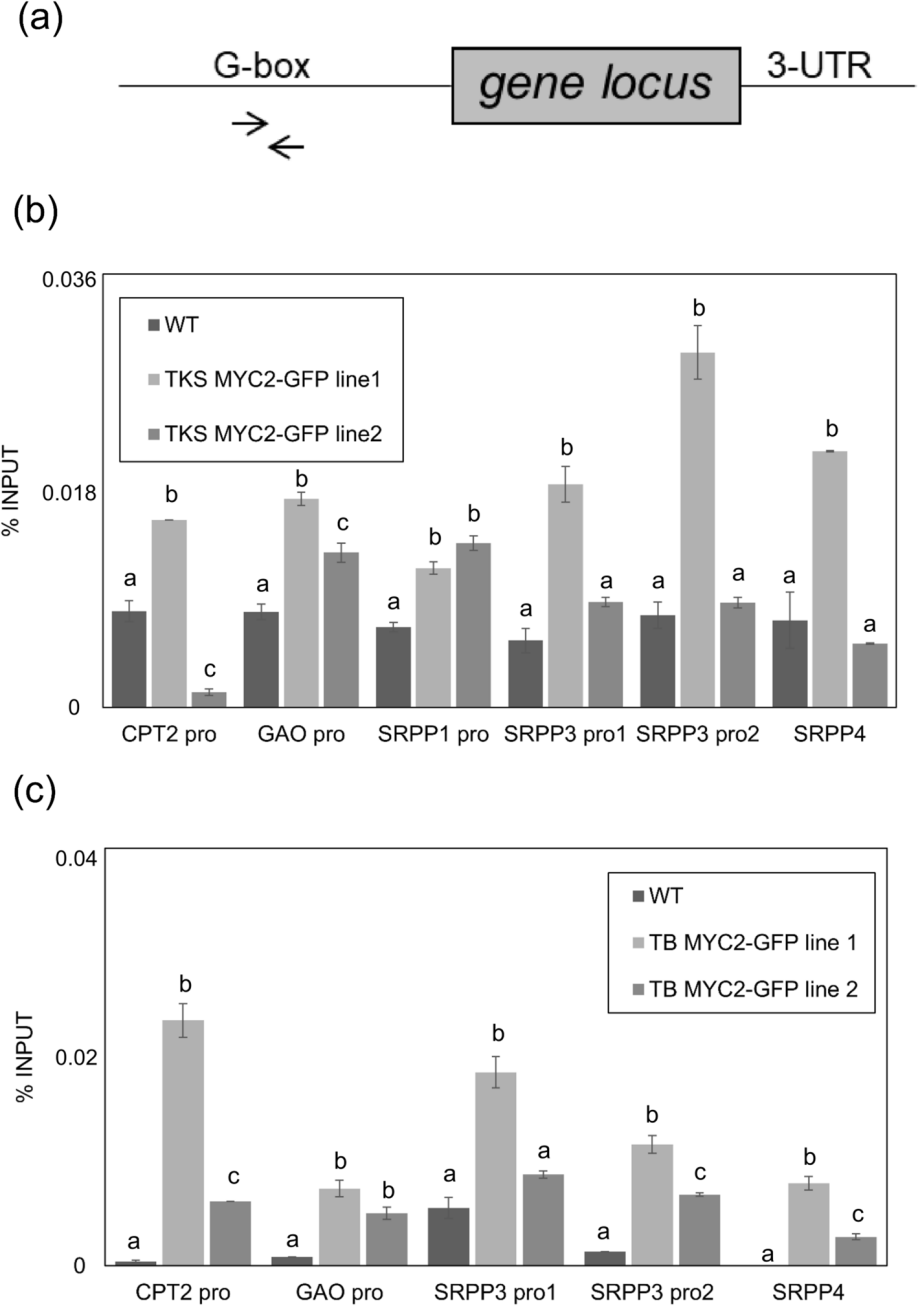
Taraxacum MYC2 interaction with rubber and other metabolite synthesis genes. **a** Schematic representation of gene locus with ChIP primer pairs amplifying promoter regions containing G-box motifs (CACGTG). **b** Relative enrichment of MYC2 in the promoter regions of CPT2, GAO, SRPP1, SRPP3, and SRPP4 loci in 35S:MYC2-GFP lines 1 and 2 in *Taraxacum kok-saghyz*, **c** Relative enrichment of MYC2 in the promoter regions of CPT2, GAO, SRPP3, and SRPP4 loci in 35S:MYC2-GFP lines 1 and 2 in *Taraxacum brevicorniculatum*. Wild-type plants were used as negative control for chromatin immunoprecipitation using anti-GFP antibody. Different letters correspond to significant differences (*P* < 0.05) after ANOVA followed by Tukey’s Honestly Significant Difference post hoc test

Additionally, the selected genes showed high expression level in high NR compared to low NR producing Tks plants (Panara et al. 2018). All the selected genes have a homolog in Tb with the exception of TksSRPP1 (Panara et al. 2018). The *SRPP3* locus was found to contain two distinct G-boxes. Amplifications spanning the different regions revealed that in both Tks and Tb, TksMYC2 can associate with the promoter regions of the aforementioned targets (Fig. 8). In Tks this association was more evident in line 1 than line 2, compared to the wild-type, particularly for *SRPP3* and *SRPP4*. This was likely due to differences in protein levels. However, in Tb, both GFP lines showed a consistent binding across all the analysed regions. Taken together, these results suggest that TksMYC2 can promote the expression of rubber synthesis genes by associating with their promoter elements, thereby confirming its function as transcription factor also in perennial plants.

### Effect of TksMYC2 overexpression on rubber and SLs related genes

In order to determine whether TksMYC2 overexpression affects *CPT2*, *SRPP1, SRPP3, SRPP4,* and *GAO,* their mRNA levels were analysed in both Tks and Tb MYC2 overexpressing lines. Samples were collected from wild-type and transgenic lines and subjected to qRT-PCR using target-specific primers and glyceraldehyde-3-phosphate dehydrogenase C2 (*GAPC2*) as an internal control.

**Fig. 9:**
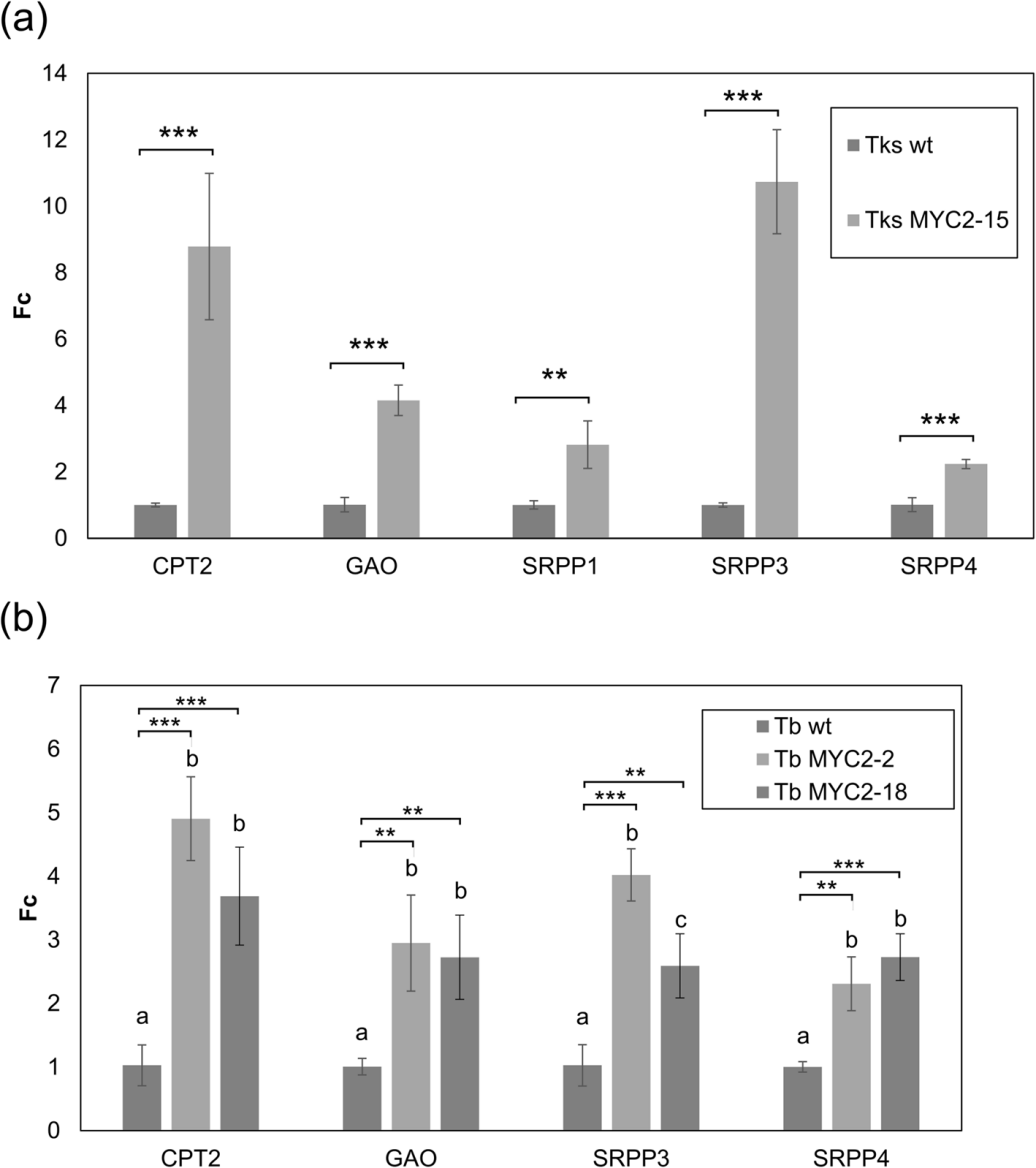
Expression analysis of rubber and SLs related genes in wild-type and transgenic Tks (**a**) and Tb lines (**b**). The abundance of the transcripts was normalized to the expression of the *GAPC2* housekeeping transcript. Data are presented as fold change of relative genes expression in each genotype with respect to its wild-type. All data are presented as mean ± standard deviation of 3 independent measurements on pools of at least 5 plants/genotype. Asterisks indicate significant differences (*P < 0.05, ***P < 0.001; Student’s t-test with respect to the wild-type). Different letters correspond to significant differences (*P* < 0.05) after ANOVA followed by Tukey’s Honestly Significant Difference post hoc test

Expression analysis demonstrated that all the genes were significantly upregulated in the Tks and Tb transgenic lines compared to the wild-type plants, as illustrated in Fig. 9. In particular, the gene encoding the key enzyme in rubber production, *CPT2*, was markedly overexpressed in all transgenic lines in both Tks and Tb, with increases ranging from 3.7-fold to more than eightfold.

Another gene that exhibited a strong upregulation in TksMYC2 overexpressing lines was *SRPP3*, which showed a more than tenfold increase. This gene also displayed a notable overexpression in the two Tb transgenic lines, with 2.5- and fourfold increases compared to wild-type plants.

*SRPP1* and *SRPP4* also showed a more than twofold increase in their transcripts in Tks MYC2 overexpressing lines in comparison to the wild-type plants. An increase in the transcript level of *SRPP4* (more than twofold) was also observed in Tb MYC2 overexpressing lines.

Finally, it was observed that *GAO* transcripts were present in greater quantities in transgenic lines of Tks and Tb with respect to wild-type plants, with increases ranging from 2.8- to over fourfold.

The results demonstrate that the overexpression of the TksMYC2 transcription factor consistently stimulates the expression of several pivotal genes involved in the rubber and SLs biosynthetic pathways in both Tks and Tb plants, indicating an important role for this gene in the production of valuable compounds in *Taraxacum* species.

## Discussion

MYC2 is a master transcription factor considered the main downstream effector of JA signalling pathway (Luo et al. 2023). Several studies have highlighted its important role in the regulation of numerous physiological and biochemical processes including plant growth and development, responses to biotic and abiotic stresses, and the biosynthesis of specialized metabolites.

Indeed, NtMYC2a/b activate the expression of nicotine biosynthesis genes in *N. tabacum* (De Boer et al. 2011; Zhang et al. 2012), while AaMYC2 activates the expression of genes encoding artemisinin biosynthetic enzymes in *Artemisia annua* (Shen et al. 2016); TcMYC2a positively regulates the biosynthesis of taxol in *Taxus chinensis* (Zhang et al. 2018); SmMYC2a/b control tanshinone and phenolic acids biosynthesis in *Salvia miltiorrhiza* (Zhou et al. 2016).

In this work we identified the homolog of AtMYC2 in Tks. Similarity, phylogenetic and collinearity analyses between Tks and At showed that evm.model.utg1303.12 protein (TksMYC2) has a high degree of conservation with AtMYC2 (Fig. 2a,b). TksMYC2 possesses the main functional domains found in AtMYC2 and in most known plant MYC2 s: the bHLH-leucine zipper, typical of the bHLH superfamily, and the bHLH-MYC-N, as found in the MYC subfamily (Fig. 2d).

The expression analysis of *TksMYC2* revealed that it is predominantly expressed in hypogeal organs, including the latex tissue, in comparison to aerial parts (Fig. 3a). Furthermore, previous research has demonstrated that a significant discrepancy is present in the metabolite composition of acetone extracts derived from the leaves and roots of Tks (Piccolella et al. 2023), and that the root is the organ where NR production is more abundant (van Beilen and Poirier 2007). This suggests that MYC2 may regulate genes associated with root metabolism.

The JA signalling pathway appears to be required in the biosynthesis of specialized metabolites such as isoprenoids (Afrin et al. 2015). This aspect has been demonstrated in several plant species, including *Arabidopsis thaliana*, *Medicago truncatula*, *Nicotiana tabacum*, and *Catharanthus roseus*, where treatment with MeJA has been shown to induce the expression of genes involved in pathways leading to different classes of specialized metabolites as reviewed by Pauwels and co-authors (Pauwels et al. 2009). In Hb, MeJA was observed to stimulate NR production and the transcription of its related genes (Deng et al. 2018). Similarly, in Tks, MeJA treatment was able to induce the transcription of genes involved in the MVA and rubber biosynthesis pathways (Cao et al. 2017; Dong et al. 2023b). The majority of genes involved in JA synthesis and signalling were found to be upregulated by MeJA treatment in Tks (Dong et al. 2023b). Our findings demonstrate that most of these genes exhibit an expression pattern among plant organs similar to that of TksMYC2 (Fig. 3b). Notably, JAZ1 and JAZ7, which showed high expression in the latex, may serve as JA receptors in this tissue, where they could act in the JA-mediated regulation of genes such as those involved in NR synthesis.

To better elucidate the role of TksMYC2 in the regulation of metabolite biosynthesis in *Taraxacum*, including NR, we generated overexpression lines, both in Tks and Tb, harbouring a 35S:*TksMYC2* construct. Analysis of root extracts from TksMYC2 overexpressing lines revealed a significant increase in NR production in Tks and, to a lower extent, in Tb, while no alteration of inulin and resins content has been observed (Fig. 6b, c). Our findings are in accordance with those presented by Wu et al. in a recent study, wherein it was demonstrated that MYC2 overexpression resulted in a significant increase in Tks NR production (Wu et al. 2024). Nevertheless, to the best of our knowledge, this is the first reported case of genetic engineering in Tks that led to a NR content increase exceeding 50% (Fig. 6a).

The metabolite analysis of n-hexane, acetone, and methanolic extracts from leaves and roots revealed differences between the wild-type and TksMYC2 overexpressing Tks plants (Fig. 5).

The main free FAs of Tks identified in our analysis are in accordance with those previously reported (Ramirez-Cadavid et al. 2017). In general, TksMYC2 overexpression resulted in a significant decrease of FAs in both leaves and roots, which represent an important carbon reserve in plants (He and Ding 2020). Such a reduction may be caused by a redirection of the carbon source towards rubber accumulation. Indeed, terpenoids and FAs share common precursors from glycolysis and the pentose phosphate pathway (Niu et al. 2015). On the contrary, the 89% increase in stearic acid observed in roots was probably caused by a decrease in SAD (stearoyl-ACP desaturase) activity. SAD is the main enzyme involved in the conversion of stearoyl-ACP to the unsaturated H1, H2, and H3 compounds (He and Ding 2020). Its activity is also linked to defence signalling mechanisms in which MYC2 is involved. Indeed, loss of function of a SAD isoform in tomato resulted in a reduction in JA levels and the disruption of JA-mediated signalling pathways (Quevedo-Colmena et al. 2024).

NR and SLs are the main compounds accumulated in the Asteraceae plants latex (Frey et al. 2024). It has been previously demonstrated that MYC2 induces the expression of genes involved in the biosynthesis of the SL artemisinin in *A. annua* (Shen et al. 2016; Frey et al. 2024). We observed that the majority of identified SLs increased in the leaves of TksMYC2 overexpressing lines, thereby providing support for the hypothesis that TksMYC2 may activate their biosynthesis (Fig. 5). In Tks roots we did not observe relevant differences in total SLs. Thus, in this organ, the effect of TksMYC2 constitutive expression appears to be more specific, namely, in the activation of NR production rather than SLs. The finding that SLs are not affected by the subtraction of their precursors by NR biosynthesis was previously observed in lettuce. Indeed, in Ls the competition for IPP between the two pathways is avoided by recruiting IPP from parenchyma cells in close proximity to laticifers for the synthesis of SL backbones (Kwon et al. 2022). An examination of individual SLs in the roots revealed a decrease in several compounds (A1-A7) and an increase in others (A8-A10) in the TksMYC2 overexpressing roots (Fig. 5). This result suggests that TksMYC2 may have a role in finely regulating the biochemical pathways, which remain largely unexplored, that lead to the synthesis of the different classes of SLs from the common costunolide precursor.

Interestingly, the jasmonate derivative A3 (12-hydroxyjasmonate sulphate) increased in the leaves and decreased in the roots of TksMYC2 overexpressing plants (Fig. 5). Although this compound showed no biological activity in potato, it may have an effect on JA-mediated regulation of gene expression (Miersch et al. 2008). This modulation suggests a possible interaction with MYC2 signalling in Tks.

Furthermore, TksMYC2 overexpression resulted in a slight increase in phenylpropanoid content both in leaves and roots (Fig. 5). In At, the activation of phenylpropanoid metabolism by MeJA treatment was observed both at transcriptional and metabolic levels (Pauwels et al. 2009). Additionally, it was observed that genes involved in phenylpropanoid/flavonoid biosynthesis lost their MeJA responsiveness in the At myc2 mutant (Dombrecht et al. 2007). Thus, we speculate that a positive relationship between phenylpropanoids, MYC2, and JA may also occur in Tks. All the compounds observed in leaf methanolic extracts increased with the exception of coutaric acid (M6) and its derivative caffeoyl-coutaric acid (M12), suggesting that this branch of phenylpropanoid biosynthesis could be differently regulated in Tks. In contrast, in the roots, all the observed compounds decreased with the exception of chicoric acid (M9, M10) and its precursor caftaric acid (M3), as well as of coutaric acid (M6), and caffeoyl-coutaric acid (M14). All of these compounds have in common the presence of a tartaric acid moiety. This evidence may be explained by a differential control of hydroxycinnamoyl-CoA:tartaric acid hydroxycinnamoyltransferase (HTT), the enzyme that catalyses the addition of tartaric acid, with respect to hydroxycinnamoyl-CoA:quinate hydroxycinnamoyltransferase (HQT). In fact, in *T. antungense*, a positive correlation between HQT expression and 5-caffeoylquinic acid concentration was observed in TaHQT1 and TaHQT2 RNAi lines (Liu et al. 2019). Furthermore, in transgenic hairy roots of *Echinacea purpurea*, EpHTT and EpHQT overexpression or silencing corresponded to increased and decreased caftaric acid and chlorogenic acid levels, respectively (Fu et al. 2021).

In plants, MYC2 interacts with specific regions in the promoter of controlled genes, such as the G-BOX element (Song et al. 2022). To investigate TksMYC2 potential interaction with candidate genes, we looked for the presence of the G-BOX motif in their 5’ region using available Tks genomic data (Lin et al. 2017). A single G-BOX motif was identified for TksCPT2, TksSRPP1, TksSRPP4, and TksGAO. TksSRPP3 showed two distinct G-BOXES. It was previously reported that *GUS* gene expression driven by the *TksSRPP3* promoter was upregulated by MeJA treatment in Tobacco (Dong et al. 2023a).

The binding of TksMYC2 with the G-BOX region of *TksCPT2*, *TksSRPP1*, *3,* and *4*, and *TksGAO* was demonstrated by ChIP-PCR assay on *Taraxacum* plants expressing TksMYC2:eGFP (Figs. 7,8). The expression of these genes in TksMYC2 overexpressing plants showed a significant increase compared to the wild-type (Fig. 9). Taken together, these data suggest that MYC2 regulates the expression of several genes involved in NR and SLs biosynthesis by directly interacting with their promoter, in both Tks and Tb.

The possible mechanisms of action of MYC2 in the regulation of metabolite biosynthesis were previously described (Song et al. 2022; Luo et al. 2023). In Hb, it has been proposed that a module composed of COI1-JAZ3-MYC2 could induce the expression of HbFPS1 and HbSRPP1 which are NR biosynthesis-related genes (Deng et al. 2018). In our study, two JAZ genes were identified as being co-expressed with MYC2: JAZ1 and JAZ7. It is plausible hypothesize that these two genes may constitute a part of the aforementioned module in Tks. Concerning COI1, five similar proteins have been identified in the Tks genome, all of which are expressed in the latex (Lin et al. 2017). The five putative COI1 genes were not modulated by MeJA treatment (Dong et al. 2023b). Further studies are required to identify a possible COI1 candidate for the interaction with TksMYC2.

## Conclusions

This study demonstrates how increased MYC2 transcription factor expression can induce significant changes in the physiology of two dandelion species (*Taraxacum kok-saghyz*, Tks and *Taraxacum brevicorniculatum*, Tb) of great interest to the agro-industry as potential sources of high-value-specialized metabolites and natural rubber. Here, we demonstrated that the overexpression of TksMYC2 affects the production of essential specialized metabolites, including SLs, phenylpropanoids, and free FAs. Moreover, our experiments showed that MYC2 overexpression can significantly enhance rubber production both in Tks and Tb species. These findings will lead to renewed efforts to promote the cultivation of the Russian dandelion as a potential alternative source of high-value compounds, particularly NR.

## Supplementary Information

Below is the link to the electronic supplementary material.

Supplementary file1 (PDF 1050 KB)

Supplementary file2 (PDF 476 KB)

## Data Availability

Sequence data of genes analysed in this study are publicly available; all the other data supporting the findings of this study are available from the corresponding authors upon reasonable request.
