# Supplementary material for: MYC2 influences rubber and sesquiterpene lactones synthesis in Taraxacum species
Source: Planta. 2025 May 24;262(1):5. doi: 10.1007/s00425-025-04719-9 (PMC12103366; doi:10.1007/s00425-025-04719-9)
Supplement: Supplementary file 1 — Supplementary file1 (PDF 1050 KB) [file 425_2025_4719_MOESM1_ESM.pdf]

Elio Fantini[1*], Loretta Daddiego[1*], Paolo Facella[*1], Giorgio Perrella[1,2], Linda Bianco[1], Carlo Fasano[1], Fiammetta Alagna[1], Michele Antonio Savoia[1,3], Daniela Rigano[4], Carmina Sirignano[4], Orazio Taglialatela Scafati[4], Severina Pacifico[5], Simona Piccolella[5], Loredana Lopez[1], Francesco Panara[1**]**

***** Equal contribution

****** Corresponding authors

TksMYC2 Interacts with Rubber and Sesquiterpene Lactones Synthesis Genes Promoters and its Overexpression Drives Metabolite Synthesis Changes in Dandelion

[1] Trisaia Research Center, ENEA, S.S. 106 Ionica - Km 419+500, 75026 Rotondella (MT), Italy

[2] Department of Biosciences, University of Milan, Via Celoria 26, 20133, Milan, Italy

[3] Department of Soil, Plant and Food Sciences, University of Bari Aldo Moro, Via Amendola 165/A, 70126 Bari, Italy

[4] Department of Pharmacy, School of Medicine and Surgery, University of Naples Federico II, Via D. Montesano 49, 80131 Naples, Italy

[5] Department of Environmental Biological and Pharmaceutical Sciences and Technologies, University of Campania “Luigi Vanvitelli”, Via Vivaldi 43, I-81100 Caserta, Italy

Francesco Panara

E-mail: francesco.panara@enea.it

Telephone: +39-0835-974523

Orcid-ID: https://orcid.org/0000-0001-9596-7262

Loredana Lopez

E-mail: loredana.lopez@enea.it

Telephone: +39-0835-974355

Orcid-ID: https://orcid.org/0000-0002-7129-9893

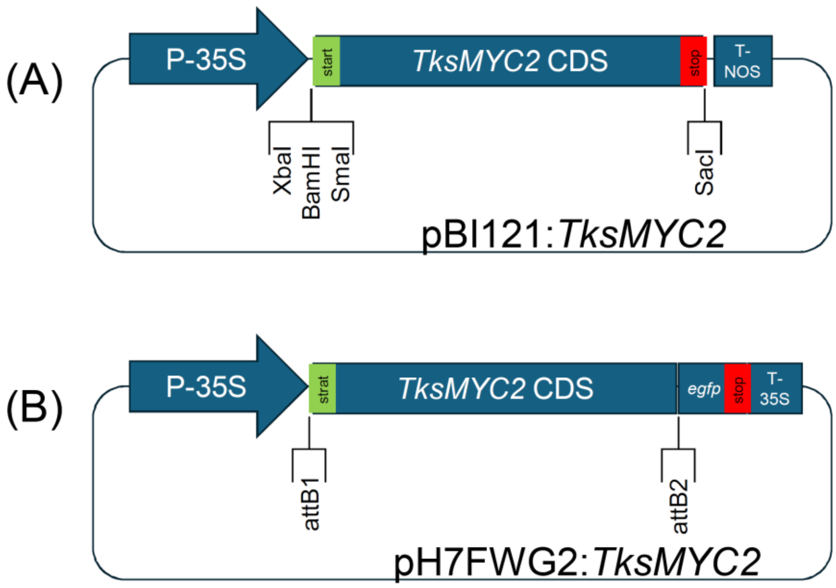

Figure S1 (A) map of pBI121:*TksMYC2*. (B) map of pH7FWG2:*TksMYC2*.

A

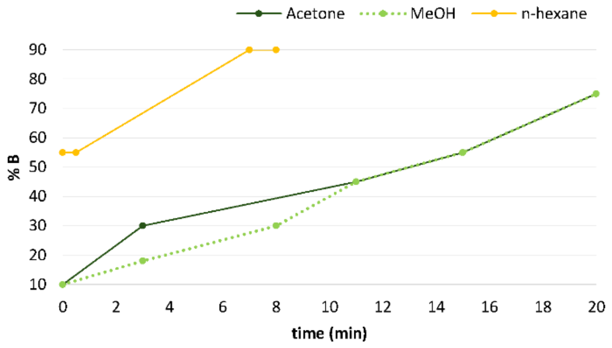

A Water (0.1 % formic acid)

B Acetonitrile (0.1 % formic acid)

B

	n-hexane extracts	acetone extracts	methanol extracts
**Mass range**			
▪ Full scan TOF survey	220-1000	120-1200	120-1200
▪ IDA* scans	150-850	90-900	90-900
**Accumulation time**			
▪ Full scan TOF survey		250 ms	
▪ IDA* scans		100 ms	
**Gases**			
▪ Curtain		35 psi	
▪ Nebulizer		60 psi	
▪ Heated		60 psi	
**Source parameters**			
▪ Ion spray voltage		-4500 kV	
▪ Interface heater temperature	600 °C	500 °C	
**Compound ionization**			
▪ Declustering potential	60	80	
▪ Collision energy	45	35	
▪ Collision energy spread	15	15	

*Information Dependent Acquisition

Figure S2 (A) Elution gradient utilized to analyse methanol, acetone, and n-hexane extracts. (B) Mass spectrometry experimental parameters.

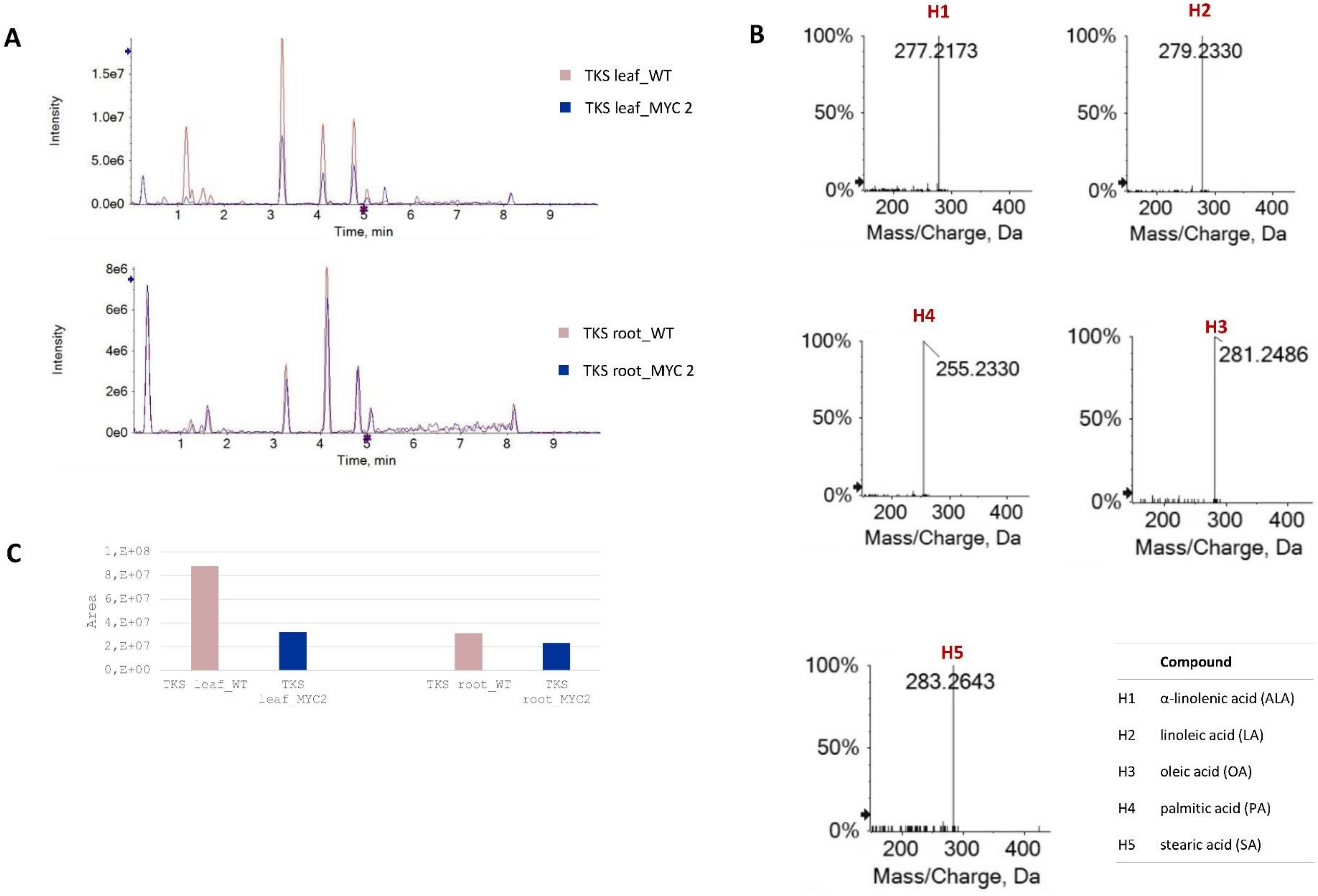

	Compound
H1	α-linolenic acid (ALA)
H2	linoleic acid (LA)
H3	oleic acid (OA)
H4	palmitic acid (PA)
H5	stearic acid (SA)

Figure S4 (A) Total Ion Current (TIC) chromatograms of n-hexane extracts. (B) TOF-MSMS spectra of fatty acids identified therein. (C) Total fatty acid content, based on peak areas.

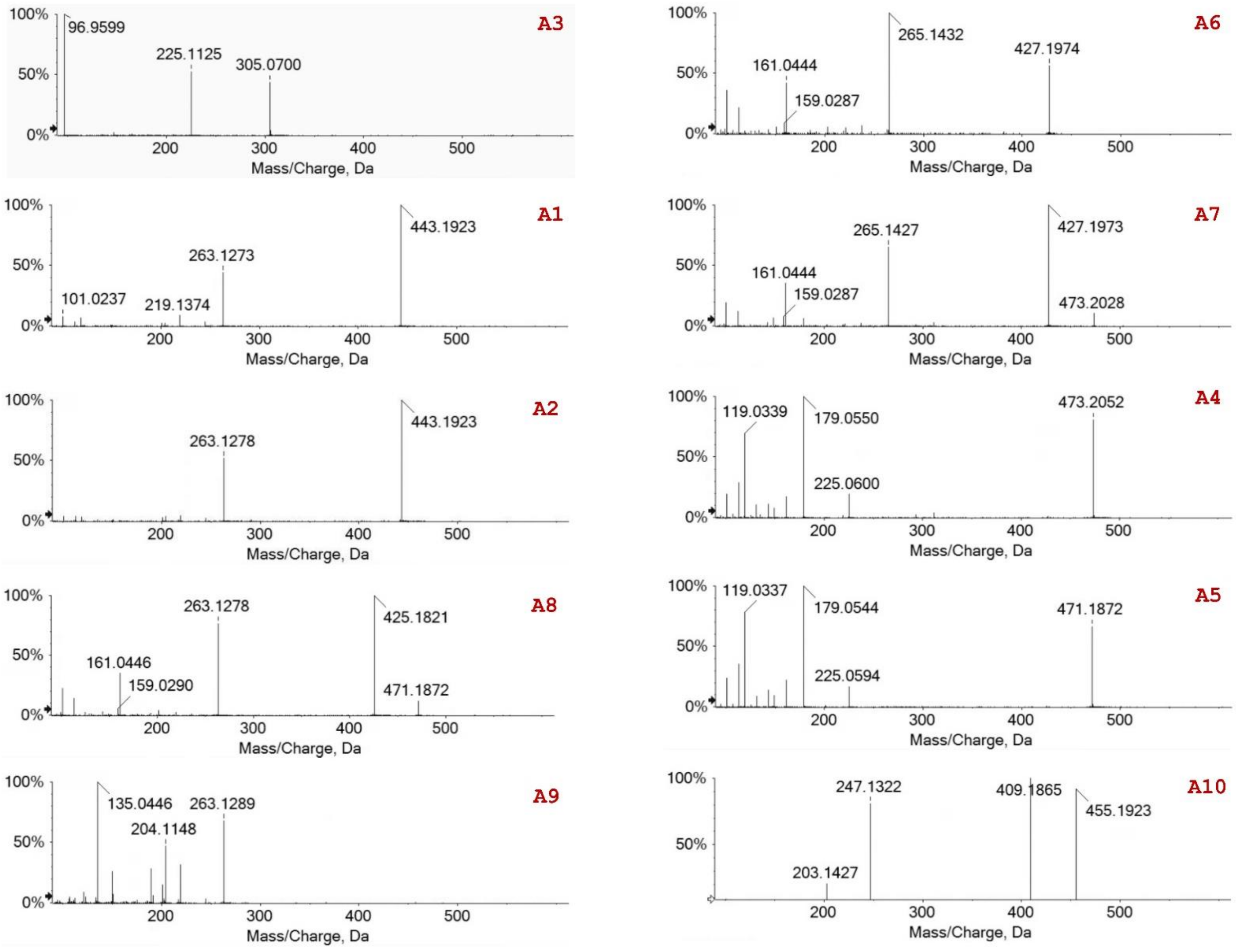

Figure S5 TOF-MSMS spectra of metabolites identified in acetone extracts.

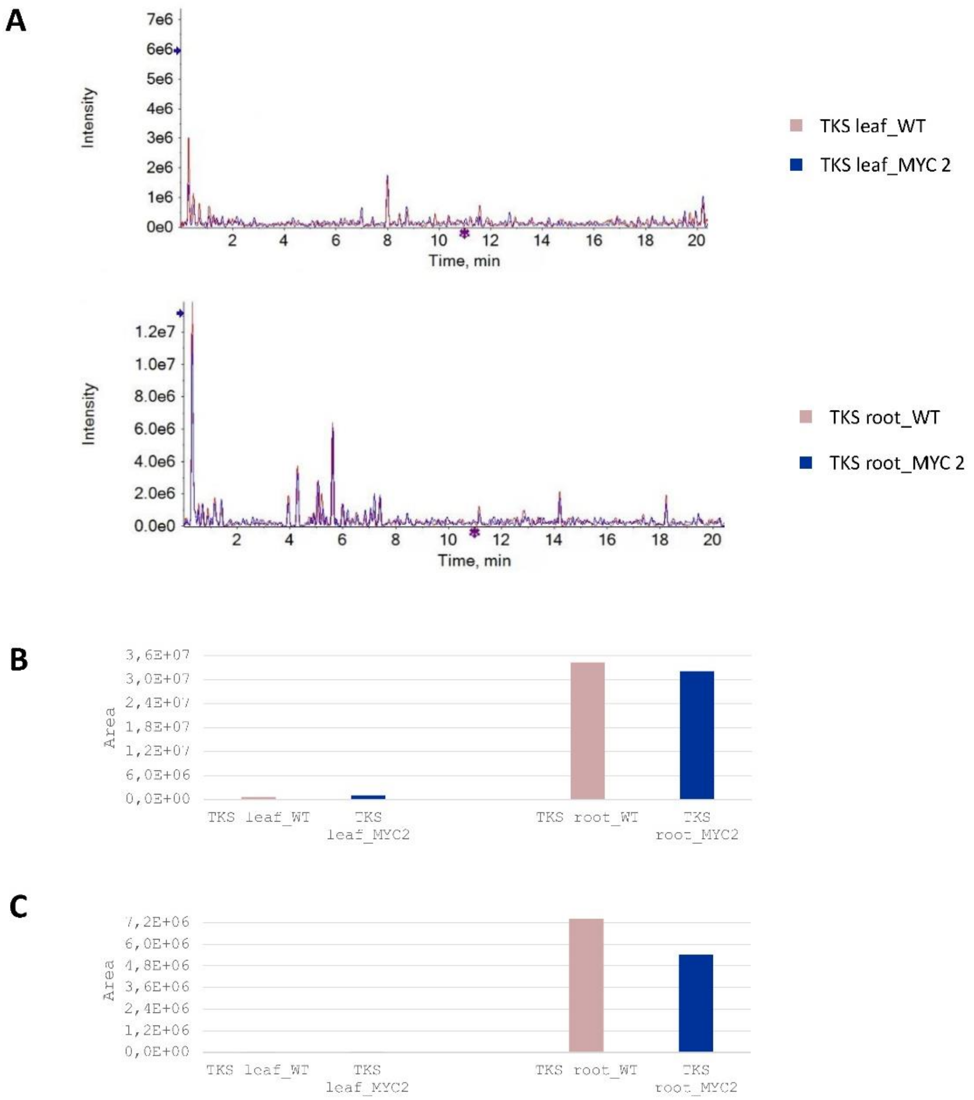

Figure S6 (A) Total Ion Current (TIC) chromatograms of acetone extracts. (B) Total sesquiterpene lactone content, based on peak areas. (C) 12-hydroxyjasmonate sulfate content, based on peak areas.

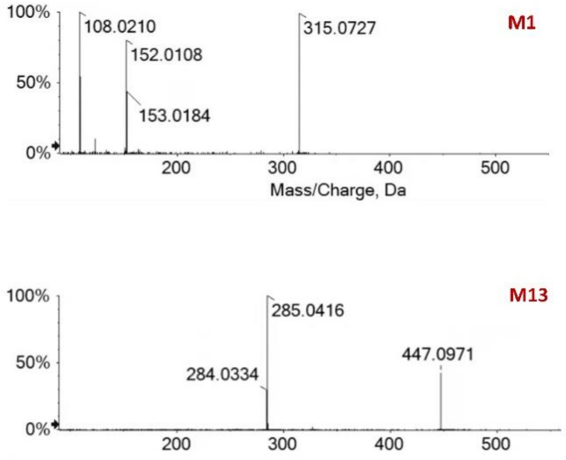

Figure S7 TOF-MSMS spectra of dihydroxybenzoic acid (M1) and luteolin 4'-O-hexoside (M13) from methanolic extracts.

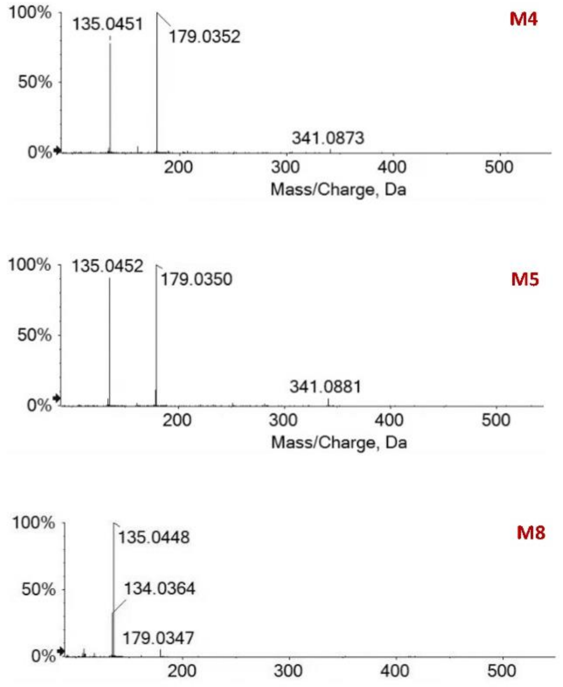

Figure S8 TOF-MSMS spectra of caffeic acid (M8) and its hexosyl derivatives (M4, M5).

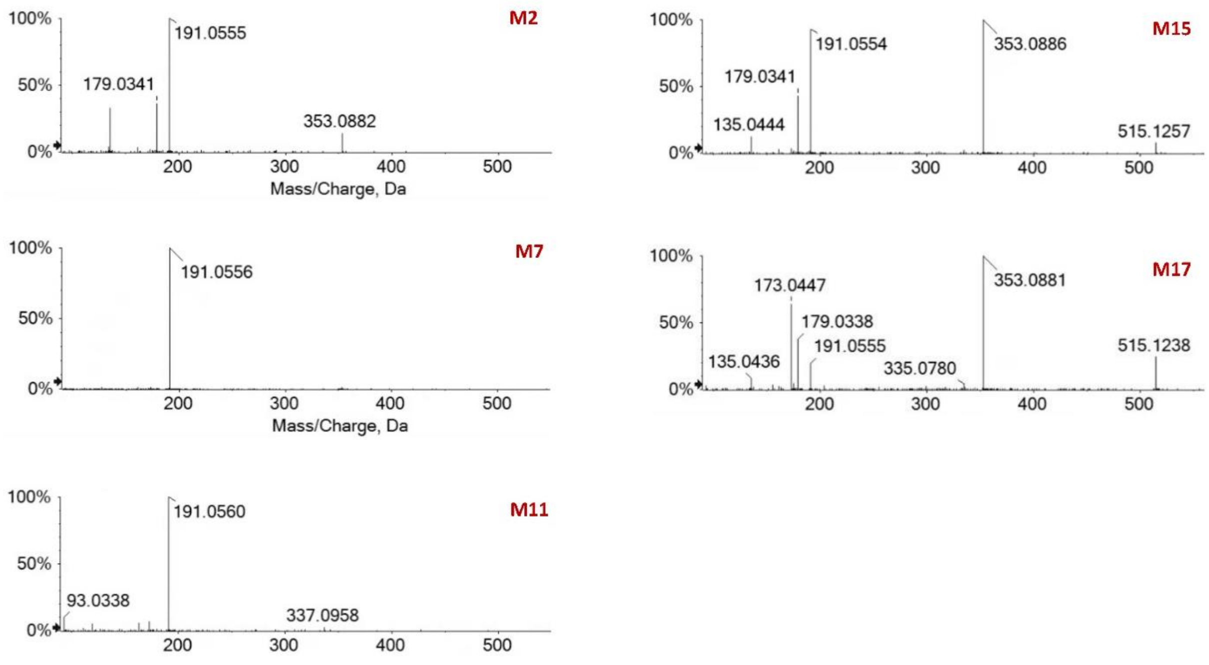

Figure S9 TOF-MSMS spectra of quinic acid depsides identified in the methanolic extracts.

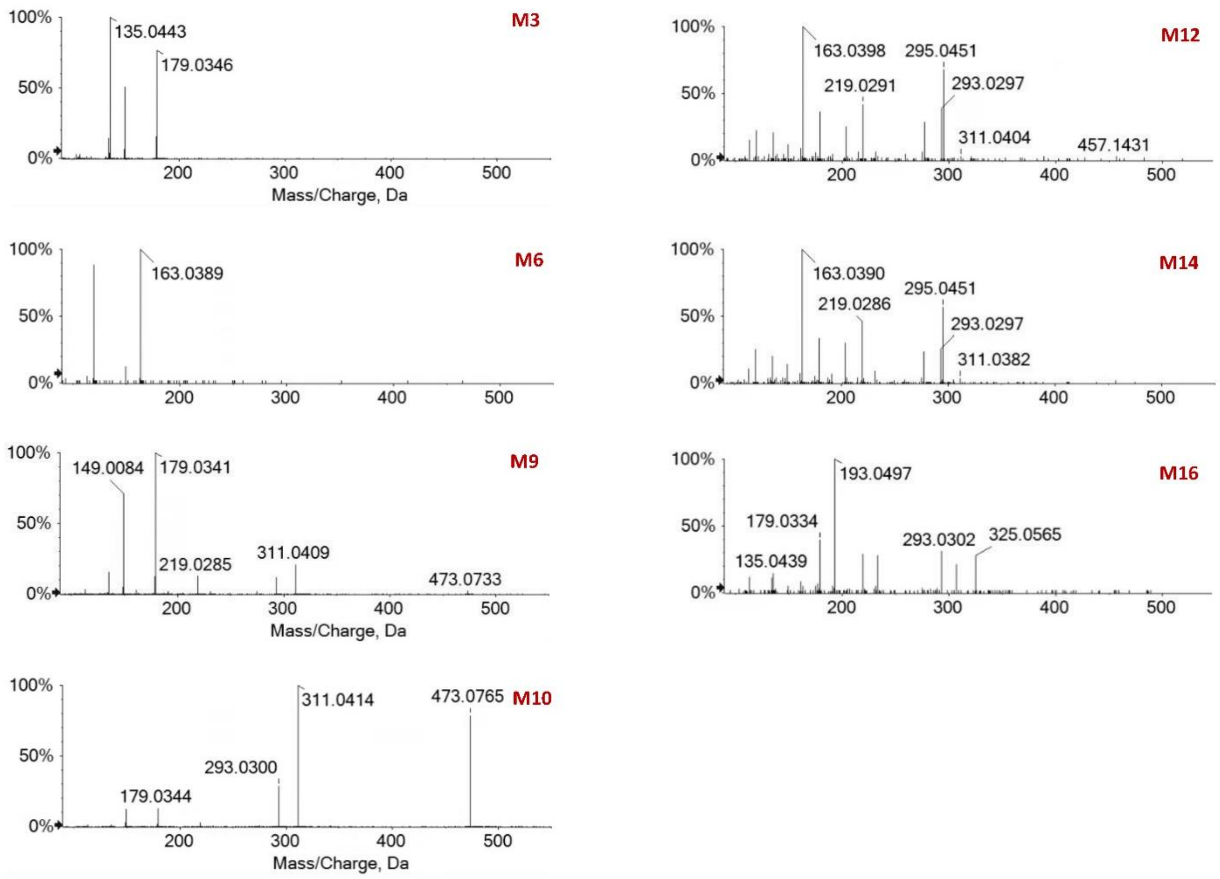

Figure S10 TOF-MSMS spectra of tartaric acid depsides identified in the methanolic extracts.

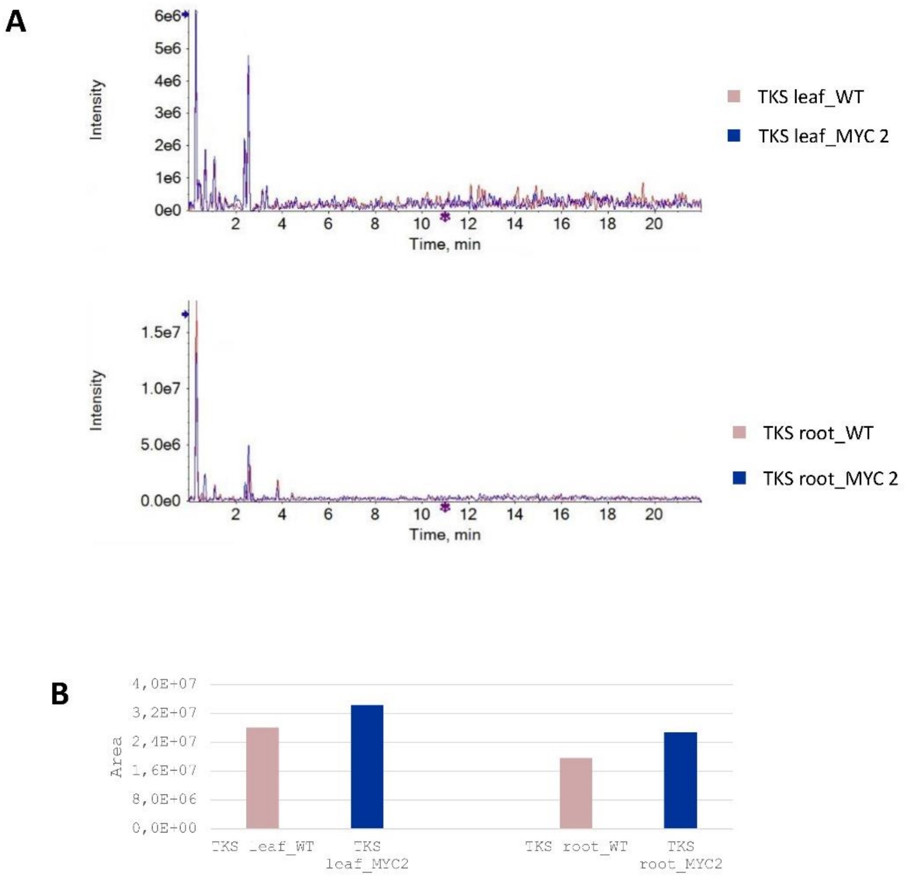

Figure S11 (A) Total Ion Current (TIC) chromatograms of methanol extracts. (B) Total phenol content, based on peak areas.

Table S1 5'-3' sequence of oligonucleotides used in this work.

**qRT-PCR**	
MYC2_for	TCGAAGTCAACCATGCTAGT
MYC2_rev	TCGGAGCTGATCTTGAGTG
GAO_for	CATCCAAGAAAATTATCCACAC
GAO_rev	GTGTTGGGTGATGACATCAG
SRPP3_for	GAGGAAACATGATTGAAATAAGTG
SRPP3_rev	TGTAAACATTACCGCCAGAT
SRPP4_for	CAATCAGTGACCTCCCTCTT
SRPP4_rev	GAAACCTTATACCCTTCGTCTG
CPT2_for	GAAGCTTGTGAAGAAAAGGA
CPT2_rev	CTGACTTCCATGATTCACG
GAPC2_for	GTTTCGTGGTATGACAACGAG
GAPC2_rev	TCACCCGACATCCAACAC
SRPP1_for	GTACAACCAGACTGTGCAGC
SRPP1_rev	TCTTCCATTATCCATCACAAAA
**TksMYC2 cloning into pBI121**	
TksMYC2_BamHI_for	GGTGGTGGATCCATGACGGATTACCGGCTACA
TksMYC2_SacI_rev	GGTGGTGAGCTCTTACAATGGATCTGAAAATCTG
**TksMYC2 cloning into pDONOR221**	
TksMYC2_B1	GGGGACAAGTTTGTACAAAAAAGCAGGCTTAATGACGGATTACCGGCTAC
TksMYC2_B2	GGGGACCACTTTGTACAAGAAAGCTGGGTCCAATGGATCTGAAAATCTG
**Identification of positive Tks and Tb plants transformed with pBI121:TksMYC2**	
TkMYC2_int_for2	AAGTAGAAAGCATCCGAGCTG
NOS_rev	CGCGTATTAAATGTATAATTG
**Identification of positive Tks and Tb plants transformed with pH7FWG2:TksMYC2**	
Tks MYC2 FW3	ATGTGAAGGTCATCGGCTGG
GFP RV2	CTTGTAGTTGCCGTCGTCCT
**ChIP-qPCR**	
ChIP TKS CPT2 G-box F	TGAGGGATGACATTTTCTGGGA
ChIP TKS CPT2 G-box R	GACTCGAACCCTTGACCTCC
ChIP TKS SRPP1 G-box F	TATCGGGTTCCAAGTGCAAC
ChIP TKS SRPP1 G-box R	CACACTTTCCACGACAACTGA
ChIP TKS SRPP3 G-box F1	ACCAACTGCTTGCTTTACTCC
ChIP TKS SRPP3 G-box R1	TTATGCAAAAGGTTCAGAGCCA
ChIP TKS SRPP3 G-box F2	GTGGTTTCTGTCCCTGCCTT
ChIP TKS SRPP3 G-box R2	ACAAATTTGCCCTCGTGTGC
ChIP TKS SRPP4 G-box F	GGTTCATCGGTAACAGGTGC
ChIP TKS SRPP4 G-box R	GGCCCCATAGTCGTACAGTT
ChIP TKS GAO G-box F	AACGTGGGTCCGAGATGATG
ChIP TKS GAO G-box R	AACCTTCCTCCAGAGCCATG

Table S2 UHPLC-HRMS metabolite profiling of the *n*-hexane, acetone, and methanol extracts.

n.	Rt (min)	Tentative Assignment	Molecular Formula	[M-H]$^-$ (*m/z*)	RDB
		n-hexane extracts			
H1	3.235	α-linolenic acid (ALA)	$C_{18}H_{30}O_2$	277.2173	4
H2	4.087	linoleic acid (LA)	$C_{18}H_{32}O_2$	279.2330	3
H3	5.054	oleic acid (OA)	$C_{18}H_{34}O_2$	281.2486	2
H4	4.749	palmitic acid (PA)	$C_{16}H_{32}O_2$	255.2330	1
H5	5.440	stearic acid (SA)	$C_{18}H_{36}O_2$	283.2643	1
		Acetone extracts			
A1	0.841	SL-1 (*e.g.*, cynaroside A)	$C_{21}H_{32}O_{10}$	443.1923	6
A2	1.014	SL-2 (*e.g.*, cynaroside A)	$C_{21}H_{32}O_{10}$	443.1923	6
A3	1.274	12-hydroxyjasmonate sulfate	$C_{12}H_{18}O_7S$	305.0700	4
A4	3.932	SL-3 (*e.g.*, taraxacolide hexoside)	$C_{22}H_{34}O_{11}$ [+FA] $C_{21}H_{32}O_9$	473.2052*	6
A5	4.255	SL-4 (*e.g.*, dihydrotaraxinic acid hexoside)	$C_{22}H_{32}O_{11}$ [+FA] $C_{21}H_{30}O_9$	471.1872*	7
A6	4.867	SL-5 (*e.g.*, taraxacolide hexoside)	$C_{21}H_{32}O_9$	427.1974	6
A7	4.905	SL-6 (*e.g.*, taraxacolide hexoside)	$C_{22}H_{34}O_{11}$ [+FA] $C_{21}H_{32}O_9$	473.2052*	6
A8	5.002	SL-7 (*e.g.*, dihydrotaraxinic acid hexoside)	$C_{22}H_{32}O_{11}$ [+FA] $C_{21}H_{30}O_9$	471.1872*	7
A9	5.978	SL-8 (*e.g.*, dihydrotaraxinic acid)	$C_{15}H_{20}O_4$	263.1287	6
A10	6.534	SL-9 (*e.g.*, annuolide D hexoside)	$C_{22}H_{32}O_{10}$ [+FA] $C_{21}H_{30}O_8$	455.1923*	7
		Methanol extracts			
M1	0.493	diOHbenzoic acid hexoside	$C_{13}H_{16}O_9$	315.0722	6
M2	0.624	3-CQA	$C_{16}H_{18}O_9$	353.0878	8
M3	0.687	caftaric acid	$C_{13}H_{12}O_9$	311.0409	8
M4	0.716	caffeic acid hexoside 1	$C_{15}H_{18}O_9$	341.0878	7
M5	0.899	caffeic acid hexoside 2	$C_{15}H_{18}O_9$	341.0878	7
M6	0.972	coutaric acid	$C_{13}H_{12}O_8$	295.0459	8
M7	1.072	5-CQA	$C_{16}H_{18}O_9$	353.0878	8
M8	1.274	caffeic acid	$C_9H_8O_4$	179.0350	6
M9	2.332	dicaffeoyltartaric acid 1	$C_{22}H_{18}O_{12}$	473.0725	14
M10	2.559	dicaffeoyltartaric acid 2	$C_{22}H_{18}O_{12}$	473.0725	14
M11	1.632	*p*-coumaroylquinic acid	$C_{16}H_{18}O_8$	337.0929	8
M12	3.244	caffeoylcoutaric acid 1	$C_{22}H_{18}O_{11}$	457.0776	14
M13	3.320	luteolin 4'-O-hexoside	$C_{21}H_{20}O_{11}$	447.0933	12
M14	3.454	caffeoylcoutaric acid 2	$C_{22}H_{18}O_{11}$	457.0776	14
M15	3.796	3,5-diCQA	$C_{25}H_{24}O_{12}$	515.1195	14
M16	3.857	feruloylcaftaric acid	$C_{23}H_{20}O_{12}$	487.0882	14
M17	4.407	4,5-diCQA	$C_{25}H_{24}O_{12}$	515.1195	14

*Formic acid adduct
