# Supplementary material for: MYC2 influences rubber and sesquiterpene lactones synthesis in Taraxacum species
Source: Planta. 2025 May 24;262(1):5. doi: 10.1007/s00425-025-04719-9 (PMC12103366; doi:10.1007/s00425-025-04719-9)
Supplement: Supplementary file 2 — Supplementary file2 (PDF 476 KB) [file 425_2025_4719_MOESM2_ESM.pdf]

Elio Fantini[1*], Loretta Daddiego[1*], Paolo Facella[*1], Giorgio Perrella[1,2], Linda Bianco[1], Carlo Fasano[1], Fiammetta Alagna[1], Michele Antonio Savoia[1,3], Daniela Rigano[4], Carmina Sirignano[4], Orazio Taglialatela Scafati[4], Severina Pacifico[5], Simona Piccolella[5], Loredana Lopez[1], Francesco Panara[1**]**

***** Equal contribution

****** Corresponding authors

TksMYC2 Interacts with Rubber and Sesquiterpene Lactones Synthesis Genes Promoters and its Overexpression Drives Metabolite Synthesis Changes in Dandelion

[1] Trisaia Research Center, ENEA, S.S. 106 Ionica - Km 419+500, 75026 Rotondella (MT), Italy

[2] Department of Biosciences, University of Milan, Via Celoria 26, 20133, Milan, Italy

[3] Department of Soil, Plant and Food Sciences, University of Bari Aldo Moro, Via Amendola 165/A, 70126 Bari, Italy

[4] Department of Pharmacy, School of Medicine and Surgery, University of Naples Federico II, Via D. Montesano 49, 80131 Naples, Italy

[5] Department of Environmental Biological and Pharmaceutical Sciences and Technologies, University of Campania "Luigi Vanvitelli", Via Vivaldi 43, I-81100 Caserta, Italy

Francesco Panara

E-mail: francesco.panara@enea.it

Telephone: +39-0835-974523

Orcid-ID: https://orcid.org/0000-0001-9596-7262

Loredana Lopez

E-mail: loredana.lopez@enea.it

Telephone: +39-0835-974355

Orcid-ID: https://orcid.org/0000-0002-7129-9893

Figure S3 Output of the FGENESH analysis of the Tks contig utg1303. Online tool available at http://www.softberry.com (Solovyev et al. 2006).

FGENESH 2.6 Prediction of potential genes in Medicago genomic DNA
Seq name: test sequence
Length of sequence: 180642
Number of predicted genes 34: in +chain 20, in -chain 14.
Number of predicted exons 73: in +chain 49, in -chain 24.
Positions of predicted genes and exons: Variant 1 from 1, Score:1862.389258

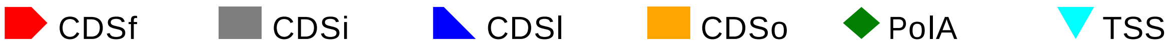

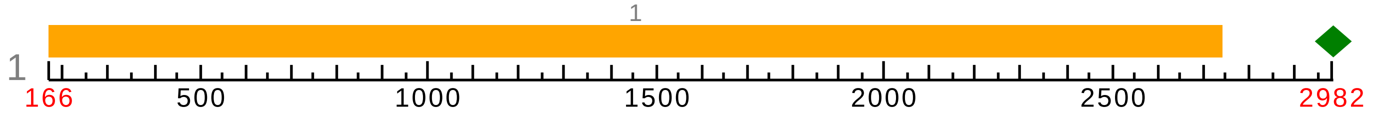

1	+	1	CDSo	166	-	2739	130.01	166	-	2739	2574
1	+		PolA	2982			-1.41				

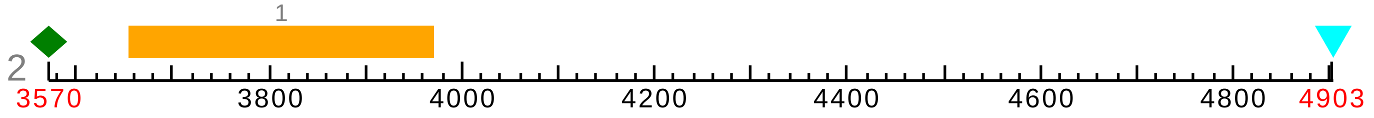

2	-		PolA	3570			4.19				
2	-	1	CDSo	3653	-	3970	23.64	3653	-	3970	318
2	-		TSS	4903			-2.24				

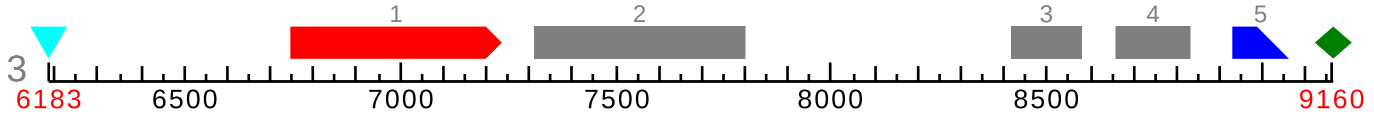

3	+		TSS	6183			-3.14				
3	+	1	CDSf	6743	-	7233	33.55	6743	-	7231	489
3	+	2	CDSi	7308	-	7797	29.41	7309	-	7797	489
3	+	3	CDSi	8413	-	8577	13.12	8413	-	8577	165
3	+	4	CDSi	8656	-	8829	13.59	8656	-	8829	174
3	+	5	CDSl	8926	-	9057	0.48	8926	-	9057	132
3	+		PolA	9160			4.19				

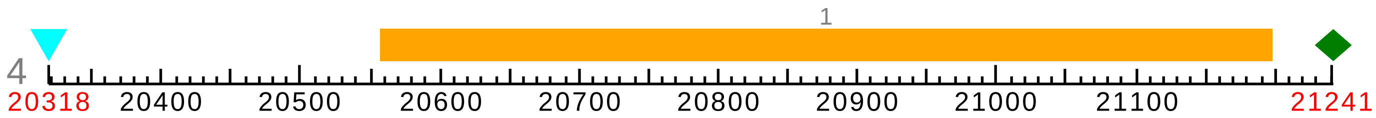

4	+		TSS	20318			-4.34				
4	+	1	CDSo	20556	-	21197	34.18	20556	-	21197	642
4	+		PolA	21241			-1.01				

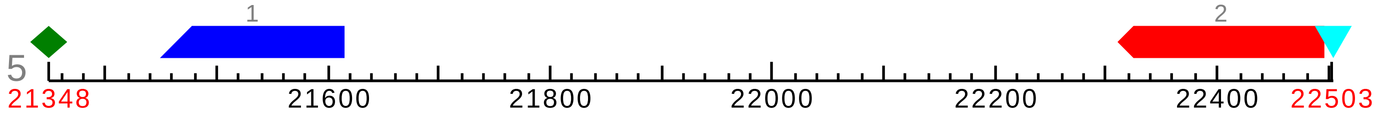

5	-		PolA	21348			4.19				
5	-	1	CDSl	21448	-	21614	-5.23	21448	-	21612	165
5	-	2	CDSf	22309	-	22495	18.92	22310	-	22495	186
5	-		TSS	22503			-3.04				

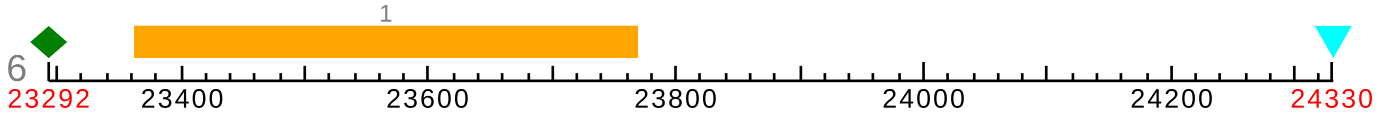

6	-		PolA	23292			-1.71				
6	-	1	CDSo	23361	-	23768	9.26	23361	-	23768	408
6	-		TSS	24330			-1.24				

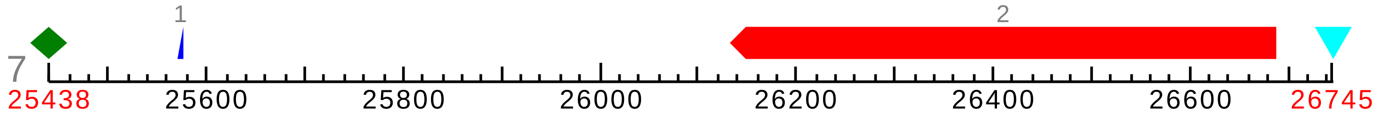

7	-		PolA	25438			4.19				
7	-	1	CDSl	25569	-	25575	-0.77	25569	-	25574	6
7	-	2	CDSf	26131	-	26687	18.87	26133	-	26687	555
7	-		TSS	26745			-1.34				

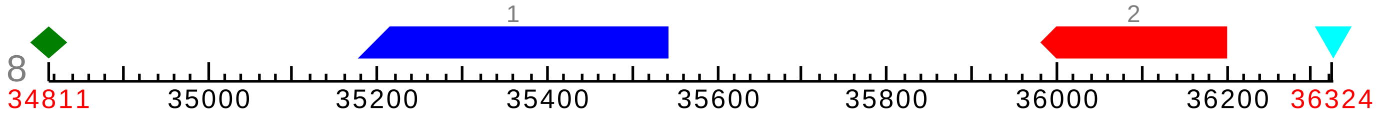

8	-		PolA	34811			4.19				
8	-	1	CDSl	35175	-	35541	12.16	35175	-	35540	366
8	-	2	CDSf	35979	-	36199	2.83	35981	-	36199	219
8	-		TSS	36324			-6.24				

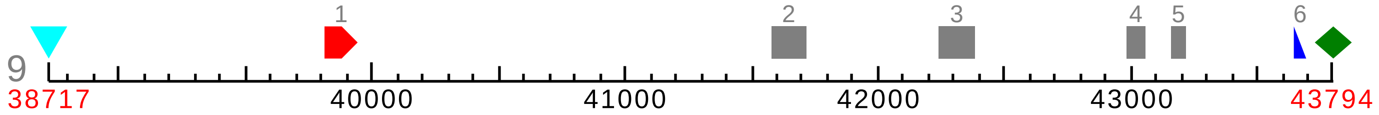

9	+		TSS	38717			-1.24				
9	+	1	CDSf	39807	-	39938	12.92	39807	-	39938	132
9	+	2	CDSi	41573	-	41711	-1.01	41573	-	41710	138
9	+	3	CDSi	42234	-	42378	6.44	42236	-	42376	141
9	+	4	CDSi	42977	-	43051	7.00	42978	-	43049	72
9	+	5	CDSi	43153	-	43211	10.16	43154	-	43210	57
9	+	6	CDSl	43638	-	43687	-0.09	43640	-	43687	48
9	+		PolA	43794			4.19				

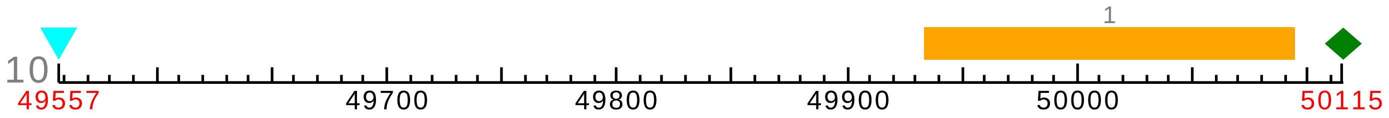

10	+		TSS	49557			-2.24				
10	+	1	CDSo	49933	-	50094	5.80	49933	-	50094	162
10	+		PolA	50115			4.19				

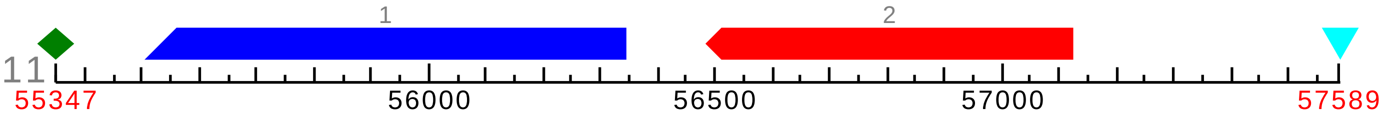

11	-		PolA	55347			4.19				
11	-	1	CDSl	55502	-	56343	20.82	55502	-	56341	840
11	-	2	CDSf	56481	-	57123	36.01	56482	-	57123	642
11	-		TSS	57589			-2.84				

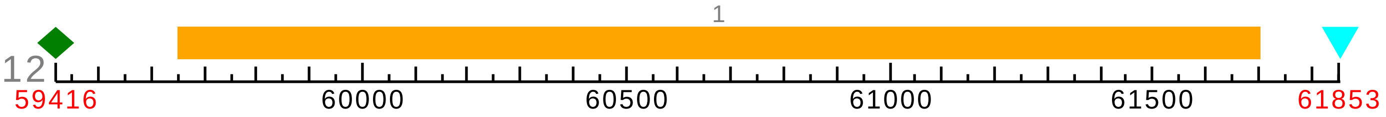

12	-		PolA	59416			4.19				
12	-	1	CDSo	59647	-	61701	142.47	59647	-	61701	2055
12	-		TSS	61853			-4.34				

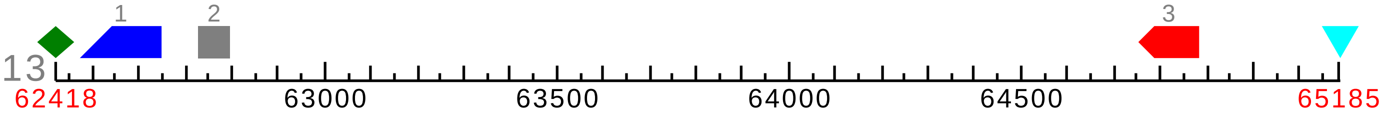

13	-		PolA	62418			4.19				
13	-	1	CDSl	62470	-	62646	0.15	62470	-	62646	177
13	-	2	CDSi	62725	-	62793	4.91	62725	-	62793	69
13	-	3	CDSf	64750	-	64881	6.18	64750	-	64881	132
13	-		TSS	65185			-4.74				

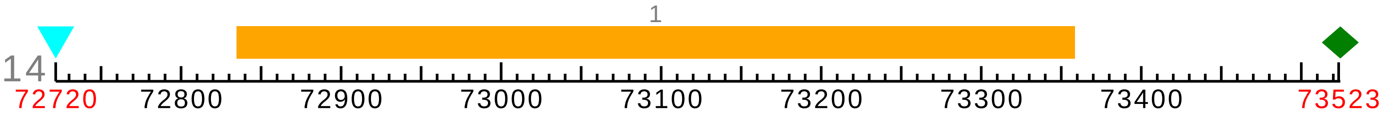

14	+		TSS	72720			-3.44				
14	+	1	CDSo	72833	-	73357	36.38	72833	-	73357	525
14	+		PolA	73523			4.19				

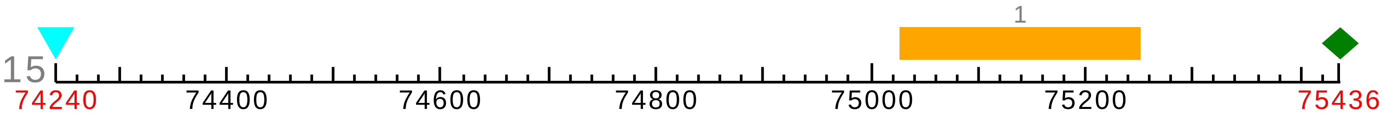

15	+		TSS	74240			-5.24				
15	+	1	CDSo	75026	-	75250	10.38	75026	-	75250	225
15	+		PolA	75436			4.19				

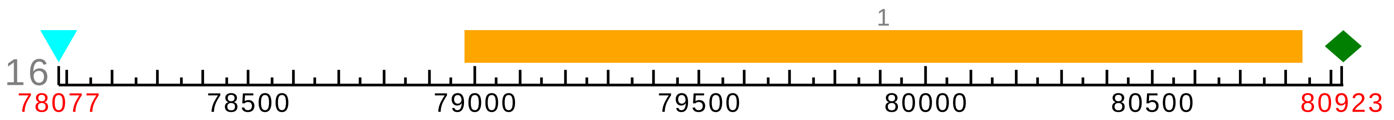

16	+		TSS	78077			-4.54				
16	+	1	CDSo	78976	-	80832	78.15	78976	-	80832	1857
16	+		PolA	80923			4.19				

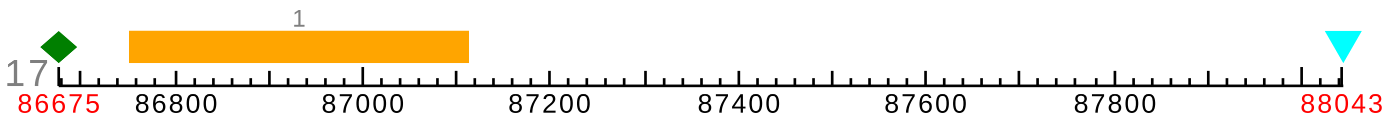

17	-		PolA	86675			4.19				
17	-	1	CDSo	86750	-	87112	2.18	86750	-	87112	363
17	-		TSS	88043			-2.34				

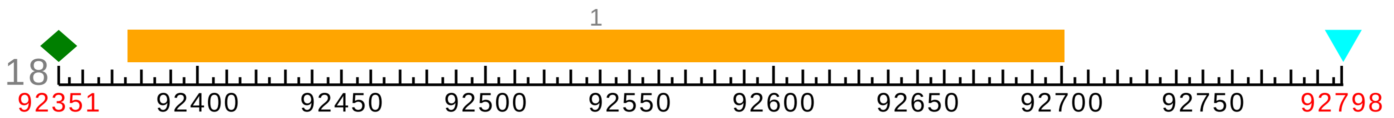

18	-		PolA	92351			-0.61				
18	-	1	CDSo	92375	-	92701	27.20	92375	-	92701	327
18	-		TSS	92798			-5.74				

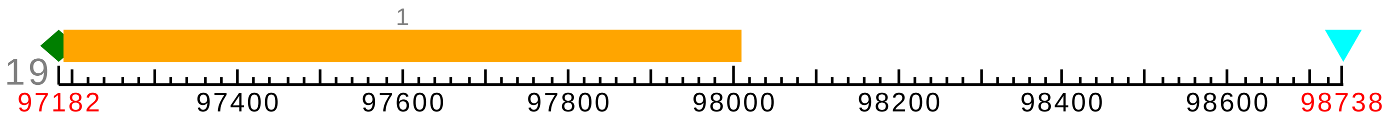

19	-		PolA	97182			-0.61				
19	-	1	CDSo	97188	-	98009	5.81	97188	-	98009	822
19	-		TSS	98738			-2.34				

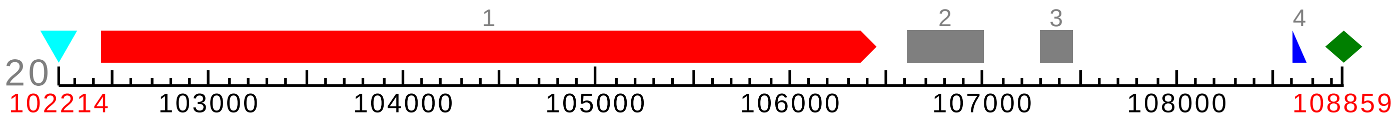

20	+		TSS	102214			-5.34				
20	+	1	CDSf	102433	-	106443	229.93	102433	-	106443	4011
20	+	2	CDSi	106599	-	106996	14.61	106599	-	106994	396
20	+	3	CDSi	107288	-	107457	-1.42	107289	-	107456	168
20	+	4	CDSl	108594	-	108667	2.73	108596	-	108667	72
20	+		PolA	108859			4.19				

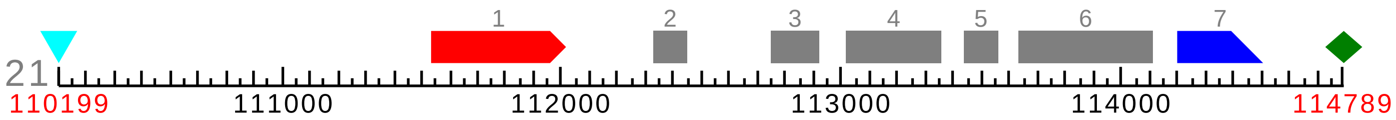

21	+		TSS	110199		-2.64

21	+	1	CDSf	111529	-	112011	41.58	111529	-	112011	483
21	+	2	CDSi	112323	-	112443	2.06	112323	-	112442	120
21	+	3	CDSi	112743	-	112915	3.41	112745	-	112915	171
21	+	4	CDSi	113011	-	113352	13.95	113011	-	113352	342
21	+	5	CDSi	113432	-	113554	12.69	113432	-	113554	123
21	+	6	CDSi	113627	-	114107	17.55	113627	-	114106	480
21	+	7	CDSl	114194	-	114501	13.13	114196	-	114501	306
21	+		PolA	114789			-1.91				

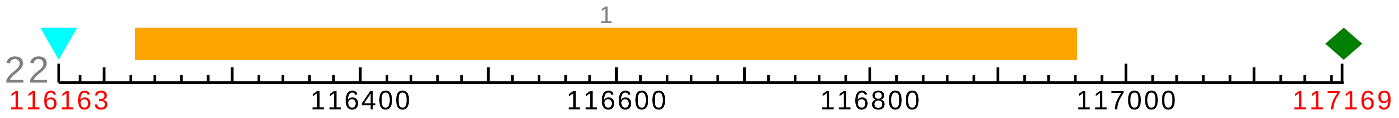

22	+		TSS	116163			0.56				
22	+	1	CDSo	116223	-	116960	112.72	116223	-	116960	738
22	+		PolA	117169			4.19				

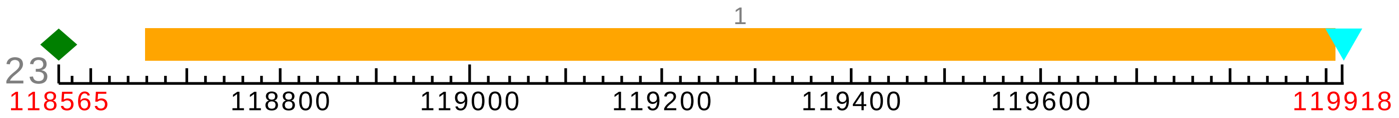

23	-		PolA	118565			4.19				
23	-	1	CDSo	118656	-	119909	199.24	118656	-	119909	1254
23	-		TSS	119918			-2.04				

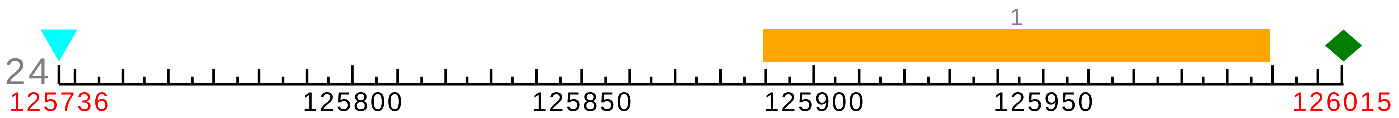

24	+		TSS	125736			-2.84				
24	+	1	CDSo	125889	-	125999	1.76	125889	-	125999	111
24	+		PolA	126015			4.19				

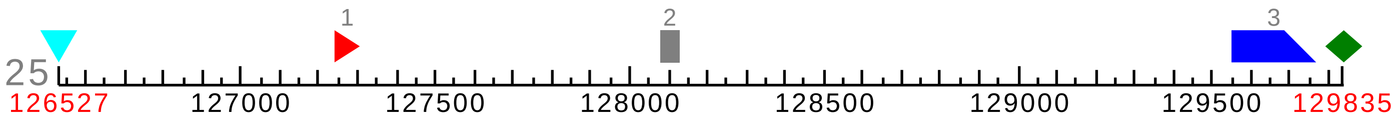

25	+		TSS	126527			-3.14				
25	+	1	CDSf	127237	-	127302	5.26	127237	-	127302	66
25	+	2	CDSi	128075	-	128125	7.35	128075	-	128125	51
25	+	3	CDSl	129546	-	129764	3.39	129546	-	129764	219
25	+		PolA	129835			1.99				

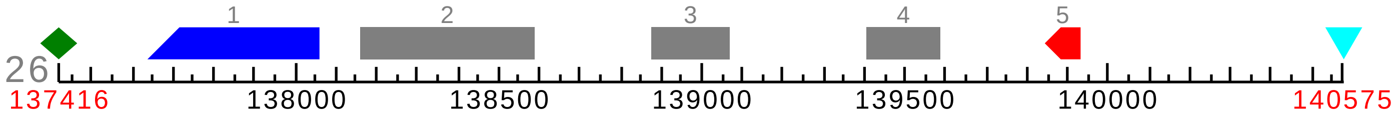

26	-		PolA	137416			4.19				
26	-	1	CDSl	137634	-	138057	35.25	137634	-	138056	423
26	-	2	CDSi	138157	-	138585	28.18	138159	-	138584	426
26	-	3	CDSi	138873	-	139065	-0.97	138875	-	139063	189
26	-	4	CDSi	139402	-	139583	5.75	139403	-	139582	180
26	-	5	CDSf	139840	-	139928	-2.51	139842	-	139928	87
26	-		TSS	140575			-1.84				

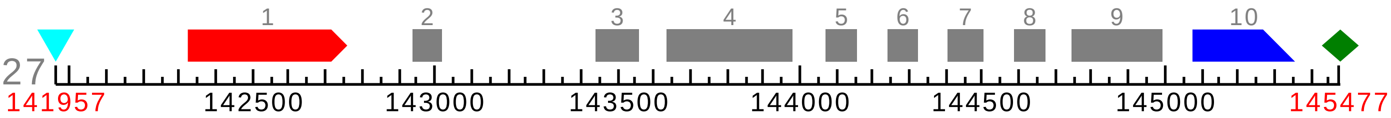

27	+		TSS	141957			-3.34				
27	+	1	CDSf	142319	-	142756	44.58	142319	-	142756	438
27	+	2	CDSi	142935	-	143015	2.72	142935	-	143015	81
27	+	3	CDSi	143436	-	143555	9.70	143436	-	143555	120
27	+	4	CDSi	143632	-	143976	37.56	143632	-	143976	345
27	+	5	CDSi	144067	-	144153	9.00	144067	-	144153	87
27	+	6	CDSi	144237	-	144320	9.65	144237	-	144320	84
27	+	7	CDSi	144401	-	144499	9.68	144401	-	144499	99
27	+	8	CDSi	144583	-	144669	9.24	144583	-	144669	87
27	+	9	CDSi	144741	-	144989	15.95	144741	-	144989	249
27	+	10	CDSl	145072	-	145353	17.12	145072	-	145353	282
27	+		PolA	145477			-1.01				

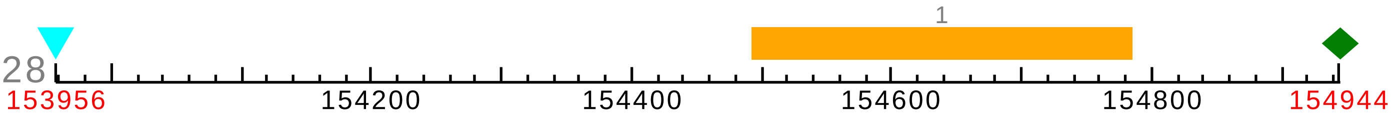

28	+		TSS	153956			-2.84				
28	+	1	CDSo	154491	-	154784	15.76	154491	-	154784	294
28	+		PolA	154944			4.19				

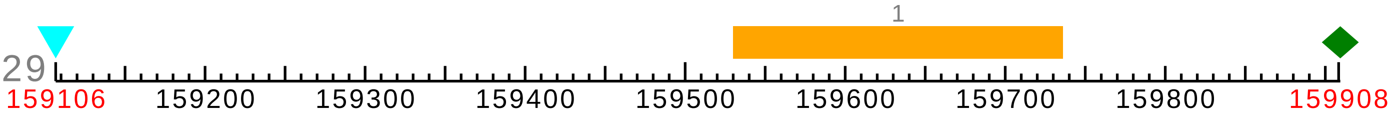

29	+		TSS	159106			-2.84				
29	+	1	CDSo	159529	-	159735	10.79	159529	-	159735	207
29	+		PolA	159908			-0.61				

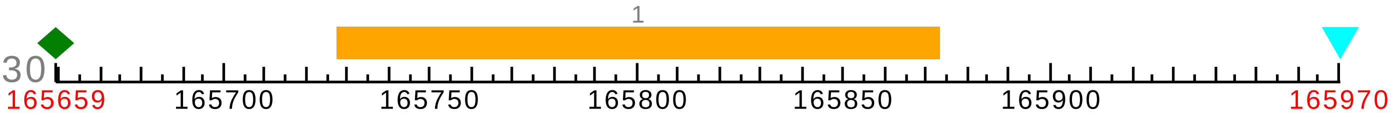

30	-		PolA	165659			1.99				
30	-	1	CDSo	165727	-	165873	6.28	165727	-	165873	147
30	-		TSS	165970			-0.34				

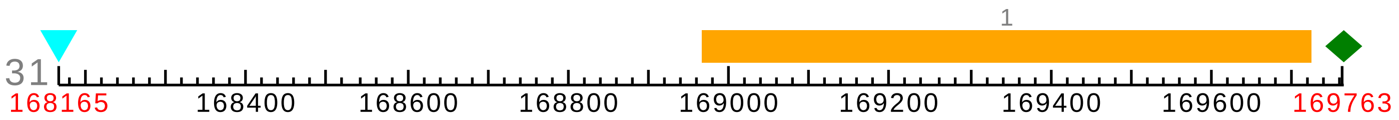

31	+		TSS	168165			-3.34			
31	+	1	CDSo	168965 -	169723	34.71	168965 -	169723	759	
31	+		PolA	169763			-5.21			

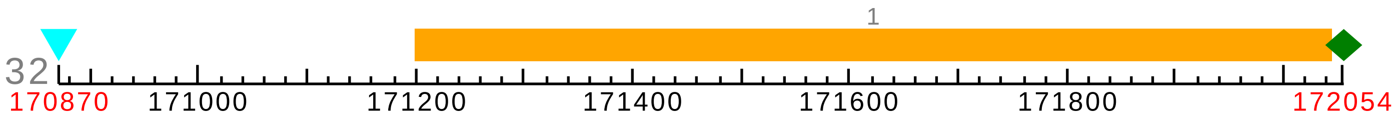

32	+		TSS	170870		-3.54			
32	+	1	CDSo	171198 -	172043	40.89	171198 -	172043	846
32	+		PolA	172054		-3.01			

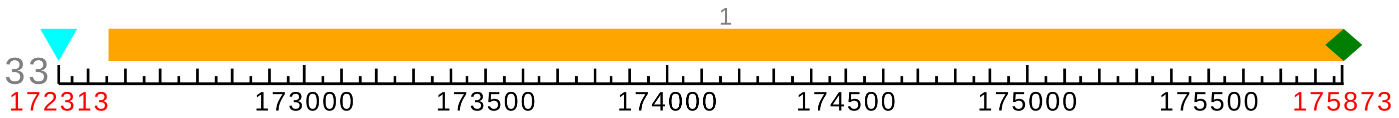

33	+		TSS	172313		-8.04			
33	+	1	CDSo	172451 -	175870	168.87	172451 -	175870	3420
33	+		PolA	175873		-3.61			

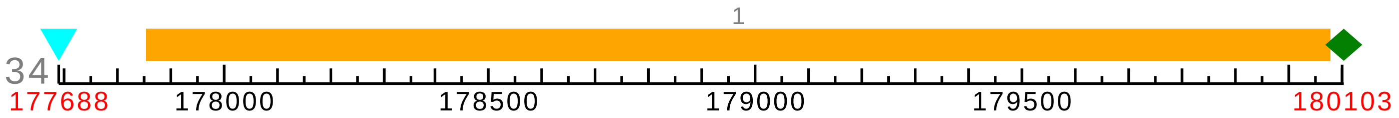

34	+		TSS	177688		-2.94			
34	+	1	CDSo	177853 -	180078	135.03	177853 -	180078	2226
34	+		PolA	180103		-2.81			

Predicted protein(s):

>FGENESH:[mRNA] 1 1 exon (s) 166 - 2739 2574 bp, chain +

ATGGCTGTAGGAATTGTTGTTGAACATCCAGTTGCACATGTTCACACACAAAATGGTTTA
GCTGAATCATTAATTAAACGTCTCCAGCTTATTGCTAGACCACTTATAATGAGAACTAAA
CTTCCTGTTTCTATATGGGGACATGCAATATTGCATGCTGTATCGTTAATTCGCATAAGA
CCAAGTGCATATCATACATATTCTCCCCTCCAACTTGTTTTTGGTCAAGAACCAAATATA
TCCCATCTTAGAATTTTTGGTTGTGCAGTATATGTCCCGATTGCACCACCCCAACGCACA
AAAATGGGGCCTCAAAGAAGATTGGGAATATATGTTGGATATGAAACAGTTTCTATTATA
ATATATCTTGAACCATTAACAGGTGATGTTTTTACAGCACGTTTTGCTGATTGCCAATTT
GATGAATCAGTTTTCCCATCATTAGGGAGAGAAAAGAAAATTCAAGAAAAGAATATTTCT
TGGAATGAACCCTCATTGTTGTATCTTGATCCTTACACAAAACAATGTGAGATGGAAGTT
CGAAAGATACTGAATTTCCAAGAATTGGCAAATCAATTACCTGATGCATTTACTGATACA
AAAAGAGTGACAAAATCATATATTCCAGCTGCTAATGCTCCAGCTCGAATTGAGATTCCA
GATACAAAATCTGAGGATAAAGTCACTCAAGAGTCCAAAACACGCCTGAAGCGTGGAAGA
CCAGTTGGTTCTAAAGATAAGAATCCAAGAAAAGGAAAAAGAATACAAAGCATACTTGAT
CATAAGAAAAATGTTCCTGAAGACAAACAAAATATCATATGTTCCCCTGAAGAGGAAATG

AATGATATAAACAAAGAGATATCAATCAGTTATGGTCAATCAAACATAATATATGACCAA
GATGGAAATATAACTGAAATATTTTCATATTCAGTTGCATGTGATATTATGAATGATGAT
CCTGAACCACAATCTGTTATTGATTGTCAAAACAGATATGATTGGGCTAAGTGGAAAGAA
GCTATGCAAGCTGAATTAAATTCTCTTAATAAGAGAAATGTTTTTGGACCCATTGTTCTC
ACACCTGGAGTTGTGAAACCATTAGGGTATAGATGGATCTTCGTACGAAAAAGAAATGAA
AAGGATGAGATTGTAAGATACAAAGCCAGGCTTGTGGCTCAAGGTTTCTCTCAAAGACTG
GGAATAGATTTTGAGGAAACATATTCCCCTGTTATGGATGCAACTACTTTTAGATATTTA
ATCAGTCTGGCTGTTTCTGAAAATCTAGAGATGCGTCTTATGGATGTTGTTACAGCTTAC
TTGTATGGATCACTTGACAGTGACATTTATATGAAGATCCCAGAAGGATTTAAAATGCCT
GAAGCATTATGTGCAAAACCCAAAGAAATGTATTCTATAATATTACAAAGATCTTTATAT
GGGTTAAAGCAATCTGGACGCATGTGGTACAATCGTTTAAGCAATTATTTGACAAGTAAA
GGTTACAAAAATAATGCTATTTGTCCATGCGTATTTATTAAGAAAACAACATCCGGATAT
GTGATCATAGCTGTCTATGTTGATGATCTAAACATCATTGGAACGAATAAAGAAATTCTT
GAAGTAATTGACCTGTTGAAAAAAGAATTTGAAATGAAAGACCTTGGAAAAACCAAGTAT
TGTCTTGGTTTGCAGATTGAGCATATGCCTAATGGAATACTTGTACATCAATCAAATTAT
ACTGAAAGAGTCTTGAAACGTTTCAATATGAACGATGCCAATTCTTTAAGCACTCCAATG
GTTGTTAGATCATTAAATATTGACAAAGATCCATACCGCCCTTGCGAAGAAAATGAGGAG
GTTCTTGGTCCAGAAGTACCATACCTTAATGCAATTGGGGCCCTTATGTATCTAACCAAT
TGTACTAGACCCGACATTTCTTTTGCAGTAAACTTACTATCAAGGTTCAGTTCATCACCG
ACAAAAAGACATTGGAACGGGATCAAACACATTTTTCGATACCTTCGAGGAACTACTGAT
TTAGGATTATTTTATCCTAATGATTCAAAGCATGGTTTAATTGGTTATGCAGATGCAGGT
TATTTGTCTGATCCTCATAAAGCAAAATCTCAAACTGGATATGTCTTCATGAATGGAGGA
ACTGCAATTTCTTGGCGTTCTCAAAAGCAAACACTCGTTGCCACATCTTCAAATCATGCT
GAAGTGATAGCATTAAATGAAGCTAGTAGAGAGTGTAAATGGTTGAGATCAATGACACAA
CTAATATGGACTTCATGTGGGCTGGATAAAGATAAAGCTCCAACTTTAATCTATGAAGAC
AACGCAGCATGTGTCAGCCAAATGAAAGAAGGATACATCAAGAGCGACAGGACAAAACAT
ATACCTCCAAAGTTCTTTGAATACATTCGAGAACTTATAGAGACTCATCAGGTCGAGATA
AAATATGTTCAATCCAGCAACAATGCAGCAGATCTTTTCACAAAGGCACTTCCTACTGCA
ATATTCAAGAAGCATGTGCATGACATTGGAATGAGACATGTCCACAACATCTAA
>FGENESH: 1 1 exon (s) 166 - 2739 857 aa, chain +
MAVGIVVEHPVAHVHTQNGLAESLIKRLQLIARPLIMRTKLPVSIWGHAILHAVSLIRIR
PSAYHTYSPLQLVFGQEPNISHLRIFGCAVYVPIAPPQRTKMGPQRRLGIYVGYETVSII
IYLEPLTGDVFTARFADCQFDESVFPSLGREKKIQEKNISWNEPSLLYLDPYTKQCEMEV
RKILNFQELANQLPDAFTDTKRVTKSYIPAANAPARIEIPDTKSEDKVTQESKTRLKRGR
PVGSKDKNPRKGKRIQSILDHKKNVPEDKQNIICSPEEEMNDINKEISISYGQSNIIYDQ
DGNITEIFSYSVACDIMNDDPEPQSVIDCQNRYDWAKWKEAMQAELNSLNKRNVFGPIVL
TPGVVKPLGYRWIFVRKRNEKDEIVRYKARLVAQGFSQRLGIDFEETYSPVMDATTFRYL
ISLAVSENLEMRLMDVVTAYLYGSLDSDIYMKIPEGFKMPEALCAKPKEMYSIILQRSLY
GLKQSGRMWYNRLSNYLTSKGYKNNAICPCVFIKKTTSGYVIIAVYVDDLNIIGTNKEIL
EVIDLLKKEFEMKDLGKTKYCLGLQIEHMPNGILVHQSNYTERVLKRFNMNDANSLSTPM
VVRSLNIDKDPYRPCEENEEVLGPEVPYLNAIGALMYLTNCTRPDISFAVNLLSRFSSSP
TKRHWNGIKHIFRYLRGTTDLGLFYPNDSKHGLIGYADAGYLSDPHKAKSQTGYVFMNGG
TAISWRSQKQTLVATSSNHAEVIALNEASRECKWLRSMTQLIWTSCGLDKDKAPTLIYED
NAACVSQMKEGYIKSDRTKHIPPKFFEYIRELIETHQVEIKYVQSSNNAADLFTKALPTA
IFKKHVHDIGMRHVHNI
>FGENESH:[mRNA] 2 1 exon (s) 3653 - 3970 318 bp, chain -
ATGGAGTTTCCGGTCATTTCCCGAGTTCGGTCGACGAATGTATCACCGACGACATCTTTG
GCTTCGTGGAGTAGCAGAGTTGTTGTTAGGATGCCGATGAAATTAAAGAAGTGGAGATAC

GTTGCCGGAAAAAGAGTGGTGGATGTGAGAGCTTCGCTGGACAGGGGCGGCGGTGATCGG
GGAGATAGAAATAGCGGTGGTTGCTGGAACACCGGCACGGAGGTGATTACATATAATAAT
AAGAGCTTTTCAGATGCAGACTACCCTGCTTGGGACAATAATGGTGCTGGTGTTAGAGTC
ACCTATGGAATTGGTTAG
>FGENESH: 2 1 exon (s) 3653 - 3970 105 aa, chain -
MEFPVISRVRSTNVSPTTSLASWSSRVVVRMPMKLKKWRYVAGKRVVDVRASLDRGGGDR
GDRNSGGCWNTGTEVITYNNKSFSDADYPAWDNNGAGVRVTYGIG
>FGENESH:[mRNA] 3 5 exon (s) 6743 - 9057 1452 bp, chain +
ATGGATGATATAAGCTTTGCTTTAGTCCGAAAAGATAAAAGTTTAAATCTTGAGGTTGTG
TGGACAACAATTGTTTACCAAAATTCGGTAATTTTACAGATGGGTGACGTGGCAAAGGAC
TTAGCGGCGGGAACGGTTGGAGGTGTAGCCCAGTTGATAGTTGGGCATCCTTTTGATACC
ATCAAGGTGAAACTCCAAAGTCAACCGACCCCATTACCTGGTCAACTCCCCAAATACTCC
GGTGCCATAGATGCCGTTAAAAAAACATTGGCCGCTCAAGGTGCCGGAGGTCTGTACAAA
GGCATGGGAGCCCCACTTGCCACGGTGGCAGTCTTCAATGCGGTGTTGTTCTCAGTTAGA
GGACAAATGGAGGCCCTTTTAAAGTCCGAACCCGGTGCATCTTTGACCGTGAAACAACAG
TTCATCGCCGGAGCCGGTGCTGGTTTCGCCGTCTCCTTCTTGGCCACCCCAACTGAGTTA
CTTAAATGCAGATTGCAGGCACAAGGCGCAGGTGCGGCGGTGGCAGAAGGTGCCGCAGCC
GCCACGGCGGCGTTGAAGTACAGTGGTCCGATGGACGTGGCTAGACAAGTGCTAAAGTCA
GAAGGAGGTGCACTAGGTTTATTCAAAGGGTTCTTTCCGACAATAGTTCGTGAAGTCCCT
GGAAACGCTACCATGTTTGGCGTCTACGAAGCTCTGAAACAGTACATCGCCGGCGGAACC
GACACTTCCGGGTTAAGCAGAGGCTCCCTGATGATGGCCGGAGGGTTAGCCGGAGGTGCG
TTTTGGATTTCCGTTTACCCAGCTGATGTCGTCAAGAGTGTGATCCAAATCGATGATTTT
AAGAATCCAAAGTATTCTGGGGCATTTGATGCATTCAAAAAGATTCTTAAAACTGAAGGA
GTTAGCGGTTTATATAAAGGTTTCGGACCTGCAATGGGTCGAAGTGTACCCGCTAATGCT
GCTTGCTTTCTAGCCTATGAAATAAAGCTTATCACAAAGAACCCTAATTTGTCGAGATGG
CGATATATTTTTAAATTTGGAAGAAAGAACAGCGAATGTGTTCCGTTGAATGAAACTATT
GCGGATGCCAACAACAAAAACTATTTGCTGGATCGGATTGAGTCCCTTGAAGATCGTCTA
ATTCAGCTTTCTTTAGAAATTGAAACCCGAAGAACATCACGCACTTCAACCACACCATCA
ACGATTCCTGCAACACGGGAATTGCCGATTTCTTCATACCCTGTTTTCAACAATCCTAAG
CCAAAATGCAAGCGTGTAGCTTCTGATGCTCTACCCATCAGCACTGGTGGCGAATTACAG
AAGAGATCTGAAGGACTGGATAAGAAGAAAAAACGGAGGAATGGAAGGAGTAAGGACGAG
AATTGTAAGACATTTAAGAATGGGAAGAAAAAAGCCCGCAGTTGGCCACACCTTAAGATA
TTAGGTTGCTAG
>FGENESH: 3 5 exon (s) 6743 - 9057 483 aa, chain +
MDDISFALVRKDKSLNLEVVWTTIVYQNSVILQMGDVAKDLAAGTVGGVAQLIVGHPFDT
IKVKLQSQPTPLPGQLPKYSGAIDAVKKTLAAQGAGGLYKGMGAPLATVAVFNAVLFSVR
GQMEALLKSEPGASLTVKQQFIAGAGAGFAVSFLATPTELLKCRLQAQGAGAAVAEGAAA
ATAALKYSGPMDVARQVLKSEGGALGLFKGFFPTIVREVPGNATMFGVYEALKQYIAGGT
DTSGLSRGSLMMAGGLAGGAFWISVYPADVVKSVIQIDDFKNPKYSGAFDAFKKILKTEG
VSGLYKGFGPAMGRSVPANAACFLAYEIKLITKNPNLSRWRYIFKFGRKNSECVPLNETI
ADANNKNYLLDRIESLEDRLIQLSLEIETRRTSRTSTTPSTIPATRELPISSYPVFNNPK
PKCKRVASDALPISTGGELQKRSEGLDKKKKRRNGRSKDENCKTFKNGKKKARSWPHLKI
LGC
>FGENESH:[mRNA] 4 1 exon (s) 20556 - 21197 642 bp, chain +
ATGGAGAAAGGCGAAGCCTCCTCCACGCCGGAAGGAGACAAACCACCTTCTCCACCGCCG
CAAACCATCACTAAAACGACGGGCCACCACCTGTCCGCCCCACCTGGTTTGACCAACGAC
GAGTTCAACGAGTTGAAACATTTTATAATCAACTTCCACACCTACCATCTCACATCATCC
CAATGTTCCTCCCTACTCGCCCAACACATCCACGCGCCGGTCGACGTCGTTTGGTCAGTC

GTCCGTCGATTCGACAAACCGCAGACCTACAAGCACTTCATCAAGAGCTGCACCGTTAGT
GAAGGCTTTACAATGACGGTGGGGTGCACGCGTGACGTCAACGTCATATCTGGATTGCCG
GCGGCAACCAGCACGGAGCGGTTAGACTTGTTGGATGATGAGAACCACGTGTTGGGGTTT
ACTATCATCGGCGGTGAACACAGATTGAGGAATTACCACGCTGTGACGACGGTGCATGAT
ATAACTAGGGCGGTGGATTCACGGCCGATCACGGTGGTTTTGGAATCGTATGTTGTGGAT
GTGCCGGAGGGGAACACGGAAGAGGACACGCGGCTTTTCGCTGACACGGTGGTGAAGTTA
AATCTGCAAAAACTAGCGTCTGTAACCGAAGCCATGGCATGA
>FGENESH: 4 1 exon (s) 20556 - 21197 213 aa, chain +
MEKGEASSTPEGDKPPSPPPQTITKTTGHHLSAPPGLTNDEFNELKHFIINFHTYHLTSS
QCSSLLAQHIHAPVDVVWSVVRRFDKPQTYKHFIKSCTVSEGFTMTVGCTRDVNVISGLP
AATSTERLDLLDDENHVLGFTIIGGEHRLRNYHAVTTVHDITRAVDSRPITVVLESYVVD
VPEGNTEEDTRLFADTVVKLNLQKLASVTEAMA
>FGENESH:[mRNA] 5 2 exon (s) 21448 - 22495 354 bp, chain -
ATGGTTGGAATTGAGATACGCCCGTGTTCACTGAACAAAATGGGACAAATATACCCCTCC
AAAAGAAATCGGGACTGTGACCTCGCTTCTTCCTCCCTTCTCATCGTCGCTTCACTCTCA
TCGTCGCTTCTTCCTCGCTGCCGCGACCTCGCTTCTTCCTCCCTTCTCGGCCGACATCCA
GGTTCCGGATTGAATTACAGCACAAGTTTTGGCAGCCAAACGTGCAACATGATTCAATTA
CAGCACAATTCAATTACCACAGTATTCAATTACAGCACATTCAATTCCTCAGCATCAAAC
AGCCCCAAGAAAATAATAGTAAACATTCACAAAGTATTCCATTATGGACATTGA
>FGENESH: 5 2 exon (s) 21448 - 22495 117 aa, chain -
MVGIEIRPCSLNKMGQIYPSKRNRDCDLASSSLLIVASLSSSLLPRCRDLASSSLLGRHP
GSGLNYSTSFGSQTCNMIQLQHNSITTVFNYSTFNSSASNSPKKIIVNIHKVFHYGH
>FGENESH:[mRNA] 6 1 exon (s) 23361 - 23768 408 bp, chain -
ATGGGTATAGATGTGCCAAGTGTTTTGTGTCTTTTATGCGAAAAAGAGGTAGAGACTATC
CCACATCTTTTCTTTGATTGTGAGTGGTCAAGATGGATGTGGACTAGAGCGGGTCTTTGG
TGGAAGACTTCCATTCCGAGGATAAACAAGTTAGTGGATTTGCTTCAATGGGCGGAGGAT
ATCAAAAAGGACAAAAAGACGAAGATTATTTTTAGAGTTTCCATCTTTGCGGTGATTAAA
CAAATATGGACGACTCGTAATGAAATCATATTCAAGAAGGTTAACCCGGATAAAAATAGG
TGTTGGAATCAAGTTCTTGATTTTAGTTTCTTTTGGTTGTGTAATACGAGTAGATTAGTT
TCTATGGATTGGAATAATTGGATTTCCAATCCCCTTGTAAACGACTAA
>FGENESH: 6 1 exon (s) 23361 - 23768 135 aa, chain -
MGIDVPSVLCLLCEKEVETIPHLFFDCEWSRWMWTRAGLWWKTSIPRINKLVDLLQWAED
IKKDKKTKIIFRVSIFAVIKQIWTTRNEIIFKKVNPDKNRCWNQVLDFSFFWLCNTSRLV
SMDWNNWISNPLVND
>FGENESH:[mRNA] 7 2 exon (s) 25569 - 26687 564 bp, chain -
ATGGATAATCAAGAACCTTCAACTCCCACATCAAACCAAATCCCTACCACAACCCTAGGA
TTTGGTCTATATTCGGGCTATGTAACAATCTTGACGGGGTCACCACAATTACCATACCAA
AGATCATTCCCCGCCACGACATCTCCCCCGATCCAATTCCAAAACATCGGAGTACAAAAT
GTTCACCAACCTCTTGTTCCTCAATTCCAACATCAATTCGAACCTCAATTCCAAACTCAA
CCCACCGAAACCGAAGATGTTGTGTATGAGACACAACCCGAGTCTATGCCCCAATCTAGC
CGGCAAAAAAAGACTCGCAAGGGAAAGACTAAAACCGCTCAAGAAAAAGCCGTGACATGG
ACCCCGGAGAAACTCGTGTGCCTAGCGGATGCTTGGTGTTTGACATCCAAAGACTCCATA
AAAGGCAACGGCAATAAAAAAGACGGGTATTGGGAGGCAATTCGAAAGTTGTTCCACACA
TTAATGAAGAGGGCAGACTACCGCAACACGGATGCACTAAGCAGCAAGTGGACACTAATA
AGGCCAAGGCTATCGAGGTTTTAA
>FGENESH: 7 2 exon (s) 25569 - 26687 187 aa, chain -
MDNQEPSTPTSNQIPTTTLGFGLYSGYVTILTGSPQLPYQRSFPATTSPPIQFQNIGVQN
VHQPLVPQFQHQFEPQFQTQPTETEDVVYETQPESMPQSSRQKKTRKGKTKTAQEKAVTW

TPEKLVCLADAWCLTSKDSIKGNGNKKDGYWEAIRKLFHTLMKRADYRNTDALSSKWTLI
RPRLSRF
>FGENESH:[mRNA] 8 2 exon (s) 35175 - 36199 588 bp, chain -
ATGGGCAACATGGTGATTGGTAGTTTTTTTGGAACCGGCGGAGTTAAGTTAGTGCTGGGA
GGGTCAGTTAAGACCGCGATGGCGCCCCCAGCCGTCACCACCACTTTGGTCACTCCGAAG
AGCAACCCACCGATCTTTAGGAACCCCCGGACAACTGGGGTAAACCCGGATGCATCACCC
CAGGATTCGAACCTGAGACCTCCACTAGGGAAGCGCAAGACACTTGATGAAGATCTGATT
GGGGTTTATAATCTCTCGGGACCTATACCGACGGATAAAGAGAGAGAGAGAGGAGAGAGA
GTCGATGGAGGTCTCAATCGAAATGTGTTACGGCTAATTGCTCAACCTGCTTTGCTTTTT
CCTCCTCTTACACAAGCCGATGGAGTTGAACTTCAAGACGAGCATCTGAATGGTTACAGT
TCAAACCCTAAAAAGCAGAGAGAAACGCCAACAATTGAAGCGACGATTGAAGCAACCGCC
CAAGGGATAGCGTCGATGCTCTGTGCACACGGACCTGAAGTCGAATGGAGGATCTGCATT
ATTTGGGAAGCGGCGTACATGAGAACAAGTTACCAGATCTGGTCCTAA
>FGENESH: 8 2 exon (s) 35175 - 36199 195 aa, chain -
MGNMVIGSFFGTGGVKLVLGGSVKTAMAPPAVTTTLVTPKSNPPIFRNPRTTGVNPDASP
QDSNLRPPLGKRKTLDEDLIGVYNLSGPIPTDKERERGERVDGGLNRNVLRLIAQPALLF
PPLTQADGVELQDEHLNGYSSNPKKQRETPTIEATIEATAQGIASMLCAHGPEVEWRICI
IWEAAYMRTSYQIWS
>FGENESH:[mRNA] 9 6 exon (s) 39807 - 43687 600 bp, chain +
ATGGGATCTGAGTGGGTTTCTTCCGGCGGATTCGAGAGACACGGCTGGAGTTTTGTTTTT
CCGGCAACAGCGATTTCCGGCAGCAGAGGTTCCGGCGAGAGATGGAGTGTTTCAGATGCT
CCTTCAGTTCAGGTTCAAGATCCTTTCACAGAAAAACCCGCCACACTTGCAAAACCTACT
CTTTTTGTAACGACAAGATTACTATTTCACACTATCCCTCTGCTTCACTCAATTGCAAAA
ATAAATAAATTTACAGAGCAGTGGTTTTCAGATATGGATCGGTATCAGAGGGTAGAGAAG
CCAAGGACCGATGCGCCGATTAACGAGAATGAGATTAGTATAACCACTCAAGGGAGGATG
TGGAACTACATTACTTACGCCATTACTCTTGTCCAGGTCTCTTTTTTCTCACAATTTCTT
GAAACTACCCGCCATGTGTCAGTGATTACAATTACCTTGTCAAGAAAGGAGCTTGACACG
TCATCCATCAGGTATCAACTACCAAATGCAACTGATCAAGTGAAACCGTTAGTAGAATAC
GAGCACGAAGGTTCACAAGTGAAAGATATGAAGACAAGACCGTCGTATGAAGCCGAGTAA
>FGENESH: 9 6 exon (s) 39807 - 43687 199 aa, chain +
MGSEWVSSGGFERHGWSFVFPATAISGSRGSGERWSVSDAPSVQVQDPFTEKPATLAKPT
LFVTTRLLFHTIPLLHSIAKINKFTEQWFSDMDRYQRVEKPRTDAPINENEISITTQGRM
WNYITYAITLVQVSFFSQFLETTRHVSVITITLSRKELDTSSIRYQLPNATDQVKPLVEY
EHEGSQVKDMKTRPSYEAE
>FGENESH:[mRNA] 10 1 exon (s) 49933 - 50094 162 bp, chain +
ATGGATAGGAAGTACTTGCAGACCGAGTCAAATCGATCATCCACTGCTGGAAACCATTTC
ATCTCTGATATGCCGCCATCACCTACCACCATCACTTCTATCTCCGACGATTCTCTCTGT
CTCTGTGACAGCTGGAGAAATTTATGTTTTAAACCAATATAA
>FGENESH: 10 1 exon (s) 49933 - 50094 53 aa, chain +
MDRKYLQTESNRSSTAGNHFISDMPPSPTTITSISDDSLCLCDSWRNLCFKPI
>FGENESH:[mRNA] 11 2 exon (s) 55502 - 57123 1485 bp, chain -
ATGGGTTTTGGAACGAGGTGGAGAGCTTGGATTCAGGCTTGTCTCTCTTCCTCTCGCGCT
TCGGTTGTGGTTAACGGCTGTCCCACGAAGGAGTTCAACGTGTCTAAAGGGGTTCGCCAA
GGAGATCCTCTTTCCCCGTTTCTCTTCATCGTGGCTATGGAAGGGCTTAATGTAGCATTG
AAATCGGCTAAAGAGAAATGTTTTTTTAAAGGTGTTCAAATCTCCGACACCGAGCTTTCA
CACCTATTCTATGTCGATGATGCCCTCTTTATGGGTGAATGGTCCAAATCGAATATCACA
AACCTCGCGCGTATACTACGGTGTTTTCATGTCTCCTCGGGCTTGAAGGTTAATTTTTCC
AAATCCAAAGTTTTTGGGATTGGTGCTGGTCATGCGGAAACTGAAGAATGGGCGGATATC

ATTGGATGTGTTGCCGGGTCTCTTCCCTTCCAGTATCTGGGAGTACCGATTGGAGCTAAT
ATGAACTTGATCAAGAATTGGAAACCGATTATTGAGAGATTTAAATCTAAGCTGTCCACT
TGGAAATCCAATAGTCTCTCGTTTGGAGGTCGCATTACTCTGGTGAAGGCTGTCCTCGGC
AGTCTGCCCATTTTCTATCTCTCCATATTCAAAGCCCCTCAAGGCTCGATATACGCATTA
AACATCGGTCTCATCGTGAAATGGTGGTGGCGGCTTAAAAATCAACCATTCTCATTATGG
TGTAAGGTCATCAGTGGTATTCATCAGCTTAGTGGTAAACCAGAGCATCAGCTTGCGGAC
CCGAAAATTCCAGGAGTATGGAAAAATATCGCCAACGCCAAGTCAGACATTGGGAAAATT
AACATGAATGTTTATGAAAATTTTAAAGTGCAGGCTGATACTGTTCAAAACAGGAATAAT
TGGAGTTGTTTGCTAAATCAAGATGACAATTATACGGTAGAGGCTCTCAGAATAAAGGTG
GACCAATCCAAATTCAAGGAGTCCCCGCAGGCGGTTTCGTGGAGAAAGGAGATCCCGATT
AAGGTGATTGGCTTCGTTTGGAAAGCTGCCAAAGGAAGAATCGCCTCCAAGGCTGCCCTC
TCTTCTCGTGGGATCCCCTTGGCATCAACTGATTGCGGGAGGTGTGGCGGCTTGGAAGAT
GCAGATCATATCCTCGTGTCTTGTCCCTTTGCTATCCAGGTGCGTAATTGGATTAGTGAT
TGGTGTGGTGTCCCGCTGCCCTCTTTCAATACCGTAAAAAATATTCTGGAGTTTGCCAAT
CATTGGGGACAATGTCCGAAAAAAAGAAGGCTTTTGAATATGGTTATTTATGGGATGCTA
TGGAGACTTTGGATATTCAGAAACGAAAGAACCTTCAAAACGAGGAATGTCTCCATAGCT
GGGGCTAGAGACGACATTAGATCCACGGTGTTTTTTTGGGCTAAGCACAGGGGTAAACTA
GGAATTTGTAATTGGTCTAATTGGTGTCATTCGTCTTTCGTGTGA
>FGENESH: 11 2 exon (s) 55502 - 57123 494 aa, chain -
MGFGTRWRAWIQACLSSSRASVVVNGCPTKEFNVSKGVRQGDPLSPFLFIVAMEGLNVAL
KSAKEKCFFKGVQISDTELSHLFYVDDALFMGEWSKSNITNLARILRCFHVSSGLKVNFS
KSKVFGIGAGHAETEEWADIIGCVAGSLPFQYLGVPIGANMNLIKNWKPIIERFKSKLST
WKSNSLSFGGRITLVKAVLGSLPIFYLSIFKAPQGSIYALNIGLIVKWWWRLKNQPFSLW
CKVISGIHQLSGKPEHQLADPKIPGVWKNIANAKSDIGKINMNVYENFKVQADTVQNRNN
WSCLLNQDDNYTVEALRIKVDQSKFKESPQAVSWRKEIPIKVIGFVWKAAKGRIASKAAL
SSRGIPLASTDCGRCGGLEDADHILVSCPFAIQVRNWISDWCGVPLPSFNTVKNILEFAN
HWGQCPKKRRLLNMVIYGMLWRLWIFRNERTFKTRNVSIAGARDDIRSTVFFWAKHRGKL
GICNWSNWCHSSFV
>FGENESH:[mRNA] 12 1 exon (s) 59647 - 61701 2055 bp, chain -
ATGACGGATTACCGGCTACAGACTATGAATCACTGGACCTCTGATGAGAACGTTTCCATG
ATCGATGCCTTCATAACTTCCGACATGGCATCCATCTGGGCCAATCCGGCTGCTTCTCAA
TCTCATACCAATACGACGGCGGTACCTGTTCCTCCGGCGTCATCATCTGCTTCAACCTCC
GATCCGCACAAGATAGTCAACGATTTCAATCCGGATACTTTACAGCAACGCCTCCAGGGG
TTGATTGATACGGCACGGGAGTCGTGGACTTACGCTATATTCTGGCAGTCGTCTCACGTC
GATTACACGGATACTCCGGTGTTAGGGTGGGGAGATGGGTATTACAAGGGGGAGGTGAAT
AAGGTGAAAACGAAGCCGTCGGCGACTTCTTTGGCGGAGCAACAGTACCGGAAGAAAGTG
CTCCGGGAGCTTAATTCTCTGATCTCTGGCTCACAGGCGCCGGAAAGCGATGCCGTGGAT
GAGGAAGTTACTGATACGGAATGGTTCTTTCTTATCTCCATGACGCAGTCGTTTGTCAAC
GGTAACGGTCTTCCTGGTCAGGCGGCCTTTAGTAACCAACCGGTTTGGGTGGCCGGACGG
GAACGGTTGATGGCATCTCACTGCGAACGAGCTCGCCAAGGTCAAGGTTTTGGATTGCAA
ACAATCGTATGTATCCCTTCCGCCGATGGGGTTATTGAATTAGGTTCAACGGAGTTGATA
TTTCAGAGTGCAGATGTGATGAAGAAAGTCAAGGTTTCGTTTAATTTCAGCGGGGATTTG
ATGCAGATCAACACAATACAGCCTGCCGGAGGAGATAACGATCCCTCGTCGATTTGGCTT
ACAGATCCGGTGGCTACATCTACGGTGACTACCGACATCGCACCAATCAAGGATTCCGTT
GACATAATCGGCTCTCAGACGACAAGTGTCATTCCATCTATTACTTCCCACGTTCCAAAC
CCTAGCTCAAGTTCGTTACCTGAAAACCCCAGATACATTAATAATCCCAATCGTGATTCC
CTACAAAATCAAGGAGTTTTTGGCAGCAGGGATTTGAATTTCTCTGAATTTGGATCGGTC
GACAGAGCAACTGCTGGCAGAAATGGGAACACTTTATACCCTAAGCCAGAATCCGGCAGA

ATCCTGGATTTCAGCGAGAGTAAAAGGAGTTCTACAAACAACGCAGCGCTGTTCTCCGGT
CAATCCCAGTTCCTCGGAGCAGAGGAGAACAACAACATCAATAATAAGAACAAGAGCAAG
AAGAAGAGATCGCCAGGTTCGTGCGGCAGCAACGAAGACGGGATGCTTTCTTTCGTCTCC
GGTGTGCTTCCATCGTCTAGTATGGGGAAATCCGATGGTGGCGCTTTCACCGGAGCTGAT
CCCGACCATTCAGATCTAGACGCATCGATAATCAGAGAAGTAGAAAGCATCCGAGCTGTG
GAACCGGAGAAAAAACCACGAAAACGAGGACGAAAACCGGCGAACGGCAGGGAGGAGCCA
TTGAATCATGTCGAAGCAGAGAGACAGAGGAGAGAGAAACTAAACCAGCGATTCTACGCT
CTACGCGCCGTCGTCCCAAACGTCTCAAAAATGGACAAAGCCTCCCTTCTCGGCGACGCC
ATATTATACATCAACGAACTCAAATCAAAGCTCGACAACACCGAATGCGACAAAGAAGAA
CTAAGAAACCAAATCGAAGAACTAAAAAGAGAATTACTAAGCAAAGAATCCCGGCACTCA
TCTTCCTCCGCCATCTCACTCCCGGAAGACATGAAAACGTCGACCACCACCCACCAACCC
CCAATGAACTTAGACGTAGATGTGAAGGTCATCGGCTGGGACGCCATGATTAGGGTTCAA
TGTAACAAAAAGAACCACCCTGCCGCCCGGCTAATGGCGGCTTTGAAAGACCTCGATTTC
GAAGTCAACCATGCTAGTGTGTCGGTGGTGAATGATTTGATGATCCAACAAGCCACCGTG
AAAATGGGCGGTCGGTTGTACACTCAAGATCAGCTCCGATTAGCCTTAACAAACAGATTT
TCAGATCCATTGTAA
>FGENESH: 12 1 exon (s) 59647 - 61701 684 aa, chain -
MTDYRLQTMNHWTSDENVSMIDAFITSDMASIWANPAASQSHTNTTAVPVPPASSSASTS
DPHKIVNDFNPDTLQQRLQGLIDTARESWTYAIFWQSSHVDYTDTPVLGWGDGYYKGEVN
KVKTKPSATSLAEQQYRKKVLRELNSLISGSQAPESDAVDEEVTDTEWFFLISMTQSFVN
GNGLPGQAAFSNQPVWVAGRERLMASHCERARQGQGFGLQTIVCIPSADGVIELGSTELI
FQSADVMKKVKVSFNFSGDLMQINTIQPAGGDNDPSSIWLTDPVATSTVTTDIAPIKDSV
DIIGSQTTSVIPSITSHVPNPSSSSLPENPRYINNPNRDSLQNQGVFGSRDLNFSEFGSV
DRATAGRNGNTLYPKPESGRILDFSESKRSSTNNAALFSGQSQFLGAEENNNINNKNKSK
KKRSPGSCGSNEDGMLSFVSGVLPSSSMGKSDGGAFTGADPDHSDLDASIIREVESIRAV
EPEKKPRKRGRKPANGREEPLNHVEAERQRREKLNQRFYALRAVVPNVSKMDKASLLGDA
ILYINELKSKLDNTECDKEELRNQIEELKRELLSKESRHSSSSAISLPEDMKTSTTTHQP
PMNLDVDVKVIGWDAMIRVQCNKKNHPAARLMAALKDLDFEVNHASVSVVNDLMIQQATV
KMGGRLYTQDQLRLALTNRFSDPL
>FGENESH:[mRNA] 13 3 exon (s) 62470 - 64881 378 bp, chain -
ATGGTTCGAGATTACCTGCCGAAAAAGGACTCGCCGGAGCTCGCCGGAGACGCGAACCAG
TCGCCGGAAACTCCTTGTGAGCGTCGGAGCTCACCACCGGGGCTCTACCTCCCTCGAGCT
CCATCTGTTACTGAGGTAGAGTGCCTTAGACCCGGTACCACGCCTATCCGCCCTTTATTT
TCCATTCAAAAGATACCACTGCAACTTACGCTGATTATCATGCACTACTATACTGTAGCT
GCCTTAAAGCTCTCGCAGCTGAATTTAGCACTGAGCTCCATGAACGTACATCAGACTGCG
CTGGCAGCTCTCCCCCGCTTGGGATCCGAACTCGTACCTGTTTGCGACGGCAAACAATCT
TCACATCAACGCGCCTAA
>FGENESH: 13 3 exon (s) 62470 - 64881 125 aa, chain -
MVRDYLPKKDSPELAGDANQSPETPCERRSSPPGLYLPRAPSVTEVECLRPGTTPIRPLF
SIQKIPLQLTLIIMHYYTVAALKLSQLNLALSSMNVHQTALAALPRLGSELVPVCDGKQS
SHQRA
>FGENESH:[mRNA] 14 1 exon (s) 72833 - 73357 525 bp, chain +
ATGAGGAGGTATAACAAAAGTGGTTCATCAAATCCTAGAGGGTCTGAGTCTTCGTCACAA
CAAAACGTAGACAGCCCTCCGCAGCAAGAGGGATACCGTCCAACTTTCGATCCTGTGCAA
TATCAATCGCAGTTGGCATTTCAAGCTTGGATGCAAACACAACCACCGTATCAGTATCAA
TATCAACAACCACAGTATCAACAAGATGACGACCCTTACGACCTCGACCAAGATGACGAC
CTTCCCATGCCACCACCACGGACTTTGCCCATTGGTAGAGATAGAGCGAAGAAGAAATCA
AAGACGAGTAGTGATTCTTCCGCAAAATCGTCTTCTGCAGCATCGGAAGCCTTGGCTAAA

ATCGAAGCTCAGTTTGCCAACATGACTGCCGAACTCTCATCAAGGAACGAGCTTCGAGCT
ATCCAGATGTATATGAGAGATATTAGTCACCTTCAGGGTGAGGAACTTGAGAGGGCAAAT
GCGCTCAATGCCCATCTCAAGCAAAAGTACGGGTGGAACTTTTAG
>FGENESH: 14 1 exon (s) 72833 - 73357 174 aa, chain +
MRRYNKSGSSNPRGSESSSQQNVDSPPQQEGYRPTFDPVQYQSQLAFQAWMQTQPPYQYQ
YQQPQYQQDDDPYDLDQDDDLPMPPPRTLPIGRDRAKKKSKTSSDSSAKSSSAASEALAK
IEAQFANMTAELSSRNELRAIQMYMRDISHLQGEELERANALNAHLKQKYGWNF
>FGENESH:[mRNA] 15 1 exon (s) 75026 - 75250 225 bp, chain +
ATGGCAGGGCGAAACCAGTTGACACACGTATCAAGGCAAGAGCGTTACCGGGATAAGAAG
ATCATATATAAAGCCCGGGACGCAGCACAGACGAAGATCATTATGTCGGCTCAATCCGTA
ATCATGTCGCTAGCCTCTCTGCGTACTTGGCCCTTATCCGATTGCAAGTGCCCAGGCCCA
ATCCGAGACCCAATTGACGGCTTACTCGACCCTCAAGCCGGTTAA
>FGENESH: 15 1 exon (s) 75026 - 75250 74 aa, chain +
MAGRNQLTHVSRQERYRDKKIIYKARDAAQTKIIMSAQSVIMSLASLRTWPLSDCKCPGP
IRDPIDGLLDPQAG
>FGENESH:[mRNA] 16 1 exon (s) 78976 - 80832 1857 bp, chain +
ATGGCTCAAATTGAGTTTGGGGAAAAATGGAGGTTTTGGATAAAAGAGTGTCTTTCGTCT
TCTAGGGCCTCGGTGTTGATCAATGGTTCCCCTACTAGAGAGTTTCCTATCACCAAAGGA
GTTCGACAAGGAGATCCTCTTTCTCCATTCCTTTTTATCATCGCCATGGAAGGGCTTAAC
GTGGCGATGAAATCCGCGTGTGAAAGGTCGATTTTTGAAGGTGTCTCGATCCCAAACGGG
GGTCCGTCTATTTCTCACCTTTTTTATGCGGACGATGCCCTTTTTGTGGGACGGTGGTCG
AGTTCAAACTTTGCAAACCTCGCGAGAATTCTGAAGTGTTTTCATGCTGTTTCGGGTTTG
AAAGTAAACTTTCATAAATCAAATGTGTTCGGCATTAGTGTGTCCAACAATGAGATCTTG
AGTTGTACTCGTATCCTTGGTTGCGAGGCGGGTTCTCTTCCCTTCAAGTATTTAGGGGTT
CCCGTGGGAGCAAATCTTTCGTTGAAACGACATTGGGATCCCATAATTGAAAGAGTGCAT
GGGCGTCTTTCGTCTTGGAAAGCCAAATCCCTTTCCTTTGGTGGCAGATTAACTCTCGTG
AAGTCGGTGCTTGGTAGTCTTCCTTTGTATTTCTTCTCCTTGTTTAGAGCCCCGAAGTCG
GTAATTGATACTATCGAAAAATTGCGAAGAAGATTCTTATGGGGAGGAAACGAGGAAAAA
AACAAGATAAATTGGGTTGCTTGGAAAGTGATTTTGGGGAATAAAGGTGACGGCGGGCTT
GGTGTCGGATCTCTCAGTTCCTTAAATTGGGCTTTGCTCGTGAAGTGGGTCTGGAGGTTC
AAAGCGGAGCCGATGTCTCTATGGCGTCGGGAAATCTGCGGCATTCATAATGCACATCGA
AAACATCTCAAGTCGTTAGCCAAAAAATCGTTCCCGGGAGTTTGGTTAAAAATATCGAGT
GTCATTAAAGACCTGGAACTAATTGGGATTGATATCCATTCCATTTTAAATGTGCAGATT
AATTCAGGTGACGGGGCCTTATTCTGGTACGACAATTGGACGGGTGAGGGAAGTCTTGCC
TCAAAGCTGGGTTCTATTTTCGAGCTGGACAAAAGAAAATCTTGTTTGGTTGCTGATCGT
GTTGATGGGAACGGCTTTAAAGAAGATTGGAAGAGGATGCCGAGAACCCGGGAGGAGAAT
GAAGAATTGGCTTCCTTGCGGAGATGGGTGGAGCCTTTCGCCGCTGGCGATGGGGAGGAC
AGATGGCGTTCCTCCTTATCCTCTGATGGTATTTATTACGTGCGGGACCTTCGGGGACTT
ATCGATTCAGTGCTGACTGAATCGGCTGCCAACCCCACTGTTTGGATTAACATTGCTCCT
CTTAAAGTTGTGTGCTTCATTTGGAGGGCGTGCTTGAACAGAATCCCGACGGCTGTGGCT
CTTGCGCGGAGAAAAATCCCCATCGCAAATGCCTCCTGTCAAAGCTGCACAGAGGGAGCA
GACGAAACTGATCATATTCTCGTGGACTGCCCGTTAGTCAAGGACGTACTGCATAAAATC
TTTAATTGGTGTAGGATCCCGATTCAACCGTTCAACACTGTCAAGGACCTGGTTACTTTT
GCTGCACAGTGGGGAAATTGCCCGAAAAAGAGGAAGGTTTTCTTAGCAATTATATATGGT
TTCCTTTGGAATGCTTGGCGGGCTCGCAATGACAAGATTTTTAATAAAATCTGCACCTCC
AGCTCGAAAATGGGGGATAATATTATCACAATGGTGTTTAATTGGGTGAAGTATAGGAGT
AATTTTGCCACTTTTAATTGGAGCGATTGGTGTATAGACCCTCTAAAAACACTGTAA
>FGENESH: 16 1 exon (s) 78976 - 80832 618 aa, chain +

MAQIEFGEKWRFWIKECLSSSRASVLINGSPTREFPITKGVRQGDPLSPFLFIIAMEGLN
VAMKSACERSIFEGVSIPNGGPSISHLFYADDALFVGRWSSSNFANLARILKCFHAVSGL
KVNFHKSNVFGISVSNNEILSCTRILGCEAGSLPFKYLGVPVGANLSLKRHWDPIIERVH
GRLSSWKAKSLSFGGRLTLVKSVLGSLPLYFFSLFRAPKSVIDTIEKLRRRFLWGGNEEK
NKINWVAWKVILGNKGDGGLGVGSLSSLNWALLVKWVWRFKAEPMSLWRREICGIHNAHR
KHLKSLAKKSFPGVWLKISSVIKDLELIGIDIHSILNVQINSGDGALFWYDNWTGEGSLA
SKLGSIFELDKRKSCLVADRVDGNGFKEDWKRMPRTREENEELASLRRWVEPFAAGDGED
RWRSSLSSDGIYYVRDLRGLIDSVLTESAANPTVWINIAPLKVVCFIWRACLNRIPTAVA
LARRKIPIANASCQSCTEGADETDHILVDCPLVKDVLHKIFNWCRIPIQPFNTVKDLVTF
AAQWGNCPKKRKVFLAIIYGFLWNAWRARNDKIFNKICTSSSKMGDNIITMVFNWVKYRS
NFATFNWSDWCIDPLKTL
>FGENESH:[mRNA] 17 1 exon (s) 86750 - 87112 363 bp, chain -
ATGGGAATATTGAAAATGTTGATTAGTTCATTGTCCCAATACATCCTTAATGTGCAGTTT
GACTTATGGTTTTGGTTAAAAACATTCAACTTTGATAGTAGAATGCTAAAAAGGGGTCAT
TGGGGCAAGAATGGCTCAATTTCTAAGTTTATACTCGGTATTGCCCAGTGCGCGGCCGCA
CGAACGGTCTGCGCGGCCGCACAGTTGGTCAATATGCACAAAAGCTGGGTCTCTCGGTAC
GCGCCCACGCACATAGGCCGCGCGGCCGCGCGTTTGTCAGTTTTCACTATTCACGAGCTT
TTGCGTGGCTTGTCCGATTCCAACGATTCCAATTGCAGTAGTCTTAGATATGATGAAAAA
TAA
>FGENESH: 17 1 exon (s) 86750 - 87112 120 aa, chain -
MGILKMLISSLSQYILNVQFDLWFWLKTFNFDSRMLKRGHWGKNGSISKFILGIAQCAAA
RTVCAAAQLVNMHKSWVSRYAPTHIGRAAARLSVFTIHELLRGLSDSNDSNCSSLRYDEK
>FGENESH:[mRNA] 18 1 exon (s) 92375 - 92701 327 bp, chain -
ATGGCTGTTGCAAGCAATACAATAGCAAATGATGATGAACTTGATGGGAATGATGTTTTA
GAAGAAGATGAAATGGGAGAAGAAGATGATGAGGAAGTTTTTGACAAAAGCTTTGAAATA
CCATCGACATTTACTAATATGGAGGAAACAAATATGACTACCGATGGCAAGTGGGTTGTG
TCAAAAATTGTGAGCAAGAATGACTTTACACAAGAGTTAGGAAAAGATTCTTTTAAGGAT
AAAGAAGAAGTTATGAGAGCTATCAAACTCCACTGCATTCGGACACATAAACAGTTTGAG
ATCGTTGAGACACGTCCAGATATATGA
>FGENESH: 18 1 exon (s) 92375 - 92701 108 aa, chain -
MAVASNTIANDDELDGNDVLEEDEMGEEDDEEVFDKSFEIPSTFTNMEETNMTTDGKWVV
SKIVSKNDFTQELGKDSFKDKEEVMRAIKLHCIRTHKQFEIVETRPDI
>FGENESH:[mRNA] 19 1 exon (s) 97188 - 98009 822 bp, chain -
ATGACTTTAGTTATTAGATGTGTAAACTTGTCCTCTAACAAAGTTAAAATTGAAGAGTTT
TTTTTAGAATTTTTAAAAGTGGATGATACTTCTGGTTTGGGAATTTTTAATGTGTTATTA
GATGCAATTAAAACACTTGGTCTTAATATTGATGATGTAAGAGGACAAGGTTATGATAAT
GGGTCTAACATGAAAGGAAAACGTAAAGGGGTTCAAACACGAGTACTTGAAGTTAATCCA
AGAGCATTATATATGTCATGTGCTTGTCATAGTCTTAACCTTTCACTTAGTGATATGGCT
CATTCTTGTGTTAAAGCAACTTCATTTTTTGGAATCTTGCAACGTATATTTGTGTCATTC
TCTAGTTCTACTAAAAGATGGACAATATTACTTGATAACGTTCCTAATTTAACCGTAAAA
TCTGTATGTAATACACGTTGGGAAAGTCGAATAAAAAGTGTCAAAGCTATTAGGTTTCAA
GCTCCTCACATAAGAGATGCATTGTTAGAATTATATAAATCATGTGATGATGCAAAGTCA
AAAAGTGAAGCAGAAAGTTTAGCTAATTCTATAGAAAGTTATGAATTTTTACTTGGTATG
GTAATTTGGTATGATATCTTATATGCTATTAATAAAGTTAGTAAGAAGTTGCAAAACAAA
ACAATATGTATTGATGCCACCATTGATCTATTAAAACACATGATAACATTTTTTAAAGAG
TATAGAAATGAAGGTTTTGCCACTAGTGTAGATAATGCTAAAGACATTGCATTAGTTATG
GATGTCGAACCTATATTTCCAAAAAAAACGCCGAATTATTAG
>FGENESH: 19 1 exon (s) 97188 - 98009 273 aa, chain -

MTLVIRCVNLSSNKVKIEEFFLEFLKVDDTSGLGIFNVLLDAIKTLGLNIDDVRGQGYDN
GSNMKGKRKGVQTRVLEVNPRALYMSCACHSLNLSLSDMAHSCVKATSFFGILQRIFVSF
SSSTKRWTILLDNVPNLTVKSVCNTRWESRIKSVKAIRFQAPHIRDALLELYKSCDDAKS
KSEAESLANSIESYEFLLGMVIWYDILYAINKVSKKLQNKTICIDATIDLLKHMITFFKE
YRNEGFATSVDNAKDIALVMDVEPIFPKKTPNY
>FGENESH:[mRNA] 20 4 exon (s) 102433 - 108667 4653 bp, chain +
ATGGCCCGTACTAGAAGTGGGCAAGGAGGTGGACAGAGCGGAAGTCAAAATTCTCGGGGA
AGACCGAGAAGTGTGAACCGCCCTCCTCCAATTATGGAAGATGGAAATAGCAATCCCGCT
CCGGTAACTCTAGAAGCTATCCAGAAGCTTCTTCATGACCAGCAAGTGCAAAACCGGGCG
GAGATGAATGACCTCATTAACGAAAGATTACAAGAGTTTTCGAAGGCTCTAAACCATGAT
CCGGCATCCGGAAGTGTGCATCCTTCTCACTCCCCGACTCATGAAAGTCACCATACTGCT
CGGCATTCACCTTCTCCTCCACCTCAAAATCCCCCACCACTAAATCCTCCAGGGCCGCGA
GTTACCTTCAAAGATTTCATGATATGTCGCCCAAAAGAGTTCCATGGGGGCAAAGATCCT
AAAGTTACCATGTATTGGTTAGCCGAAATCGAGCAAGTACTTCGAGTTTGCCGATGTGAG
GAAGAGATGAAGGTGACTTTTGCCTCCCAAATGCTGAAGGGAGATGCCCTCACATGGTGG
AATACTCTCACCACCACCTTGGGTGGTCATGTCGTGGCTTCCTTCACTTGGGCGGAGTTC
GTTCGGAGGATCAAGTTGAAGTTTTGCTCTCCTTTGCATAAGGAGATGATAGTAAACGAG
TTCTTTGCGCTTAAAAAGGGCAACAGGTCGATTGATGAGTACTCGAAGAAGTTTACGGAT
ATGATTCCGTTTCTTGAAGATACTCTCCCTTTGGAAAGTGGAAGAATCAACCGGTTTGTG
GACGGGTTGCCCGGGGATTACTTGTTGGAAGTGAGCAAGGCCACAACTTTGGATGAGGCC
ATTGATTTGGCCGTAAAAGTGGAAAATATGTTAGCCCGAAAGGCTAAAGAAAGTGGTGGA
GCCGGTGAGAAGAGAAAGCAAGTGGAGGTTTCCAGTTCAAACAAGAAAACACGATTCTCC
CCAAAATCCGCAACAAAGGATGATCGACCAAAGTGTGACAAGTGCGGGAGAAAACACCGA
GGTGAATGCATGAAGGGAACCGAGACTTGCTATAGGTGTGGAAAGCCGGGGCACATGGCG
CGTGACTGTGATGTGAAAGATTTGTCCGGGGTCGAATGCTTCTCGTGTCATGAAATGGGA
CACTTTAGCAACAGATGCCCGAACAAAAAGGGAGAAGGATCCAGGACTATGGGTGAGAAG
AAAAAGGAAGCACCAAAGAAAGGAAGGGGTTTCCAAATGACATTGGCGGAGGCCAAGGAG
ACGGAGGACGTGGTGGCAGGTACTTTACTTGTCGATACTAATTTAGCACATGTTTTATTT
GACTCGGGTGCTAGTTACTCTTTTGTATCGTATGAGTTTTGTAAGAAGTTAGATGGGCCT
ATGGCTACTTTACGTGATCCCTACATGATCGAGGTTGCCGATGGGAGGGTTGTTCCCGTG
AAGCAACTTTATCCAAATTGTGATGTCGAGGTTGATGGGTGGCATTTTCCCATTGACTTG
TTATTAATCGGAATCAAAGGCTTCGACATCGTGGTGGGGATGGATTGGCTTAAAGCGTAC
GAGGCCAAGATTTTATGTGGAAGACGTATAGTGTCGATTAAAGTAGCTCCCGGAGAGAAG
ATTTATATATATGGGAAATGTAACAACAAATTCCCATGTGTAATATCGGCGGTCAAAGCT
CACAAAAGTATCCAAAAAGGGTGCGAAACATTTTTGGCTTTTGTGCTCGACGGTAGAAAA
GAGAAGACATGCGCGAAAGTGAATGATGTGAGAGTGGTGAAGGAATTTCCGGAGGTATTT
CCCGAGGACTTGTCCGGATTACCACCGATTAGACAAGTGGAATTTGCCATTGAACTAGTT
CCGGGTGCAAGACCGATCGCGCGGGCGCCTTACCGTCTTGCTCCTACCGAGATGAGGGAA
CTTATGGCACAACTCCAAGAACTCCTCGACAAGGGGTTTATTCGTCCGAGTTCGTCACCT
TGGGGTGCACCGGTATTGTTTGTGAAGAAGAAAGATGGCACAATGCGAATGTGCATCGAT
TACCGGGAACTAAACAAGGTGACCGTCAAGAATCGTTATCCGTTGCCGCGGATAGACGAT
TTGTTTGATCAATTGCAAGGAGCGGATTATTTTTCGAAGATTGATCTTCGTTCAGGCTAT
CACCAAGTTCGGGTGAAAGACGAAGATGTTTGCAAAACCGCGTTCCGAACCCGATACGGT
CACTACGAATTTCTCGTAATGCCGTTTGGTCTCACGAATGCTCCAGCTATATTCATGGAC
TTAATGAATCGAGTGTGCAAGCCATTCCTAGATAAGTTCGTTATCGTTTTTATTGACGAT
ATTCTTATCTACTCAAAGACCGAAGAGGAGCATGAAACTCATTTACGTGCGGTTTTACAA
GTGTTGAAGAACGAGGAACTTTATGCCAAATTCTCGAAATGTGAATTTTGGTTGCGAGAA
GTGCAATTTCTCGGTCATATTATTTCTCGTGAAGGCGTGATGGTAGATCCCGCGAAGATC

GAGACGGTGATGAAGTGGGAACCCCCGAAAACTCCCTCGGAAGTGAGAAGTTTCCTTGGG
TTGGCCGGGTACTATCGGAGATTTATTGAAGGCTTTTCTAAAATTGCGGTTCCTCTTACT
CAACTTACCAAGAAAGAAACAAAATTTGAGTGGAACGAAAAACGTCAAGAAGCCTTCGAG
ACCTTGAGAAAGAAGTTGTGCGAAGCACCGGTGTTGACTTTACCGGAGGGCACGGAAGAC
TTTGTGGTCTTTAGCGATGCATCGAGCCATGGGCTCGGGTGTGTGTTGATGCAGAGGGGG
AAAGTAATTGCCTATGCTTCGAGGCAATTGAAAGATGCGGAAACTCGATACCCGACACAT
GACTTAGAATTGGCGGCTATAGTTCATGCGTTGAAGATATGGCGACATTACTTGTACGGG
ACAAAGTGCAAGTTGTACACCGATCACAAGAGTCTCCAATACGTCTTTACGCAAAAAGAC
TTAAACATGAGGCAACGAAGGTGGATCGAGCTTATCAATGATTATGATTGCGAAATCATA
TATCATGAAGGAAAAGCTAATGTTGTAGCCGATGCTTTGAGTCGGAAGATACGTGAAAAG
CCCATTCGAGGTAGATCGATGCGAATGGAGTTGGTGTCGACTCTTGTGCAATTGATAAAA
GAAGCACAAACGGAAGCTTTGAAAGTTGAAAATGTTAAAAACGAGAAACTCGGGAAAAAG
ATCGAGTTTGAAGAAAATGCACGGGGTTTGAAAACCGTGAGAAGACGGGTTTGGGTACCG
AAATTTGGTGGAAACCGGAAGATGATCTTGGACGATGCGCATAAATCCAAGTATTCTATT
CATCCCGGTAGTACCAAAATGTACCGAGACTTGAGGAGACACTATTGGTGGCCCGGAATG
AAGCCGCGGGTAGCAAAGTTTGTCTCTAAGTGTGCTACATGTGCACAGGTGAAGGCGGAA
CACCAAGTTCCTTTTGGGAATATACAGTCACTCCAAGTCCCGATTGGAAAATGGGAAGAT
ATTACCATGGATTTTGTCATGGGTCTTCCAAGGACGAGCAAGGGCAACGATGCAATTTGG
GTGATAGTTGATCGCCTTACAAAAAGTGCCCTTTTTATTGCCATGAAGGAGACGACTCCG
TTGGAAAAGTTGGCAAAGTTGTATGTTGATGAGGTGGTGAGGCTACATGGAGTCCCGTTG
ACTATTGTATCGGACCGAGATGCTAGATTCACATCTAGATTTTGGCAAGCGTTACAAAAA
GAATTGGGGACGAAAGTGCTTTTGAGCACAGCCTACCATCCTCAAACTGATGGTCAAAGC
GAACGAACCATCCAAACCTTAGAAGATATGCTAAGGTCATGTGTGATTGACTTTGGTGGG
AGGTGGGACGAATTTCTACCTCTGGTGGAATTTTCGTATAACAATAGTTATCATTCGACT
ATTGAAATGCCACCATTTGAAGCCTTGTATGGAAGGAAGTGTAGAACTCCCGTGGGCGAT
TTAGTGATGTTGAAAGTGTCACCATGGAAAGGAGTGATGAGATTCGGTAAGAAGGGCAAG
TTGAGTCCTCGGTATGTGGGTCCTTTCAAAGTGATAAAAGTGGTGGGTACGCAAGCTTTT
AAATTGGAGTTACCGCAAGAACTCCAAGGGATACATGATACGTTTCATGTAAGCTACTTG
AAGAAGTACTTTGGAAAAGAAGAATTGACAATTCCATTGAAGGAATTGAAAGTTGATGAG
AAGAAACGGTTGATTGAAGAACCGGAAGCTATCTTGGAATGCAAGACCAAGAAGCTAAGG
AACAAGGAGATTGATTTGGTATTAGTGAGATGGAAACATTCACTTGGACCAAACTTGACT
TGGGAAACCAAGGATGAGATGATGAAGAGGGGCCAAAGTGTAAAGTTGGCCAAACTTGAG
GGGCTAAAGGGGATATGCTGGAAGATTAAATTGGCTGGACATTTCTTCCAGAAATGTGTT
CGTAAGGTGAGAAGGAAGAAGAAGAAAAGGAGGGTTTTTCATCCAAGAGCTAGAGTGATC
AAATCCAAGTGGTTAGAAGGTTACAAAGGAGAAAAGTTAGTAGAAGGAGGATCTGCAGGA
GCAGCAAACGATCCCAAAGCCTCTACAAGTTGA
>FGENESH: 20 4 exon (s) 102433 - 108667 1550 aa, chain +
MARTRSGQGGGQSGSQNSRGRPRSVNRPPPIMEDGNSNPAPVTLEAIQKLLHDQQVQNRA
EMNDLINERLQEFSKALNHDPASGSVHPSHSPTHESHHTARHSPSPPPQNPPPLNPPGPR
VTFKDFMICRPKEFHGGKDPKVTMYWLAEIEQVLRVCRCEEEMKVTFASQMLKGDALTWW
NTLTTTLGGHVVASFTWAEFVRRIKLKFCSPLHKEMIVNEFFALKKGNRSIDEYSKKFTD
MIPFLEDTLPLESGRINRFVDGLPGDYLLEVSKATTLDEAIDLAVKVENMLARKAKESGG
AGEKRKQVEVSSSNKKTRFSPKSATKDDRPKCDKCGRKHRGECMKGTETCYRCGKPGHMA
RDCDVKDLSGVECFSCHEMGHFSNRCPNKKGEGSRTMGEKKKEAPKKGRGFQMTLAEAKE
TEDVVAGTLLVDTNLAHVLFDSGASYSFVSYEFCKKLDGPMATLRDPYMIEVADGRVVPV
KQLYPNCDVEVDGWHFPIDLLLIGIKGFDIVVGMDWLKAYEAKILCGRRIVSIKVAPGEK
IYIYGKCNNKFPCVISAVKAHKSIQKGCETFLAFVLDGRKEKTCAKVNDVRVVKEFPEVF
PEDLSGLPPIRQVEFAIELVPGARPIARAPYRLAPTEMRELMAQLQELLDKGFIRPSSSP

WGAPVLFVKKKDGTMRMCIDYRELNKVTVKNRYPLPRIDDLFDQLQGADYFSKIDLRSGY
HQVRVKDEDVCKTAFRTRYGHYEFLVMPFGLTNAPAIFMDLMNRVCKPFLDKFVIVFIDD
ILIYSKTEEEHETHLRAVLQVLKNEELYAKFSKCEFWLREVQFLGHIISREGVMVDPAKI
ETVMKWEPPKTPSEVRSFLGLAGYYRRFIEGFSKIAVPLTQLTKKETKFEWNEKRQEAFE
TLRKKLCEAPVLTLPEGTEDFVVFSDASSHGLGCVLMQRGKVIAYASRQLKDAETRYPTH
DLELAAIVHALKIWRHYLYGTKCKLYTDHKSLQYVFTQKDLNMRQRRWIELINDYDCEII
YHEGKANVVADALSRKIREKPIRGRSMRMELVSTLVQLIKEAQTEALKVENVKNEKLGKK
IEFEENARGLKTVRRRVWVPKFGGNRKMILDDAHKSKYSIHPGSTKMYRDLRRHYWWPGM
KPRVAKFVSKCATCAQVKAEHQVPFGNIQSLQVPIGKWEDITMDFVMGLPRTSKGNDAIW
VIVDRLTKSALFIAMKETTPLEKLAKLYVDEVVRLHGVPLTIVSDRDARFTSRFWQALQK
ELGTKVLLSTAYHPQTDGQSERTIQTLEDMLRSCVIDFGGRWDEFLPLVEFSYNNSYHST
IEMPPFEALYGRKCRTPVGDLVMLKVSPWKGVMRFGKKGKLSPRYVGPFKVIKVVGTQAF
KLELPQELQGIHDTFHVSYLKKYFGKEELTIPLKELKVDEKKRLIEEPEAILECKTKKLR
NKEIDLVLVRWKHSLGPNLTWETKDEMMKRGQSVKLAKLEGLKGICWKIKLAGHFFQKCV
RKVRRKKKKRRVFHPRARVIKSKWLEGYKGEKLVEGGSAGAANDPKASTS
>FGENESH:[mRNA] 21 7 exon (s) 111529 - 114501 2031 bp, chain +
ATGGCTGATTCTGTCGATACTTTGGCTTCGCCCTCTGAATCAGTTACTAAATACTCAGAT
ACGGTTGAATCGTTTTGGCCAAAGGATTCTCGTTCGCCTACACAAACCCTAGATCGACAG
TCGAGCTCTCCGGATACTAAGGTCGATGCTCACGTTAGCGTGTTTGATCAATTATTACGC
GAAGAGGAGGAAAAATTGGAGGATTTTAAGACAACAGAGTCGCAAGTGAATCCTATTATG
TCCGCTGAAGCGCTCGAAGATTCTGAAGAACCGGCGCTATCGCTCTCGGGTGCGGAAGCT
AATGCCCCAACTGAGCCCTCGAGTCCAGTGGATGTCGATGATAACGCGAATAGTCTCGCC
GATGAGACTGATGCGTGGATTTCGTGTAATCGAAGTGAAAACGATGTTTACAAACCGATG
CCCTCAGTTCCATCAAGTTCTTCAAGTTGGTACGAAGATGCCTACGAGAAACCGTATATG
GTGGGTGCAGGGCTTGCTAATCTAGGAAACACATGCTTTTTCAATGCAGTTATGCAGTGC
TTCACACACTCTGTGCTACTTGTTCAGGGTCTATACTCGTACACCTTTCCAACACCTTGT
GATTATTTCTCATCATCTTTTCAAAGATATCAGCAAGAAGATGCTCATGAATTCCTGCAA
TGCTTTCTTGATAGACTTGAAACTTCCTTCACCAATTTAAAACTGAAAGATGTTGCTTTT
TCCTCACAAAGCAACAATCTAGTCAAACAAGTATTTGGAGGTCGTGTCATCAGCAAATTA
AGATGTTGCAACTGTAACCACATCTCTGATACATATGAGCCATCAGTTGACCTAAGTTTG
GAAATAGATGATGCAAATACTCTTTCAACTGCCCTTGAGTCATTCACAAAAGTCGAACAC
ATTGGAGATGAAGAAATGAAGTTCACATGTGACAACTGTAAAGAAAAAGTCTCAGTTGAC
AAACAACTTATGCTGGATCAAACTCCTCCCATCTGTGCTTTTCACTTAAAAAGATTCAAA
AACGATGGCTCTTATGTTGAAAAAATAGACAAACATGTTGAGTTTCCTCTTGAGTTGGAC
TTGCAGCCTTACACATGTGGCAATCCAACCAATAACAACGAGGATTTGAAATATGAACTA
TATGCAGTTGTTGTACATGCAGCCTATACATCCACCTGTGGTCATTACTATTGCTACATT
CGATCTGCACCTGAAACATGGTACAAGTTTGATGACTCAAAGGTTACTAGTGTGTCTGAA
TCATATGTGTTATCAGAAGAAGCCTACATCCTATTCTATGCAAAACAAAATACACCATGG
TTTTCAAACTTCATGGAAACATATAAACCTTCATTAGACCCAAATCTTTCAAACACATCC
CCAAAATCCGTACTTGAAAATCTTGATGCCCAAAACATCGAATCTTGTTCAACATCAACA
CATCCCAAAGTTCCTGAATCCAAACATCCATGTTCCAAATCAGATGCTGTGGTCCCAAAC
ACTCCAATTCATGTTAAAACAGTAACCCCCGTTTTAACCCTCAAAGAATACGGGTCCAAT
CACAAAGTTGTCACTCCTACAAACCACACCAACATCCAAACCGATAATTACGAAAATGAC
CCTATCAGAAGAAAAACACCATTTGAAATCGAAGACATTCTTTCTCCATCCACCCCTCAA
AGAACCCCTGGGTTTTACTCCTCTGATGATGATGAAACTCCAGAAGTGGTATTTGATGCA
AAACCTGAGAAACCAAAGGATGTGGAAAAACAATTATTGTCAGCATATAAGAGACCAAGA
AACAAGGAAAGGGAGGAAATTGCGAAACTTGAGGCATTAAAACAATGTAGTAAGAGATTC
AAAGGTTCTAGAAAAAACCAGTTAATGGCGGCGATAAAGAGCGATAGTTCACTTTACAAA

AGAAGCAAGAAGATGACGTCATCATCACCTTCTAGAAACAGAAAGAGTTTAAGTAGGTCA
AGTGACAGGAACCCAATGCCTCGTAATTTGGCCACTTCGAGTTTTCGTTGA
>FGENESH: 21 7 exon (s) 111529 - 114501 676 aa, chain +
MADSVDTLASPSESVTKYSDTVESFWPKDSRSPTQTLDRQSSSPDTKVDAHVSVFDQLLR
EEEEKLEDFKTTESQVNPIMSAEALEDSEEPALSLSGAEANAPTEPSSPVDVDDNANSLA
DETDAWISCNRSENDVYKPMPSVPSSSSSWYEDAYEKPYMVGAGLANLGNTCFFNAVMQC
FTHSVLLVQGLYSYTFPTPCDYFSSSFQRYQQEDAHEFLQCFLDRLETSFTNLKLKDVAF
SSQSNNLVKQVFGGRVISKLRCCNCNHISDTYEPSVDLSLEIDDANTLSTALESFTKVEH
IGDEEMKFTCDNCKEKVSVDKQLMLDQTPPICAFHLKRFKNDGSYVEKIDKHVEFPLELD
LQPYTCGNPTNNNEDLKYELYAVVVHAAYTSTCGHYYCYIRSAPETWYKFDDSKVTSVSE
SYVLSEEAYILFYAKQNTPWFSNFMETYKPSLDPNLSNTSPKSVLENLDAQNIESCSTST
HPKVPESKHPCSKSDAVVPNTPIHVKTVTPVLTLKEYGSNHKVVTPTNHTNIQTDNYEND
PIRRKTPFEIEDILSPSTPQRTPGFYSSDDDETPEVVFDAKPEKPKDVEKQLLSAYKRPR
NKEREEIAKLEALKQCSKRFKGSRKNQLMAAIKSDSSLYKRSKKMTSSSPSRNRKSLSRS
SDRNPMPRNLATSSFR
>FGENESH:[mRNA] 22 1 exon (s) 116223 - 116960 738 bp, chain +
ATGATTCATCACAAAGTTTCGAGTCTTGTTTTCCTGGTGTTACTAGGTTTGGGCATTTGT
GCCGCGACTAGAGTTCTCTTGACTATTGAGGAGAGCATTCCTTACACTCATGGTGGTTTA
TCAGGAGGTGGTGGTGGTGGAGGGGGTAGTGGAGGCGGAGGTGGTGGTGCATATGGGGAA
GAAGTAGGACATGGAGGTGGATACGGAGGTGGAGCTGGTAAAGGTGGTGGTGAAGGGTAT
GGTGGTGGAGCGGCCGGAGGTGGAGGTGGAGGCGGAGGTGTTGGAGGAGGTGGTGGTGGT
GGAGGAGGAGAGGCAGGAGCAGGTTACGGTGGAGGAGAAGGAGGTGGGAGTGGTGGCGGA
CATGGTGGTGGAGCACTCGGAGGTGGAGGTGGAGGTGGTGGTACCGGAGGAGGTGGTGGT
GGAGGAGGTGGAGCACACGGTGGGGGTTACGGCGGAGGAGAAGGAGGTGGGAAAGGTGGT
GGATACGGTGGAGCTGGAGGAGCTGGTGGTGGAGGTGGAGGTGGAGGTGGTCATGGTGGT
GGTGGAGGAGGAGCTGGAGGCGATGGACATGGCAGTGGAGGAGGAGCTGGAGGGGGTGCC
GGGGGATCATATGGTGGATACGGTGGTGGAGGTGGTTCAGGAGGCGGAGGTGGAGGAGGT
GGTGGTGCAGGAGCGGGTGGATATGGAGGTGGGAGTGGGGGTGGTGAAGGTGGTGGTCAT
GGTGGCTATATCCCCTAA
>FGENESH: 22 1 exon (s) 116223 - 116960 245 aa, chain +
MIHHKVSSLVFLVLLGLGICAATRVLLTIEESIPYTHGGLSGGGGGGGGSGGGGGGAYGE
EVGHGGGYGGGAGKGGGEGYGGGAAGGGGGGGGVGGGGGGGGGEAGAGYGGGEGGGSGGG
HGGGALGGGGGGGGTGGGGGGGGGAHGGGYGGGEGGGKGGGYGGAGGAGGGGGGGGGHGG
GGGGAGGDGHGSGGGAGGGAGGSYGGYGGGGGSGGGGGGGGGAGAGGYGGGSGGGEGGGH
GGYIP
>FGENESH:[mRNA] 23 1 exon (s) 118656 - 119909 1254 bp, chain -
ATGCATTCACTTTCACTCATCATGCATGTTCACAAAGTTTCAAGTGTTGTTTTCTTGGTG
TTACTAGGTTTGGGCATTTGTGCCGCGAGCAGAGCTCTCTTGACTCTTGAAGAGAGCATT
CCTTACGGTCATGGTGGTTTAGCTGGAGGTGGAGGTGGTGGAGGGGGTAGTGGTGGCGGA
GGTGGTGGTGCATATGGGGAAGAAGGAGGACATGGTGCTGGATACGGAGGTGGCGCTGGG
AAAGGTGGTGGTTCGGGATATGGTGGTGGAGTGGCAGGAGGTGGAGGTGGAGGTGGTGGT
AGTGGTAGTGGAGGCGGTGGTGGAGGAGGAGCTGGAGGAGCCAAAGGTGGGGGTTATGGT
GGAGGAGAAGGAGGTGGGAGCGGAGGTGGATACGGTGGTGGTGCAGCTGGTCATGGTGGA
GCAGCCGGAGGAGGTGGAGGTGGTGGTAGTGGCGGAGGCCATGGAGGAGGAGAAGGAGCA
CAGGGTGGGGGTTACGGTGGAGGAGAAGGAGGTGGGAGCGGAGGTGGATACGGTGGTGCA
GCTGGTCATGGTGGTGGAGAAGCCGGAGGAGGTGGAGGTGGTGGTAGTGGCAGAGGCGGA
GGCGGTGGAGGAGGAGAAGCAGCACAAGGTGGGGGTTATGGTGGAGGAGAAGGAGGTGGG
AGCGGAGGTGGATACGGTGGTGCAGCTGGTCATGGTGGTGGAGAAGCCGGAGGAGGTGGA

GGTGGTGGTAGTGGCAGAGGCGGAGGCGGTGGAGGAGGAGAAGCAGCACAAGGTGGGGGT
TATGGTGGAGGAGAAGGAGGTGGCAGTGGAGGTGGATACGGTGGTGCAGCTGGTCATGGT
GGTGGAGCAGCAGGAGGAGGTGGAGGTGGTGGTAGTGGCGAAGGCGGAGGCGGTGGAGGA
GGAGAAGCAGCACACGGTGGGGGTTATGGTGGAGGAAAAGGAGGTGGGAGCGGTGGTGGA
TATGGTGGTGCTGGTGGAGCTGGTGGTGGAGGTGGAGGTGGAGGTGGACATGGTGGTGGT
GGAGGAGGAGGTGGTGGAATAGGAGGAGCAGGAGGAGGCGGATATGGTAGTGGTGGAGGC
GAAGGAGGAGGCGCTGGAGGATCCTACGGTGGACACGCTGGAGGAGGTGGTGGTGGTTCA
GGAGGAGGAGGTGGAGGTGGTGCGGGTAGTGCAGGAGCTGAGCTTGGAGCAGGTGGTTAT
GGTGGTGGCAGTGGGAGTGGTGAAGGAGGTGGTCATGGTGGCTATATCCCCTGA
>FGENESH: 23 1 exon (s) 118656 - 119909 417 aa, chain -
MHSLSLIMHVHKVSSVVFLVLLGLGICAASRALLTLEESIPYGHGGLAGGGGGGGGSGGG
GGGAYGEEGGHGAGYGGGAGKGGGSGYGGGVAGGGGGGGGGSGSGGGGGGGAGGAKGGGYG
GGEGGGSGGGYGGGAAGHGGAAGGGGGGGGSGGGHGGGEGAQGGGYGGGEGGGSGGGYGGA
AGHGGGEAGGGGGGGGSGRGGGGGGGEAAQGGGYGGGEGGGSGGGYGGAAGHGGGEAGGGG
GGGSGRGGGGGGGEAAQGGGYGGGEGGGSGGGYGGAAGHGGGAAGGGGGGGGSGEGGGGGG
GEAAHGGGYGGGKGGGSGGGYGGAGGAGGGGGGGGGHGGGGGGGGGIGGAGGGGYGSGGG
EGGGAGGSYGGHAGGGGGGGSGGGGGGGAGSAGAELGAGGYGGGSGSGEGGGHGGYIP
>FGENESH:[mRNA] 24 1 exon (s) 125889 - 125999 111 bp, chain +
ATGGATAAAAATGGTAAAACGCGGACAAAATGGCTTAAACGCGGAATGGTTTTGCCGAAC
GCGGACAAATCAGAATTGAACGCGGACAGAACCCTTCTGTCCGCGTTTTGA
>FGENESH: 24 1 exon (s) 125889 - 125999 36 aa, chain +
MDKNGKTRTKWLKRGMVLPNADKSELNADRTLLSAF
>FGENESH:[mRNA] 25 3 exon (s) 127237 - 129764 336 bp, chain +
ATGTCTATCACAGAGAAAGGGCTTCAACAGTTCTTGCTACAGCTTCAGCATCCTTTAAGA
ACTAAGGAGAAAGGTAATGGTACATCTCCTAGAAGTGGAAGGAAAATTGAGAAAATGGGG
AAGGTAAGGGCAGGTTCGGTTCAGGCTGGTTTGGACGAGATAGCTCACATCGGTGAGTGC
AAGGAGGTGTTCAAGCCACCCGCCATGGCTGCCCCGGTTTACACCACCACCCTACATCGC
CGGTTGACCGGACCATGCTCCGCCGCCACTGACCACCACTCCGGGATGGCAGTAGGTTTG
GCATCCTCTTCCAAATGCACGGTTAATCAGCCATAG
>FGENESH: 25 3 exon (s) 127237 - 129764 111 aa, chain +
MSITEKGLQQFLLQLQHPLRTKEKGNGTSPRSGRKIEKMGKVRAGSVQAGLDEIAHIGEC
KEVFKPPAMAAPVYTTTLHRRLTGPCSAATDHHSGMAVGLASSSKCTVNQP
>FGENESH:[mRNA] 26 5 exon (s) 137634 - 139928 1317 bp, chain -
ATGGCTGCTTCTGTGGAAAATATACTATGGAGAAGTTCATTGCTATTGTGGGTTGAAATG
GAACCGGATATGGAGAAAGAGTCTAAAAAGAGTCGAAGGCTGTTCCTCGATTACTGGCTT
GATGATGTGCTGCAAGGAAGAATTAAGGCTAAAACAGATGTTTGCCCGTTGACCTTTTCG
TTGACTAACCATAAAGACCAAAATAGCTCTAGAAGATGGCTGTTCCTCTTCTTGTGGTGT
GATGATCCAGTGCAACAATTTGTATTGACAGATAGCGACCGTTCAAAAAGGCCTACTCTC
TTCTTCCGTGTTCGTCCGTCTTTTCCTTACTCTCTTCTTCCCGCTCGGCCGTTCAAGAAC
AGGAGCCCTATCGCGACACTTCTCTGCTGCTCCGGTGCTCTGCTGCTCCGCTTCTCTGCT
GCTCGTCTGCTTCTCTGCTGCTCCGCTTCTCTGCTCGCGATCGGGGACATGGATTTTAAA
GTTGGACAACCAATCGAAATTATAGACGATGGATCAGGATTCCGTGCATCTTACTATACG
GGGACGGTTGTGCAAGTCTATGACGATTATAATGAAAGGGTAGCGGTAAGATATGATAAG
GTGAAAGATTATAATGGCAACCCTCTAGTTGACGATTTACCCAAGGAGGATTTGCGTCCA
GTTGCACCAAAGGTTGATGTCAATCTTGAAACAAATGACACTGTAAACGCTTGGGACGGA
GAAGGGTGGTGGAATGGAAAACTTATACAAACAGCAAGTAATTTGTACACAGTTGATCTT
GGTTCTGAATCTGATCCAAAGATAGAAGTCTTTAGAAAGAAGCATCTACGCATAAACCAA
CGTTGGCAGATGTTGGAACACGGACAACACCGTTGGGTTTATGTAAAAAACCAGAATGTG

GATTTTGCGTTTGGAAGTCGTGTTGAAATAGTCGGTTTTGAAGCTGGGATGACAAATTCT
TACTATGCTGGAACTGTTGTTGGATATAGTAACTATGCAAGGGTGCAGGTGAAGTATGAA
ACATTGACAGCAAATGGTAACGCACGTATTGAGGATTTCACGCGATCAGACATTCGTCCA
TATCCAGCAGATGTGAATGTAAACATCGAAGTTGGAGACATTGTTGATGTTTGGGAAAAT
CATGGGTGGTGGATGGGAAAATGCACAACTGTTAATACTCGCACAGATGAGTACACTGTG
GTCAAGGAAATTGATAAGAAGAGTAGGAAGTATGAAAGAGACGATGTGCGAATCAACCAA
CACTGGTTCCCTGCCGGTGCTTCTAGGTTTTGGTCTTATACTAACAATCTGTATTGA
>FGENESH: 26 5 exon (s) 137634 - 139928 438 aa, chain -
MAASVENILWRSSLLLWVEMEPDMEKESKKSRRLFLDYWLDDVLQGRIKAKTDVCPLTFS
LTNHKDQNSSRRWLFLFLWCDDPVQQFVLTDSDRSKRPTLFFRVRPSFPYSLLPARPFKN
RSPIATLLCCSGALLLRFSAARLLLCCSASLLAIGDMDFKVGQPIEIIDDGSGFRASYYT
GTVVQVYDDYNERVAVRYDKVKDYNGNPLVDDLPKEDLRPVAPKVDVNLETNDTVNAWDG
EGWWNGKLIQTASNLYTVDLGSESDPKIEVFRKKHLRINQRWQMLEHGQHRWVYVKNQNV
DFAFGSRVEIVGFEAGMTNSYYAGTVVGYSNYARVQVKYETLTANGNARIEDFTRSDIRP
YPADVNVNIEVGDIVDVWENHGWWMGKCTTVNTRTDEYTVVKEIDKKSRKYERDDVRINQ
HWFPAGASRFWSYTNNLY
>FGENESH:[mRNA] 27 10 exon (s) 142319 - 145353 1872 bp, chain +
ATGGAGAACAACAACCTCCTACCTGATGATGTGATGTATGGTGATGATGCACATTTAGTG
TATTGTACAGACGAAGATGATGGGGATGACAGCAGCTCAGAGCTTGAAGATGGGGATGAC
ATCAACGAAGAGCTTGAAGATGTGGAAGACAACAATATACACCCTGAAGTTGATGAAGTT
GAGCCAGGAATTGTTGCAGCCGCTGATATTGGATCAGATGCCGAGTCTGACTTCAATGAC
TCTGATCAGGATAACCAACTCCCCCGTGCTCAAAGACCAAACAGGAAAAGAGGCATGACA
CGATTGCCAAGACTACAGACAGATTACACCAGATCCGGTGGGAGGAAGAAGCTTGTGAAG
TTTGACAATTTGGGTAGGTTTTCAAGAAAGTATAGGGCATTGTTTGTGAGCTACTTGGGT
GATCTTGTACGACAGAAGCACTTTTTTGATGTTGACGACTGTCAATTTAAAGTTGTGATG
AAAAGGATGGGTATGCTTCTACGCAACTTCAGAAGAAAGGTTGCAAATAATGTGATTGCA
AAAGATGAGGAGCCGTCAAGAACATTATTGTGGTGTAAAGGTCGTGAAGATAAACATGGG
GACATTAAGCCTGAAGCTAAGATGATTGCTGATCAACTAATGGAACATGAAAAGCAGATT
AAGGATGGAACTGTACTCCTTGAACCTGGTACTGATGCCATGACCATGGTTTTTGGCAAG
GATAAGGGTGGCTATTTAAAGGGTGTGGGAACGGAAGTGACTGCAACCACATACTTTCAC
ATCCCTCGCAACAAAGGATCTGCTAAGGAGGAAATTAAGGAGCTAAAATGTGCAGTCCAG
AATGGGAAGGTTGAACTTGAGAAGAAAGATGCTCAAGTGAAGGCTTTGACTACAAAGTTT
GATGAACAACAAGAAACACTGAACTGGGTTTTAGCTCATCTTGCTGCTACAGGAACGAAA
ATTCCCAATCTCCCTAACACATCTGGTGTTTCACTAGACAATGTGGTTGAATCTCCTGCA
ACTGCTGACCTCAACGTATCCACAGAACCAACAGAGGAAGCCTTTACACAGGCAATTAAA
AGAACAAATAAAAAAGGTGTTCAGAGTAAGTCTGTGGCTGCAATTGCTGATAAACCATCA
CCGACTTTAACACCAACAACTTTCAAAAACACCAAAAAGGTTCTTGCCCCCAAAACTTCA
ACTATTACTCCTGAACTAGCAGCTGCAACTTCACCACCAAATCGAGTTCATCAGTCAACT
ATCAAAACCAACAAAAAGGCTGTTTCCACCAAAAATGTGAGTGAAGTACCAGCAACTTCA
CCTTCAAATCCAGTTCCTCAGACTATCAAGTGTTATCTATGTTATCCTGATAAAAGGAAC
ATAGTTGCTCGGGGTGAAATTCACTTATCATCAAAACGACAATCCATTCATGGAGTGCCT
TTGCAAGATGACTGCTACAAAGTGTCCATTTTGGAAATGGTGAAAAAGGATTCATATTTA
CCATATGAAACTGGTGAGATGAAAACAGTTGAGCAGGCTTTTAAATCTTTTGTCCCATGG
CCAAAGTACCTTGTCAAAGTTGCTGCAAAGGAAGCGGGTGTCAAACAAGTGGAAGTTAAT
GTGCCTGTTGTTCAACCATCTGGGAAACAGGTAGAAAACAATGTACAAGTTCTGACATCT
GCTGTGAAAGAAGTTGTGAAATCAAGTGTCAACGAAGTTGGGACTTCAAGTGTTAGGGAA
GTAGGAAAATCAAGTGTCATACCGCCTCAAGAGAATGTACCCCAAACCACAAACAAACGC
AAGGTTACTTACATACCCAATGGTGAACTTGCAAAAAGAACGAGGTCGTCACGTCCACTC

AATATGAAGTAG
>FGENESH: 27 10 exon (s) 142319 - 145353 623 aa, chain +
MENNNLLPDDVMYGDDAHLVYCTDEDDGDDSSSELEDGDDINEELEDVEDNNIHPEVDEV
EPGIVAAADIGSDAESDFNDSDQDNQLPRAQRPNRKRGMTRLPRLQTDYTRSGGRKKLVK
FDNLGRFSRKYRALFVSYLGDLVRQKHFFDVDDCQFKVVMKRMGMLLRNFRRKVANNVIA
KDEEPSRTLLWCKGREDKHGDIKPEAKMIADQLMEHEKQIKDGTVLLEPGTDAMTMVFGK
DKGGYLKGVGTEVTATTYFHIPRNKGSAKEEIKELKCAVQNGKVELEKKDAQVKALTTKF
DEQQETLNWVLAHLAATGTKIPNLPNTSGVSLDNVVESPATADLNVSTEPTEEAFTQAIK
RTNKKGVQSKSVAAIADKPSPTLTPTTFKNTKKVLAPKTSTITPELAAATSPPNRVHQST
IKTNKKAVSTKNVSEVPATSPSNPVPQTIKCYLCYPDKRNIVARGEIHLSSKRQSIHGVP
LQDDCYKVSILEMVKKDSYLPYETGEMKTVEQAFKSFVPWPKYLVKVAAKEAGVKQVEVN
VPVVQPSGKQVENNVQVLTSAVKEVVKSSVNEVGTSSVREVGKSSVIPPQENVPQTTNKR
KVTYIPNGELAKRTRSSRPLNMK
>FGENESH:[mRNA] 28 1 exon (s) 154491 - 154784 294 bp, chain +
ATGGTCGCCGGAGAAGGAAGAACAAGGGTGCTGCAGGTCGCCGGTGTGCAGCAAGGTTTG
TCTGCCGGCAGGAAGTCCTCAACAGCACGCAGCAGTGGGGCTGTAGCTGGAGGTTTAGCG
AGCAGGAGGGGGCAGGCCGTCGCCGGGAAGGGACTCGCCGGAACAGCAGGTCGTCACCGA
GATGGAGGAGTAGCAGCTGCGGCGCTTAGGGTTTCCTGCGGCCTGGTCGCGATCCACGAA
GAAGAAGGCAGATGTGCGGGAGATTTTAAAAAAAATCTGATCATGCTGCTTTGA
>FGENESH: 28 1 exon (s) 154491 - 154784 97 aa, chain +
MVAGEGRTRVLQVAGVQQGLSAGRKSSTARSSGAVAGGLASRRGQAVAGKGLAGTAGRHR
DGGVAAAALRVSCGLVAIHEEEGRCAGDFKKNLIMLL
>FGENESH:[mRNA] 29 1 exon (s) 159529 - 159735 207 bp, chain +
ATGTCGGGCTGGAATAGTGCAAATCGCCGGAAAACAGTAGGATTCCGCAGCAGCTTTCTC
ACTGCTGCTACTCACCGGGAAATCGCCGGAGAGGGTAAAGCTGGTGAACGAGATGGTCGC
CGGAGAAGGAAGAACAAGGGTGCTGCAGGTCGCCGGTGTGTAGCAAGGTTTGTCTGCCGG
CAGGAAGTCCTCAACAACACGCAGTAG
>FGENESH: 29 1 exon (s) 159529 - 159735 68 aa, chain +
MSGWNSANRRKTVGFRSSFLTAATHREIAGEGKAGERDGRRRRKNKGAAGRRCVARFVCR
QEVLNNTQ
>FGENESH:[mRNA] 30 1 exon (s) 165727 - 165873 147 bp, chain -
ATGGAAGGTGTGGCAGAAGTTCTACTTCCATTTTCAATAGAAAGGTTGCTCGAAGAGATG
TGTCTCCAGAAGGGTCTAGAGATGCCGGACGTTGCTGGCTCGGGAAGACTCTTTCGAAAA
TCAGCAAAGAGTCTGCCATTGATGTAA
>FGENESH: 30 1 exon (s) 165727 - 165873 48 aa, chain -
MEGVAEVLLPFSIERLLEEMCLQKGLEMPDVAGSGRLFRKSAKSLPLM
>FGENESH:[mRNA] 31 1 exon (s) 168965 - 169723 759 bp, chain +
ATGGGCCGGATCGGGTTTGGGTCAATCCGGGTCGGATCTGGCCGGGTTGGTCGGGTTGAT
CGAGTCAGTGCCGAGGTGATCGAGGTTTGCTCGGGCAAAGGAGTCGCGATCCGGGATTTG
TCCGAGGATGGCCGGGTTGCTGATCGAGGTTGCCGAGGATTGCCGAGTCGTTGCCGAGTG
GTGTCCGAGCAGGTCTGGTCGCGACTAGGAGTTGTCCGGGTAGTTGTCCGAGGTGTGCCG
AGGAGATCAGGTCGCTGCCGAGGAGTGCCGGGCACGTGTCCGAGAAGACATTTCGTCGAG
CAGGTGGTGTGCGCGGGTAAGTTGGTCGCGGTCCGAGGAGTTGCCGAGGATTTCCGGGTC
GTTGCCAAGCCGTGTGCGAGGAAGCAATACGTCGAACACTTGGTGTGCACGGTTGAGTTT
TCGTCGATTTTCGGCCAATTTGATCAGATTTTTGGCCGCAATCTCAATTATCGGCTGTTG
TTTGATGTTGAGGTGGTTTATCGATCGATTTTAGGCTTCAATTTCAGATTATCGGGGCTG
ATTTACCCGAACAAGTGGCCGACCAAACACGCACACAAGGTGTTCGCTGAAATGCCTGCC
GAGTCGCTGATCGGGGTGGCCGCGGATTGCTCGAGCAGACCTGGTCGCTGGTCCCGACGG

GGACTTTCGTCGAGCACCTGGCGTGCACTCGTCGATTATCGGCCGTTCTTCGTGATTTCC
GTCAATTTTTGGATGATTTCTTCTGTTTCTTGGTCGTAA
>FGENESH: 31 1 exon (s) 168965 - 169723 252 aa, chain +
MGRIGFGSIRVGSGRVGRVDRVSAEVIEVCSGKGVAIRDLSEDGRVADRGCRGLPSRCRV
VSEQVWSRLGVVRVVVRGVPRRSGRCRGVPGTCPRRHFVEQVVCAGKLVAVRGVAEDFRV
VAKPCARKQYVEHLVCTVEFSSIFGQFDQIFGRNLNYRLLFDVEVVYRSILGFNFRLSGL
IYPNKWPTKHAHKVFAEMPAESLIGVAADCSSRPGRWSRRGLSSSTWRALVDYRPFFVIS
VNFWMISSVSWS
>FGENESH:[mRNA] 32 1 exon (s) 171198 - 172043 846 bp, chain +
ATGAAAAAGCTTCATTCTGATTCAAAAGAAGAAAATTCACCTGATTTTGAGAAAATCTCA
TGTAAAAGTGAAGATACCGAAAGTGAATTGGAAGATATTGACTGTTCTGCTTTTGTAGGT
ACGAATTCAGTTGAAGGAAGTGCAGAAGTAGGAAATTTGTCTGAAAACAATGCTACTTTT
AATATTTGTAATAACGACAAGTTAAATGCCTTTAGGGCTAAAATTGCTTCTGATCTGAAA
GAAACTGCATGCGAACCACCTGTTTTTGAAACTGCATGCGAACCACCAGTATTTGAAACT
GCATGTAAAACACCTGAAAATGAACCTGCATGCGAACCACCTCAACCTGAAAATGTCATT
CCTGAACCTAAAATTGAAAATGTCCATCCTGAAGTAGTCAAAGTCAAGGACATAACTTAT
AAGACATTAAAGAAAGAAGGAAATTTAAGAGGTTATAACAATGAAAAACCATGTTCTGAT
AACCCTTCATGCTCAAATTCATCTGATGAGTCAAATAATTCAAATGTGAAGGCTGTTTTA
TTTCCCAAATTGAAAAACATACCAAATGCTTTGTTTGTTAAATCTGGATGTTCAAAAGCT
TCTACTTCTAGATTATCTTCGATTGTCATGAAAGAGAATTCTGACTGTTCTAGTGATGCT
ACTTATAGTTCAGATGGTTATAACAATAAAGATGATTGGAGAACTGAATTTAGATATGTT
TCAGGAAATTTGAACAAAAACATGAAACCTGAAAATGTAGAACAAACATATAGGCAAAGA
CAAAATCAAAATTACAAACGAAATTTGAGGACTAAAGAAAGAATTTCCAACAACAAAAGT
CCTTAG
>FGENESH: 32 1 exon (s) 171198 - 172043 281 aa, chain +
MKKLHSDSKEENSPDFEKISCKSEDTESELEDIDCSAFVGTNSVEGSAEVGNLSENNATF
NICNNDKLNAFRAKIASDLKETACEPPVFETACEPPVFETACKTPENEPACEPPQPENVI
PEPKIENVHPEVVKVKDITYKTLKKEGNLRGYNNEKPCSDNPSCSNSSDESNNSNVKAVL
FPKLKNIPNALFVKSGCSKASTSRLSSIVMKENSDCSSDATYSSDGYNNKDDWRTEFRYV
SGNLNKNMKPENVEQTYRQRQNQNYKRNLRTKERISNNKSP
>FGENESH:[mRNA] 33 1 exon (s) 172451 - 175870 3420 bp, chain +
ATGACTGGAAGAAAGGAAAAACTTCGGCATTTCCGAAAGCTGAAGGATGGTGGACGAGTG
AAGTTCGGAAACAATCATACTGCCGAAATCAAAGGATATGGACAAATTACAAACGACGAA
TTTACAATAAAACGAGTTGCTTATGTAGAAGGACTCAAACATAATTTAATCAGCGTATCT
CAACTTGTTGTAGGTACAGGATTAAAGATCACTTTTGATGATGAAGGTTCGGTGATTGAG
GACAAGAAAACCAAGAATGTAATTCTGAAATCCGAACGAAAAGGTGAAATGTTTCCATTG
GACATGAAACCCATCATTGGGAAGCCTTCTATTTGCCGGCTGACAAGAGCAGCCTCGGAC
ATGAGCTGGCTATGGCATAAAAGATTCTCTCACCTGAACTTCTGTGACATAAACAAACTT
GTGGTAAACGATCTCGTTCGTGGTTTACCTGTTCTTAAATTCGATAACGAACATCTCTGT
GCAGCTTGTGAGTTCGGAAAGCAAAGCAGGAAAAGTCATTCATCAATAGTTAATACCAAG
ATTGTGGAACCACTTGAATTGATTCACATCGATTTATGTGGGCCTTCCTCAATTCAAAGT
ATTGGTGGTAACAAATATTTACTTGTTATTGTTGATGATTTCTCACGTTTTACTTGGGTG
TATTTTCTTAAGAACAAATCCGAAACTACACAACAGATGATCGATTTTATCAAGTATGTT
GAACTGCAATTGCGAAAACCAGTTCGGAAGATCAGAAGTGACAATGGAACCGAATTCAAA
AATCACACGTTCGAAGCATTCTTAACAGAAAAGGGAATAGACCACAACTTTTCAGCACCA
AGAACACCTCAACAAAAAGGTGTAGTAGAGAGAAGTAACAGATCATTATGTGAAGCAGCT
CGATCTATGCTCAACTTCGCAGCTATACCTCTCTATTTTTGGGCTGAAGCTATTCTCTGT
GACTGTTTTACTCAAAACCGGACTTACATCAACAAAAGATTTTTAATCACACCATACGAA

ATCTTAAACAACAGGAAACCTAATGTTAAGTTTTTTCATGTATTTGGATCCAGGTGTTTT
CTATACAACACAAAGGACCAAAAGAACAAATTTCAAGCAAAAGCTGATGAAGTTATCTTC
CTTGGATACTCTCTACACTCAAAAGCATATAGGGTCCTAAACAAACAATCGAAAGTGATT
GAAGAAACATTTGATGTCACTTTTGATGAGGACTATATTAGGAAGAACAGAACTGTACAA
ATCGAATCAAATAAAATATTTCCCGAAAATCAGGTAGATTCGGAACCTTTAATGAATTTT
GAAGATGAATTTTCTTTGTTCTTTGATGAGCCAGTTAAAGCACTGGACTCCGAAGCTAGA
GCAAAAGATAACAAGCAAGACGAATTGTTAAAACTCATTGAAGAAACTGCTGCTGATTCG
AACCTTACTGAAGAACAAGAAACACCGAATCCTATTTTCGAGGGGGAGAGTGTGATTGAA
CAAATCATCTTTCCAGATCAAAATAACAATGATCAAGCCCAGGGGGAGAGTCATCAAACC
AATGACACGGATGAAGTTCCAGTCCAGGGGGAGAGAGTTCAAACATGTAGTTCGGAATCT
GCTGATGATATGGGTTATGCGTCAAATTCGGATACAAGAATCAATTCCGAAGCTGAAGAA
GTCATACCTCTGAATGAAAATGATTACGACATGAATTATCCACCTCTTGTAAAATGGACA
AGGGATCATCCACAAAGACAAATCATTGGTTCACCATCACAAGCAATCCTAACTCGAGCT
CAGAGAAAAGAAAGAGAAGCTACTTTGAATAAAAATCTTTTATTTTGTCAACATAATGCT
TTTCTCTCTAAAATCGAACCTAAAAATGTTAAAATTGCTCTTGACCATTCTGATTGGGTT
GAAGCAATGCAGGCTGAGTTAAACGAGTTTGAGAGAAACAAAGTTTGGAGGCTAATTCCC
ACTCCACCAGATGTTTCGGTTGTAGGCCTAAAATGGGTCTTCCGAAACAAAGTTGATAAA
GAAGGGAATGTTGTTCGAAACAAGGCTAGACTGGTCGTTAAAGGATACTGTCAACAAGAA
GGTATAGACTACGAAGAGACATTTGCTCCAGTTGCAAGACTCGAAGCAGTTCATATCTTC
TTAGCCTATGCTACAAGCAAGAACTTCCAAGTATATCAAATGGATGTGAAGTGTGCTTTT
CTTAATGTCGAACTTGAAGAAACAGTCTACGTCGAACAACCACCAGGTTTTGTTAATGAG
AAATTTCCTAACCATTGCTACATTCTAGACAAAGCTGTCTACGGTCTTAAACAAGCACCA
CGAGCATGGTATGAGACTCTAACAAAATTTCTTAAGTTATCTAATTTTAAACAAGGTCCA
GTTGATCCAACATTATTTCGTAAAAAGGTTGGGGATCACTTAATGATGGTTCAGATTTAT
GTTGATGACATAATATTTGGTTCTACAGATCCGAAACTGACTGTGGAATTCAAAGCTCTT
ATGGAAACTAAATTTGAAATGTCATCAATGGGTCCTATTAACTTTTTCCTTGGATTGAAT
GTAGTTCAGAATAGCGCAGGAGTGTTTATCAATCAAGAAGCCTTTACGAAGAAGCTTCTC
ATCAAGTTCGGAATGACAGGAGGAAGCAAAGCCAAAGTTCTTATGGCATTCGGAACAAAG
TTGAAACCCTCGCTTGACGAACCAGCTGCAGACCAAACACTTTACAGGGGCATGATAGGA
TCACTTTTATACTTAACTTCTAGTAGACCTGATATCATGTTTGCTGTCTGTTATTGTGCA
AGGTATCAATCGAATCCACGTACATCTCACATGACTGCTGTGAAGAACATCTTCAGATAC
TTGCAAAACACAATCTCGCTTGGAATTTGGTATCCTGCGAACACAGGATTCTTCGTACAA
GCATATACAGATTCGGACCTAGGAGGTTGCAACCTTGATCTTAAGAGTACCTCAGGCGGA
TGTCAGTTCTTGGACCGAAAGCTAGTAAGCTGGCAATCGAGAAAACAAACGTGTGTTTCT
CTTTCCACTGCCGAAGCAGAATACATTGCCACTGCTACTTGTACATCACAAGTCCTTTGG
ATTCAAAGCCAACTTCGAGACTATGGGGTGAACATGAAGAAAATTCCAATATACTGTGAT
TCCGAAAGTACGATTCGGATTTGTCACAACCCTGTACAACACTCGAAAACAAAGCACATC
GCTCTGAGATACCATTTTATCAAAGATCATATCGAAGAAGGAAACATCGAGATACACTTT
GTGAAAACAACTGAGCAACTAGCTGACATATTCACAAAGGCCTTAGCTGAGATCCCATAC
ATGAACATTCTAAGGGGACTCGGAATGATGGAAGCTCACAATGTTCCGTCATCCAGCTAA
>FGENESH: 33 1 exon (s) 172451 - 175870 1139 aa, chain +
MTGRKEKLRHFRKLKDGGRVKFGNNHTAEIKGYGQITNDEFTIKRVAYVEGLKHNLISVS
QLVVGTGLKITFDDEGSVIEDKKTKNVILKSERKGEMFPLDMKPIIGKPSICRLTRAASD
MSWLWHKRFSHLNFCDINKLVVNDLVRGLPVLKFDNEHLCAACEFGKQSRKSHSSIVNTK
IVEPLELIHIDLCGPSSIQSIGGNKYLLVIVDDFSRFTWVYFLKNKSETTQQMIDFIKYV
ELQLRKPVRKIRSDNGTEFKNHTFEAFLTEKGIDHNFSAPRTPQQKGVVERSNRSLCEAA
RSMLNFAAIPLYFWAEAILCDCFTQNRTYINKRFLITPYEILNNRKPNVKFFHVFGSRCF
LYNTKDQKNKFQAKADEVIFLGYSLHSKAYRVLNKQSKVIEETFDVTFDEDYIRKNRTVQ

IESNKIFPENQVDSEPLMNFEDEFSLFFDEPVKALDSEARAKDNKQDELLKLIEETAADS
NLTEEQETPNPIFEGESVIEQIIFPDQNNNDQAQGESHQTNDTDEVPVQGERVQTCSSES
ADDMGYASNSDTRINSEAEEVIPLNENDYDMNYPPLVKWTRDHPQRQIIGSPSQAILTRA
QRKEREATLNKNLLFCQHNAFLSKIEPKNVKIALDHSDWVEAMQAELNEFERNKVWRLIP
TPPDVSVVGLKWVFRNKVDKEGNVVRNKARLVVKGYCQQEGIDYEETFAPVARLEAVHIF
LAYATSKNFQVYQMDVKCAFLNVELEETVYVEQPPGFVNEKFPNHCYILDKAVYGLKQAP
RAWYETLTKFLKLSNFKQGPVDPTLFRKKVGDHLMMVQIYVDDIIFGSTDPKLTVEFKAL
METKFEMSSMGPINFFLGLNVVQNSAGVFINQEAFTKKLLIKFGMTGGSKAKVLMAFGTK
LKPSLDEPAADQTLYRGMIGSLLYLTSSRPDIMFAVCYCARYQSNPRTSHMTAVKNIFRY
LQNTISLGIWYPANTGFFVQAYTDSDLGGCNLDLKSTSGGCQFLDRKLVSWQSRKQTCVS
LSTAEAEYIATATCTSQVLWIQSQLRDYGVNMKKIPIYCDSESTIRICHNPVQHSKTKHI
ALRYHFIKDHIEEGNIEIHFVKTTEQLADIFTKALAEIPYMNILRGLGMMEAHNVPSSS
>FGENESH:[mRNA] 34 1 exon (s) 177853 - 180078 2226 bp, chain +
ATGGCTACCCAAAAACCTCAACAAAACCCAGAGGCATCGGAACAATTTTCATTCCCAGAT
GACACAGGATCATCGTTTCCTGTACTCGATATCAAAAATAACAACCTATACCTTAATCTG
GATACAGTACTTCAACCAGGTGCTAAATTCTTTGATTCATTTTTCAAATCCATTTTTGTC
TGCCTCATGCACTCAAGAATATCTACAGCCCTAACCCTTACCAAATCCGTACCCATCTCT
GTTCTTTCAAGAGCTTATGCAACCGCACGATATGACAAACCCAGCGAATCGATGCATTTT
GATCTTGCAACCAACAAATCCACGTCAATTACCAAACATCATTTCTGTAAATTGCTCAAT
CTTCAGTCCTCCACAGACCTAACTCATCCAGACTCCATATCAAATGTTGATTTGATAAAC
ATGTGTAACCAAATGGGGCACGAACCGTTATTGGAAACTGTATCGAAGATGAACAAATCA
CGGATGCCGCCGACATGGAACCTGTTAGCATTGATCATTCTTCGGTGTTTTGCTGAACGA
ACTACTGGTTCGGATAATTCTAGTAAGTTGCTCCTCACTCTCATCTACGCCATTTATTCA
AATCAAAACATCGATATAGGTCAGATTTTATGGACTCAATTCTGTCTGAGCCCTAATTCT
AGTTCCCGAACGACAAAAATTTCCATGGCTCGATTTTGGGCTATTGTGGTTGATGGGGCT
CTGAACAAGTTCACAAACCTAAGAGGAGATAAAGATACAGCTATGGTTGAACTTTCGGAA
CTACAAGTTAATAAACTTCAATTTGTTAAGGAACGTGTTTTCGAACATTGTGGTGAAATC
CCTTCTGAAATGTGGTCAATTGTTCCTGAGGATGATCCTCAGAAGAAGAGGGTAAAGAAA
GCTAACAACGGGGTTCTACCTGAAGTTGTTCTTCGGGACATACCATTGGATGTGCAGGAA
AGGGTTGATCTGAACTACACAAAGAAACCCCAAACTAAACGCAAAAAGGGAGAAACCATT
GAACCTGAACCTGTGAACGAGCCGGATCAATCCCCTCCGAAGAAGAAAAAGAAAATAAAG
AAGGCAGCTCGGAAACCTTCTAAGAAACCAAAGAAAACCCCAACTCTTCAAGATGAACCA
ACTGATGATGAGGAAGCTACTCAGTCAAATGCTTTTCATCACTCCGAACCAGAACATCAA
CCGAACCAAGGAGAAGGTACTTCGAAATCCCCTCCCAAACAAACAAAAAAAGACACTACC
TTCGACAACCTTGAAAACATTGAAAACCTTACCCAAACACCTCCCGTACCACCTGAACAT
ACCGAACATCATGATGAAACAAACAAACCCTCACCACATCAATCCGAATCTGAACATGTT
GATGAGGATATAATTTTGGAAGGACCTGGAGGAGACATGTTCAATCCGAATCAAGCCACC
TCTTCCACATATGTCAAACCAACAGACATGTTCACAATTCTTCCTGTAACATTCGAAGAA
GAAGACTTAACCGCCGAACAACTAAATCTTTCAGCCATAAAGAAGGACATCTCTTCTTTG
CGAACCATCCTGAACACTATCCTTGTAGGTATTGATCCTTCTTCTGCCAATCAAAAAGCT
GATTCTGCAAAGAAACAAACAGAAGAGTTTCAGGAGATTCGAAAAGAACTCTTAACCTCT
ATCGATACTCTCCAAGCTGATTTCACATCCAAGATTTCGGATGTTGAAAAGAAAATCGAT
GAAGTGTCTAAAACAGCTACAACTGAGACAGAGTTGAAGGCCAAAGTGCACGCTCTGGAA
CTCCAAATTCAAAAACTTGAGACTCAACTGGAGTCTAAAACCCATGAAGCTGACCATAGT
CTGAGAGTTATCGATATGTATAAAGCTCAGAATCAGGAGATGAACCTGAAACTTGTCAAG
CTCGTTGAAGACAAGGATCATCAAGTTCAGAAGCTATCGGAGAAAGTTGATTCGAAATTT
GATCAAGTGATTAAAGCCATTTCAGAGATTCAAATTCCGAAAGTGGTTGAGAAGGTTGTG
GAAAAGGTTTTGAGAAGCCAGGGGCTCATTCTCAAGAAGTGGGAGATAATAGAGAAAGTG

AACAACCACAAAAGAATCAAACTGAAACCGAAACAACCAATAAACCGCCTCCAAAATCAC
CTCCGAAACAGTCCAAGAAAACACCACCAAAGAAATCAAAATGACCACCGGTTAAAGGTG
TTGTAA
>FGENESH: 34 1 exon (s) 177853 - 180078 741 aa, chain +
MATQKPQQNPEASEQFSFPDDTGSSFPVLDIKNNNLYLNLDTVLQPGAKFFDSFFKSIFV
CLMHSRISTALTLTKSVPISVLSRAYATARYDKPSESMHFDLATNKSTSITKHHFCKLLN
LQSSTDLTHPDSISNVDLINMCNQMGHEPLLETVSKMNKSRMPPTWNLLALIILRCFAER
TTGSDNSSKLLLTLIYAIYSNQNIDIGQILWTQFCLSPNSSSRTTKISMARFWAIVVDGA
LNKFTNLRGDKDTAMVELSELQVNKLQFVKERVFEHCGEIPSEMWSIVPEDDPQKKRVKK
ANNGVLPEVVLRDIPLDVQERVDLNYTKKPQTKRKKGETIEPEPVNEPDQSPPKKKKKIK
KAARKPSKKPKKTPTLQDEPTDDEEATQSNAFHHSEPEHQPNQGEGTSKSPPKQTKKDTT
FDNLENIENLTQTPPVPPEHTEHHDETNKPSPHQSESEHVDEDIILEGPGGDMFNPNQAT
SSTYVKPTDMFTILPVTFEEEDLTAEQLNLSAIKKDISSLRTILNTILVGIDPSSANQKA
DSAKKQTEEFQEIRKELLTSIDTLQADFTSKISDVEKKIDEVSKTATTETELKAKVHALE
LQIQKLETQLESKTHEADHSLRVIDMYKAQNQEMNLKLVKLVEDKDHQVQKLSEKVDSKF
DQVIKAISEIQIPKVVEKVVEKVLRSQGLILKKWEIIEKVNNHKRIKLKPKQPINRLQNH
LRNSPRKHHQRNQNDHRLKVL
